# NADPH oxidase 2 mediates cardiac sympathetic denervation and myocyte autophagy, resulting in cardiac atrophy and dysfunction in doxorubicin-induced cardiomyopathy

**DOI:** 10.1038/s41598-024-57090-2

**Published:** 2024-03-23

**Authors:** Yuan Ma, Hui-Ping Zhao, Li-Guo Yang, Lu Li, Ai-Lin Wang, Xiao-Juan Zhang, Ke Wang, Bin Yang, Zong-Feng Zhu, Pei-Jun Zhang, Jia-Pu Wang, Rui-Fang Chi, Bao Li, Fu-Zhong Qin, Zhi-Peng Wang

**Affiliations:** 1 The Second Hospital of Shanxi Medical University, 382 Wuyi Road, Taiyuan, 030001 Shanxi People’s Republic of China; 2 Shanxi Medical University, Taiyuan, 030001 Shanxi People’s Republic of China; 3 Shanxi Datong University School of Medicine, Datong, 037009 Shanxi People’s Republic of China; 4 Institute for Radiation Protection, Taiyuan, 030006 Shanxi People’s Republic of China

**Keywords:** Cardiomyopathies, Heart failure

## Abstract

Doxorubicin has been used extensively as a potent anticancer agent, but its clinical use is limited by its cardiotoxicity. However, the underlying mechanisms remain to be fully elucidated. In this study, we tested whether NADPH oxidase 2 (Nox2) mediates cardiac sympathetic nerve terminal abnormalities and myocyte autophagy, resulting in cardiac atrophy and dysfunction in doxorubicin-induced heart failure. Nox2 knockout (KO) and wild-type (WT) mice were randomly assigned to receive a single injection of doxorubicin (15 mg/kg, i.p.) or saline. WT doxorubicin mice exhibited the decreases in survival rate, left ventricular (LV) wall thickness and LV fractional shortening and the increase in the lung wet-to-dry weight ratio 1 week after the injections. These alterations were attenuated in Nox2 KO doxorubicin mice. In WT doxorubicin mice, myocardial oxidative stress was increased, myocardial noradrenergic nerve fibers were reduced, myocardial expression of PGP9.5, GAP43, tyrosine hydroxylase and norepinephrine transporter was decreased, and these changes were prevented in Nox2 KO doxorubicin mice. Myocyte autophagy was increased and myocyte size was decreased in WT doxorubicin mice, but not in Nox2 KO doxorubicin mice. Nox2 mediates cardiac sympathetic nerve terminal abnormalities and myocyte autophagy—both of which contribute to cardiac atrophy and failure after doxorubicin treatment.

## Introduction

The anthracycline antibiotic doxorubicin has been used extensively as a potent anticancer chemotherapeutic agent, but its clinical use is limited by its cardiotoxicity^1^. Doxorubicin-induced cardiotoxicity is characterized by eccentric ventricular hypertrophy, dilated cardiomyopathy and congestive heart failure^2,3^. The heart is extensively innervated by sympathetic nerves^4^. A number of studies have shown that cardiac sympathetic nerve terminals are reduced in heart failure^5–7^. Doxorubicin-induced cardiomyopathy is associated with cardiac sympathetic terminal abnormalities^8–10^, as evidenced by decreased protein gene product (PGP) 9.5 and tyrosine hydroxylase, markers of cardiac innervation. Cardiac sympathetic nerves provide trophic signal to the heart^4^. Recently, clinical and experimental animal studies have demonstrated that doxorubicin induces cardiac atrophy, reflective of reduced left ventricular (LV) mass, LV function and cardiomyocyte size^3,11^. However, whether doxorubicin-induced cardiac atrophy is associated with cardiac sympathetic terminal abnormalities remains to be completely understood. Evidence has accumulated that oxidative stress contributes to doxorubicin-induced cardiotoxicity^12,13^. NADPH oxidase is a major source of reactive oxygen species (ROS) in heart failure^14^. NADPH oxidase 2 (Nox2) is a major subunit of NADPH oxidase^15^. In the present study, we first tested the hypothesis that Nox2 mediates cardiac sympathetic nerve terminal abnormalities, resulting in cardiomyocyte atrophy in doxorubicin-induced heart failure.

The size of cardiomyocytes is regulated by the relative rate of protein synthesis and degradation^4,16^. Autophagy plays an important role in maintaining cardiomyocyte size and myocardial structure and function^17,18^. Insufficient and excessive levels of autophagy contribute to myocardial remodeling and dysfunction^17^. Doxorubicin has been shown to increase cardiomyocyte autophagy^19,20^ and myocardial oxidative stress^13,21^. Oxidative stress induces cardiomyocyte autophagy^22,23^. However, it remains unknown whether Nox2-derived ROS mediate doxorubicin-induced cardiomyocyte autophagy, contributing to cardiac atrophy and failure. To address this question, in this study, secondly, we examined the effect of Nox2 deficiency on cardiomyocyte autophagy in doxorubicin-induced cardiomyopathy.

## Results

### Nox2 deficiency improves survival rate and prevents cardiac atrophy and LV dysfunction in doxorubicin-induced cardiomyopathy

**Figure 1:**
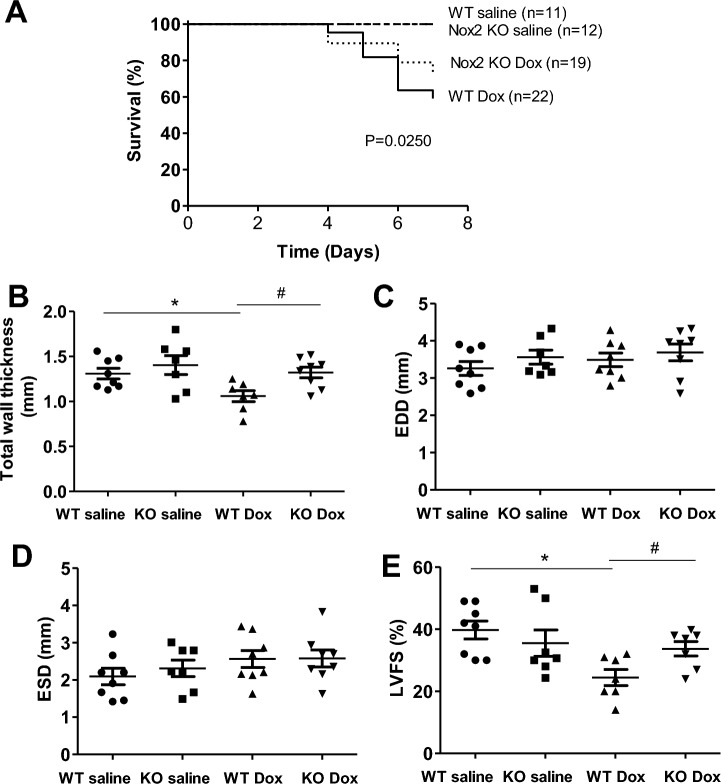
(**A**) Survival analysis through 7 days when mice were euthanized. Survival was calculated by Kaplan–Meier analysis. (**B**–**E**) Changes in left ventricular (LV) total wall thickness, LV end-diastolic dimension, LV end-systolic dimension and LV fractional shortening in WT and Nox2 KO mice with saline or doxorubicin (Dox) treatment. Values are presented as mean ± S.E.M.; n = 7–8. *P < 0.05 versus WT saline group; #*P* < 0.05 WT Dox group. Comparisons among groups were performed by one-way ANOVA followed by a Bonferroni post hoc test for multiple comparisons.

During the 1-week period after doxorubicin injection, nine mice died in the WT doxorubicin group (n = 22), five mice died in the Nox2 KO doxorubicin group (n = 19). The survival rate was 59.1% in the WT doxorubicin group, and was increased to 73.7% in the Nox2 KO doxorubicin group (Fig. 1A). None of the mice died in the WT saline group (n = 11) and Nox2 KO saline group (n = 12). The survival rate was 100% in the WT saline or Nox2 KO saline group (Fig. 1A). The results suggest that Nox2 deficiency significantly improves the survival rate in doxorubicin-induced cardiomyopathy in mice.

Figure 1B–E shows echocardiographic data of all experimental groups. The WT doxorubicin mice exhibited a decrease in total LV wall thickness (anterior + posterior wall thickness at diastole) that was prevented in the Nox2 KO doxorubicin mice (Fig. 1B). There were no statistical differences in LV EDD and LV ESD among the groups (Fig. 1C,D). In the WT doxorubicin group, LV FS was decreased, and the decrease was attenuated in the Nox2 KO doxorubicin group (Fig. 1E). These findings suggest that Nox2 mediates doxorubicin-induced cardiac atrophy and dysfunction.

Table 1 summarizes body and organ weights of all experimental groups. Body weight and tibial length did not differ statistically among the groups. Total heart weight and the ratio of total heart weight to tibial length were decreased in the WT doxorubicin group, reflective of cardiac atrophy, and the decreases were attenuated in the Nox2 KO doxorubicin group. The lung wet-to-dry weight ratio was increased in the WT doxorubicin group, indicative of lung congestion, and the increase was attenuated in the Nox2 KO doxorubicin group. There was no significant difference in the liver wet-to-dry weight ratio among the groups. These data further suggest that Nox2 mediates doxorubicin-induced cardiac atrophy and failure.

**Table 1:** Organ weights.

	WT saline	Nox2 KO saline	WT Dox	Nox2 KO Dox
BW (g)	24.4 ± 1.1	25.4 ± 1.1	23.5 ± 1.2	22.9 ± 1.0
TL (mm)	17.7 ± 0.2	17.8 ± 0.2	17.7 ± 0.1	17.9 ± 0.1
THW (mg)	119.2 ± 4.1	121.3 ± 4.7	98.9 ± 3.7	105.0 ± 7.6
THW/TL (mg/mm)	6.08 ± 0.27	6.29 ± 0.20	4.71 ± 0.16*	5.80 ± 0.34#
Liver W (wet/dry)	3.06 ± 0.10	3.21 ± 0.09	3.15 ± 0.11	3.20 ± 0.07
Lung W (wet/dry)	4.35 ± 0.11	4.17 ± 0.09	4.96 ± 0.22*	4.08 ± 0.09#

Values are mean ± S.E.M. N = 9–14.

*WT* wild type, *KO* knockout, *Dox* doxorubicin, *BW* body weight, *TL* tibial length, *THW* total heart weight, *W* weight.

*P < 0.05 versus WT saline group. #*P* < 0.05 versus WT Dox group.

### Nox2 deficiency attenuates myocardial oxidative stress in doxorubicin-induced cardiac atrophy and failure

**Figure 2:**
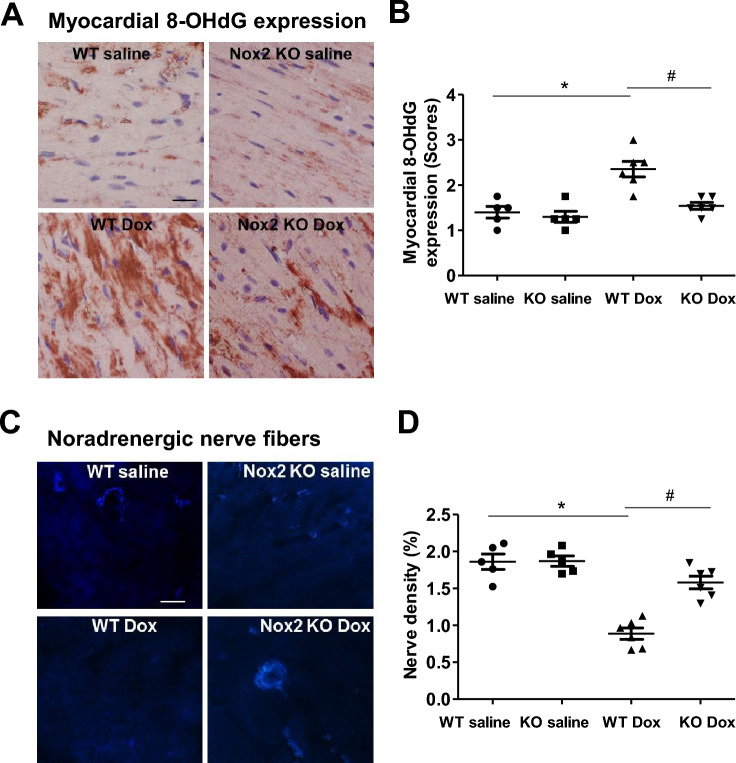
(**A**) Immunohistochemical staining for 8-hydroxydeoxyguanosine (8-OHdG) in left ventricular myocardium of WT and Nox2 KO mice with saline or Doxorubicin (Dox) treatment. 8-OHdG staining is shown in red. Nuclie are shown in blue. Bar = 20 μm. (**B**) The graph shows the relative expression of myocardial 8-OHdG in the four groups. Values are presented as means ± S.E.M.; n = 5–6. *P < 0.05 versus WT saline group. #*P* < 0.05 versus WT Dox group. (**C**) Sucrose-potassium phosphate-glyoxylic acid (SPG)-induced histofluorescence for myocardial noradrenergic nerve fibers. Bar = 10 μm. (**D**) The graph shows the relative noradrenergic nerve density in the four groups. Values are presented as means ± S.E.M.; n = 5–6. *P < 0.05 versus WT saline group. #*P* < 0.05 versus WT Dox group. Comparisons among groups were performed by one-way ANOVA followed by a Bonferroni post hoc test for multiple comparisons.

Figure 2A,B shows the immunohistochemical staining of 8-OHdG, an index of oxidative stress^24^. As expected, myocardial 8-OHdG expression was increased in the WT doxorubicin group compared with the WT saline group, but not in the Nox2 doxorubicin group (Fig. 2A,B), supporting the notion that Nox2 NADPH oxidase-derived ROS play an important role in doxorubicin-induced cardiac atrophy and failure.

### Nox2 deficiency attenuates reduced cardiac sympathetic nerve terminal density in doxorubicin-induced cardiac atrophy and failure

**Figure 3:**
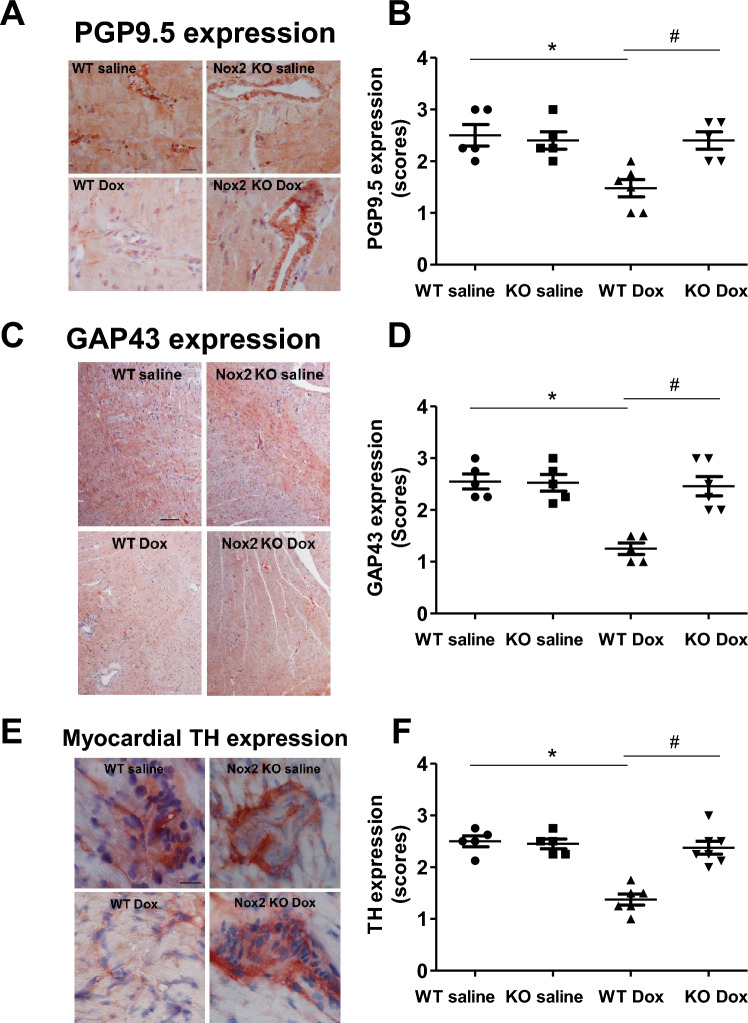
Immunohistochemical staining for PGP9.5, GAP43 and tyrosine hydroxylase (TH) in left ventricular myocardium of WT and Nox2 KO mice with saline or Doxorubicin (Dox) treatment. (**A**) PGP9.5. Bar = 20 μm. (**B**) The graph shows the relative expression of myocardial PGP9.5 in the four groups. Values are presented as means ± S.E.M.; n = 5–6. *P < 0.05 versus WT saline group. #*P* < 0.05 versus WT Dox group. (**C**) GAP43. Bar = 100 μm. (**D**) The graph shows the relative expression of myocardial GAP43 in the four groups. Values are presented as means ± S.E.M.; n = 5–6. *P < 0.05 versus WT saline group. #*P* < 0.05 versus WT Dox group. (**E**) TH. Bar = 20 μm. (**F**) The graph shows the relative expression of myocardial TH in the four groups. Values are presented as means ± S.E.M.; n = 5–7. *P < 0.05 versus WT saline group. #*P* < 0.05 versus WT Dox group. Comparisons among groups were performed by one-way ANOVA followed by a Bonferroni post hoc test for multiple comparisons.

Oxidative stress and cardiac sympathetic nerve damage coexist in doxorubicin-induced cardiomyopathy^9,10,25^. We examined whether Nox2 NADPH oxidase-derived ROS mediate myocardial sympathetic nerve terminal abnormalities in doxorubicin-induced heart failure. The sympathetic nerve terminal markers were assessed by catecholaminergic histofluorescence and the immunohistochemical expression of PGP9.5, GAP43 and tyrosine hydroxylase, a key enzyme in the synthesis pathway of catecholamines^7,26^. Figure 2C shows the representative catecholaminergic histofluorescence. Myocardial noradrenergic nerve fibers were reduced in the WT doxorubicin group compared with the WT saline group, and the reduction was attenuated in the Nox2 KO doxorubicin group (Fig. 2C, D). Figure 3A–F shows that myocardial PGP9.5, GAP43 and tyrosine hydroxylase immunohistochemical expression was decreased in the WT doxorubicin group compared with the WT saline group. These changes were ameliorated in the Nox2 KO doxorubicin group (Fig. 3A–F). These results suggest that Nox2 NADPH oxidase-derived ROS mediate myocardial sympathetic nerve terminal abnormalities in doxorubicin-induced heart failure.

### Nox2 deficiency restores protein expression levels of PGP9.5, GAP43, tyrosine hydroxylase and NET in doxorubicin-induced cardiac atrophy and failure

**Figure 4:**
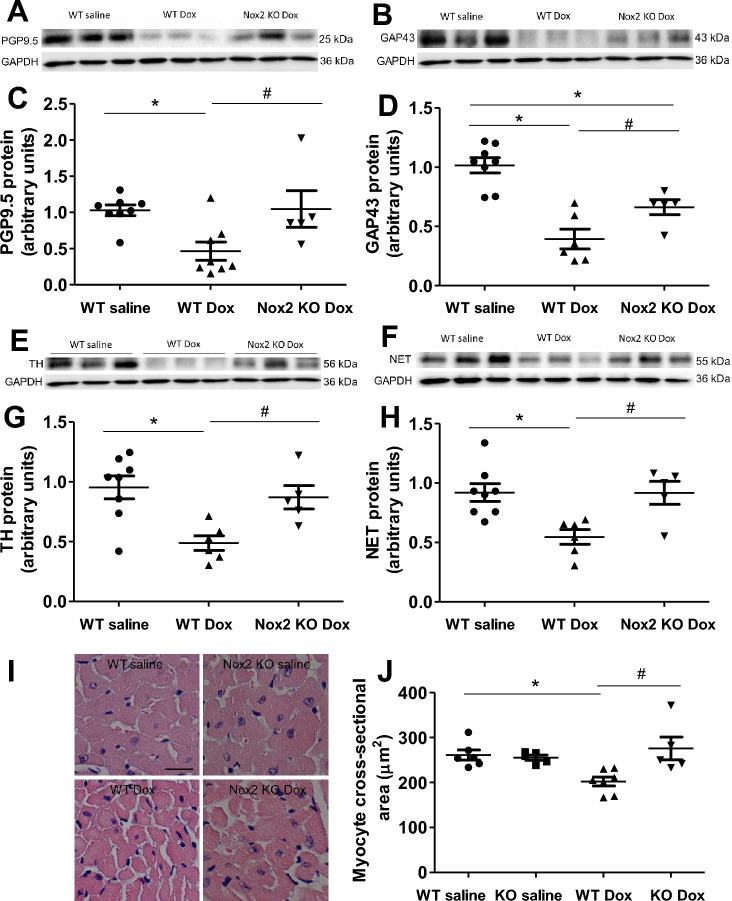
(**A**–**H**) Changes in myocardial PGP9.5, GAP43, tyrosine hydroxylase (TH) and noradrenaline transporter (NET) proteins in WT saline, WT doxorubicin (Dox) and Nox2 KO Dox groups. (**A**, **B**, **E**, **F**) Representative Western blots of PGP9.5, GAP43, TH and NET proteins, respectively. Equal loading of proteins is illustrated by GAPDH bands. (**C**, **D**, **G**, **H**) The graphs show the relative expression of PGP9.5, GAP43, TH and NET in the three groups, respectively. Values are presented as means ± S.E.M.; n = 5–8. *P < 0.05 versus WT saline group. #*P* < 0.05 versus WT Dox group. (**I**, **J**) Changes in myocardial cross-sectional area in left ventricular myocardium of WT and Nox2 KO mice with saline or Doxorubicin (Dox) treatment. (**I**) The representative photomicrographs of left ventricular myocardium stained by hematoxylin and eosin. Bar = 20 μm. (**J**) The graph shows the mean myocyte cross-sectional area measured by ImageJ. Values are presented as means ± S.E.M.; n = 5–7. *P < 0.05 versus WT saline group. #*P* < 0.05 versus WT Dox group. Comparisons among groups were performed by one-way ANOVA followed by a Bonferroni post hoc test for multiple comparisons.

Figure 4 shows the protein expression levels of PGP9.5, GAP43, tyrosine hydroxylase and noradrenaline transporter (NET) by Western blot. The protein expression levels of PGP9.5, GAP43, tyrosine hydroxylase and NET were reduced in the WT doxorubicin group compared with the WT saline group (Fig. 4A–H). Nox2 deficiency had no effect in the saline-treated group (Supplementary Fig. 1A–H), but prevented the reduction in the protein expression levels of PGP9.5, GAP43, tyrosine hydroxylase and NET in the doxorubicin-treated group (Fig. 4A–H). These findings further suggest that Nox2 NADPH oxidase-derived ROS mediate the decreases in myocardial sympathetic nerve terminal density, norepinephrine synthesis and norepinephrine uptake in doxorubicin-induced heart failure.

### Nox2 deficiency attenuates myocyte atrophy and improves ERK and Akt signaling in doxorubicin-induced heart failure

Studies have shown that cardiac sympathetic denervation induced by 6-hydroxy-dopamine is associated with a significant reduction in cardiac size^4^. Doxorubicin reduces cardiac mass^3,27,28^. In consistent with these findings about LV total wall thickness and heart weight, myocyte size assessed by myocyte cross-sectional area was markedly reduced in the WT doxorubicin group, the reduction was prevented in the Nox2 KO doxorubicin group (Fig. 4I,J).

**Figure 5:**
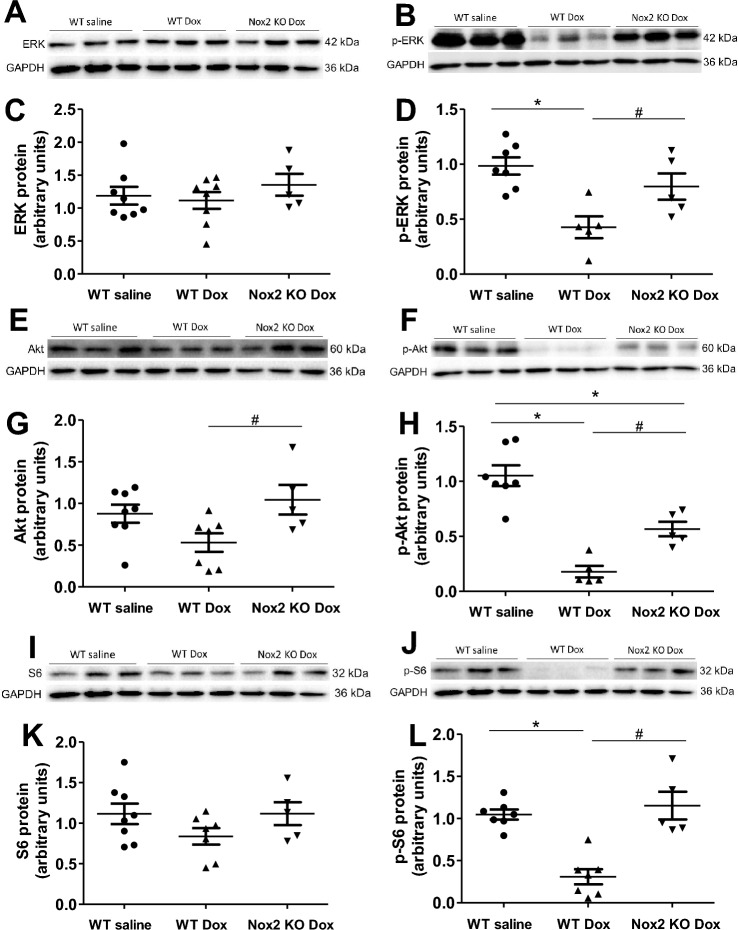
(**A**–**L**) Changes in myocardial ERK, p-ERK, Akt, p-Akt, S6 and p-S6 proteins in WT saline, WT doxorubicin (Dox) and Nox2 KO Dox groups. (**A**, **B**, **E**, **F**, **I**, **J**) Representative Western blots of ERK, p-ERK, Akt, p-Akt, S6 and p-S6 proteins, respectively. Equal loading of proteins is illustrated by GAPDH bands. (**C**, **D**, **G**, **H**, **K**, **L**) The graphs show the relative expression of ERK, p-ERK, Akt, p-Akt, S6 and p-S6 proteins in the three groups, respectively. Values are presented as means ± S.E.M.; n = 5–8. *P < 0.05 versus WT saline group. #*P* < 0.05 versus WT Dox group. Comparisons among groups were performed by one-way ANOVA followed by a Bonferroni post hoc test for multiple comparisons.

Evidence has accumulated that myocyte size is regulated by the relative rate of protein synthesis and degradation^4,29^. We assessed the protein expression levels of ERK and Akt/S6 signaling pathways. Figure 5A,C shows that total ERK protein level did not change among the groups. Figure 5E,G,I and K shows that total Akt and S6 protein levels tended to decrease in the WT doxorubicin group and the decreases were attenuated in the Nox2 KO doxorubicin group. Phosphorylated-ERK (p-ERK), phosphorylated Akt (p-Akt) and phosphorylated S6 (p-S6) protein levels, indicative of ERK, Akt and S6 activity, were markedly decreased in the WT doxorubicin group (Fig. 5B,D,F,H,J and L), suggesting reduced protein synthesis, and Nox2 deficiency prevented the decreases in p-ERK, p–Akt and p-S6 protein levels (Fig. 5B,D,F,H,J and L). There were no statistically significant differences in these parameters between the WT saline and Nox2 KO saline groups (Supplementary Fig. 2A–G). These data suggest that Nox2 mediates cardiac myocyte atrophy in doxorubicin-induced heart failure, at least in part, through ERK and Akt/S6 signaling pathways.

### Nox2 deficiency prevents activation of myocyte autophagy in doxorubicin-induced cardiac atrophy and failure

**Figure 6:**
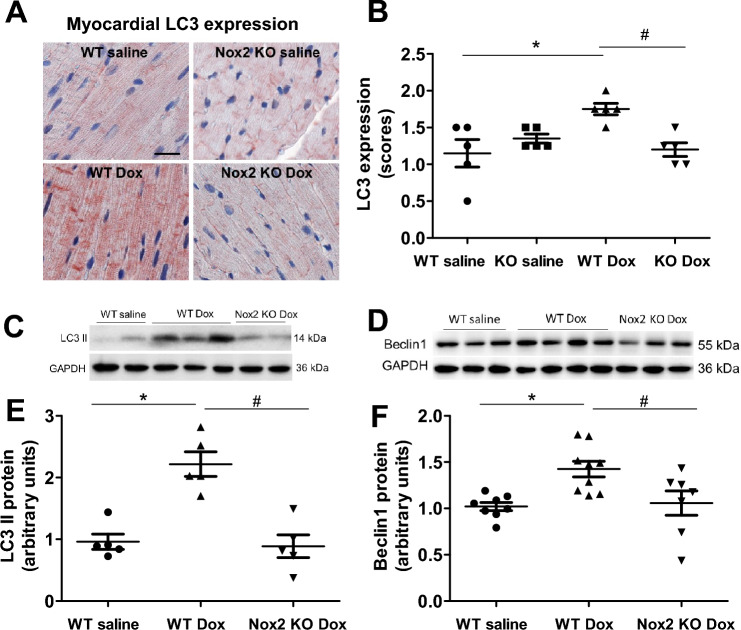
(**A**) Immunohistochemical staining for LC3 in left ventricular myocardium of WT and Nox2 KO mice with saline or Doxorubicin (Dox) treatment. LC3 staining is shown in red. Nuclie are shown in blue. Bar = 20 μm. (**B**) The graph shows the relative expression of myocardial LC3 in the four groups. Values are presented as means ± S.E.M.; n = 5. *P < 0.05 versus WT saline group. #*P* < 0.05 versus WT Dox group. (**C**–**F**) Changes in myocardial LC3 II and Beclin1 proteins in WT saline, WT doxorubicin (Dox) and Nox2 KO Dox groups. (**C**, **D**) Representative Western blots of LC3 II and Beclin1 proteins, respectively. Equal loading of proteins is illustrated by GAPDH bands. (**E**, **F**) The graphs show the relative expression of LC3 II and Beclin1 proteins in the three groups, respectively. Values are presented as means ± S.E.M.; n = 5 for (**E**), n = 7–9 for (**F**). *P < 0.05 versus WT saline group. #*P* < 0.05 versus WT Dox group. Comparisons among groups were performed by one-way ANOVA followed by a Newman–Keuls (**B**) or Bonferroni post hoc test (**E**, **F**) for multiple comparisons.

**Figure 7:**
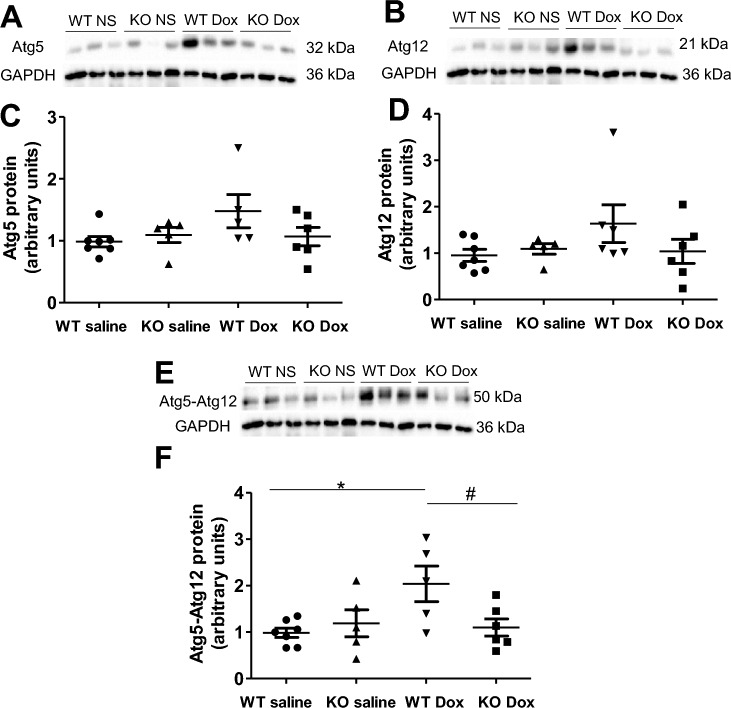
(**A**–**F**) Changes in myocardial Atg5, Atg12 and Atg5-Atg12 proteins in WT and Nox2 KO mice with saline (NS) or doxorubicin (Dox) treatment. (**A**, **B**, **E**) Representative Western blots of Atg5, Atg12 and Atg5-Atg12 proteins, respectively. Equal loading of proteins is illustrated by GAPDH bands. (**C**, **D**, **F**) The graphs show the relative expression of Atg5, Atg12 and Atg5-Atg12 proteins in the four groups, respectively. Values are presented as means ± S.E.M.; n = 5–7. *P < 0.05 versus WT saline group. #*P* < 0.05 versus WT Dox group. Comparisons among groups were performed by one-way ANOVA followed by a Newman-Keuls post hoc test for multiple comparisons.

Cardiomyocyte autophagy is important for the maintenance of cardiac structure and function^18^. Prior studies have shown that doxorubicin induces cardiomyocyte autophagy^19,20^. In the present study, immunohistochemical staining demonstrated that myocardial expression of LC3 was increased in the WT doxorubicin group (Fig. 6A,B), indicative of increased autophagosome formation, and the increase was prevented in the Nox2 KO doxorubicin group (Fig. 6A,B). Western blot showed that the proteins of LC3 II and Beclin1, a key mediator of autophagy initiation, were increased in the WT doxorubicin group (Fig. 6C–F). Nox2 deficiency had no effect in the saline-treated group (Supplementary Fig. 3A–C), but prevented the increases in LC3 II and Beclin1 proteins in the doxorubicin-treated group (Fig. 6C–F). The autophagy-related proteins Atg5 and Atg12 play an important role in the autophagosome formation^18,30^. The conjugation of Atg12 and Atg5 is required for the elongation of autophagosome. We have further found that Atg5, Atg12 and Atg5-Atg12 conjugate proteins were increased in the WT doxorubicin group and the increases were prevented in the Nox2 KO doxorubicin group (Fig. 7A–F). These results suggest that Nox2 NADPH oxidase mediates induction of myocyte autophagy, contributing to doxorubicin-induced cardiac myocyte atrophy and failure.

To further determine the effect of doxorubicin on autophagic activity, we have performed an additional experiment using chloroquine, an inhibitor of autophagic degradation (Supplementary Methods). Mice were treated with saline, chloroquine at a dose of 10 mg/kg (i.p. once daily for 7 days), doxorubicin at a single dose of 15 mg/kg (i.p.) and doxorubicin at a single dose of 15 mg/kg plus 10 mg/kg of chloroquine (beginning 1 h before doxorubicin injection, once daily for 7 days). Western blot analysis showed that LC3 II protein had no significant changes in the chloroquine-treated mice compared with the saline-treated mice (Supplementary Fig. 4A,B). LC3 II protein was markedly increased in the doxorubicin-treated mice, and the increase was prevented by the treatment of chloroquine (Supplementary Fig. 4A,B). The results suggest that chloroquine inhibits the initiation of autophagy via an unknown mechanism, although it exerts an effect at the digestion step^31^.

### Nox2 deficiency attenuates myocyte apoptosis and myocardial fibrosis in doxorubicin-induced cardiac atrophy and failure

As expected, anti-apoptotic protein Bcl-2 was decreased, pro-apoptotic protein Bax was increased, the ratio of Bcl-2/Bax was reduced, and cleaved caspase 3 protein, a marker of apoptosis was increased in the WT doxorubicin group. These changes were prevented in the Nox2 doxorubicin group (Supplementary Fig. 5A–F). These findings suggest that Nox2 NADPH oxidase mediates myocyte apoptosis, contributing to doxorubicin-induced cardiac atrophy and failure. Myocardial fibrosis measured by Masson trichrome staining was increased in the WT doxorubicin group, Nox2 deficiency attenuated the increase in the doxorubicin-treated group (Supplementary Fig. 6A,B).

## Discussion

**Figure 8:**
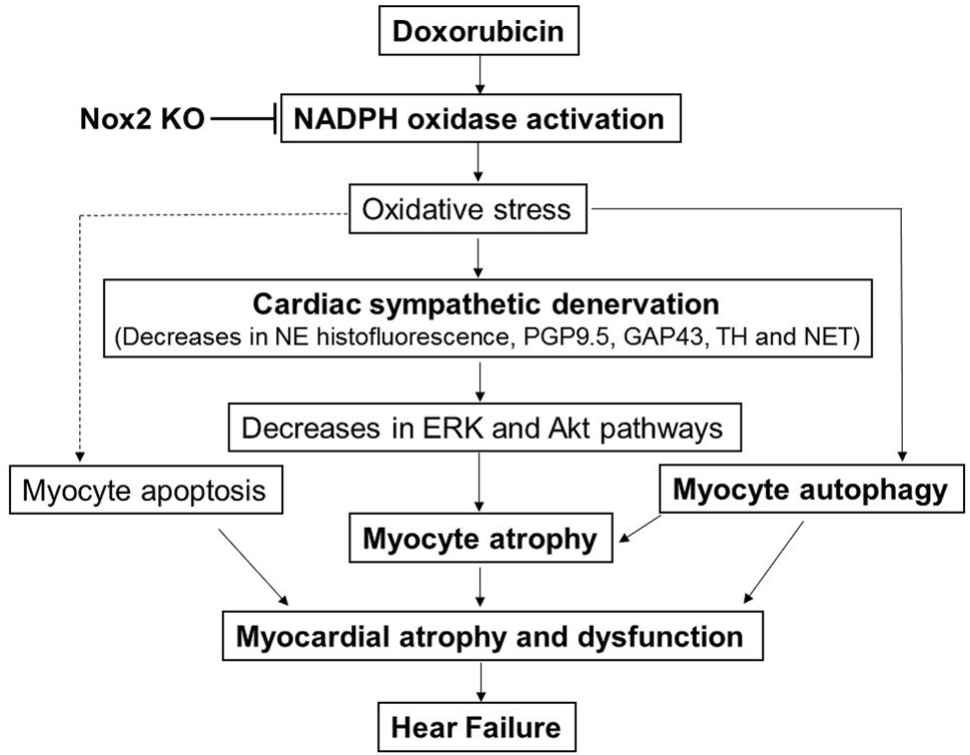
Scheme showing that doxorubicin induces NADPH oxidase-derived oxidative stress, resulting in cardiac sympathetic nerve terminal abnormalities as evidenced by the decreases in norepinephrine (NE) histofluorescence and PGP9.5, GAP43, tyrosine hydroxylase (TH) and noradrenaline transporter (NET) protein expression, myocyte autophagy activation and myocyte apoptosis—all of which contribute to cardiac atrophy and failure. NADPH oxidase 2 (Nox2) KO attenuates cardiac sympathetic nerve terminal abnormalities, prevents activation of myocyte autophagy and reduces myocyte apoptosis, thereby improving cardiac atrophy and failure in mice after doxorubicin treatment. These findings suggest that the inhibition of NADPH oxidase, the improvement of cardiac sympathetic nerve terminal innervation and/or the reduction of myocyte autophagy could have beneficial effects in doxorubicin cardiomyopathy and heart failure.

There are several major new findings in this study. First, Nox2 deficiency reduces myocardial oxidative stress, attenuates decreased catecholaminergic histofluorescence profiles, and restores protein expression levels of PGP9.5, GAP43, tyrosine hydroxylase and NET in doxorubicin-induced heart failure. Second, Nox2 deficiency attenuates the decreases in LV wall thickness, the heart weight to tibial length ratio and myocyte cross-sectional area and improves ERK and Akt/S6 signaling in doxorubicin-induced heart failure. Third, Nox2 deficiency prevents the increases in LC3 II, Beclin1 and Atg5-Atg12 protein expression and reduces myocyte apoptosis and myocardial fibrosis in doxorubicin-induced heart failure. Nox2 deficiency improves survival rate and attenuates the decrease in LV fractional shortening and the increase in the lung wet-to-dry weight ratio in doxorubicin-induced heart failure. These findings suggest that Nox2 NADPH oxidase-derived oxidative stress mediates cardiac sympathetic nerve terminal abnormalities, myocyte autophagy and myocyte apoptosis—all of which contribute to cardiac atrophy and failure after doxorubicin treatment Fig. 8.

We and others have shown a reduction of cardiac sympathetic innervation (reduced norepinephrine storage and loss of sympathetic nerve endings) and/or function (reduced norepinephrine reuptake) in congestive heart failure^5–7,26^. Cardiac sympathetic denervation assessed by reduced tyrosine hydroxylase expression has also been demonstrated in doxorubicin cardiomyopathy in patients^8,9^. Experimental studies have further confirmed cardiac sympathetic denervation in doxorubicin-induced cardiomyopathy in rats as evidenced by a reduction in the number of nerve fibers evaluated by PGP9.5 immunohistochemistry^10^. The release of norepinephrine from cardiac sympathetic nerve terminals was reduced in rabbits with chronic doxorubicin treatment^32^. Myocardial NET expression was downregulated in doxorubicin-induced heart failure in mice^33^. In the present study, we have shown that catecholaminergic histofluorescence profiles, PGP9.5 and GAP43, markers of sympathetic nerve terminals, the expression of myocardial tyrosine hydroxylase, a rate-limiting enzyme of noradrenaline synthesis, and the expression of myocardial NET were reduced in the WT mice with doxorubicin cardiomyopathy.

Numerous studies have shown that doxorubicin induces an increase in ROS in the heart and NADPH oxidase is a major source of ROS^12,13^. Our present study has shown that Nox2 deficiency attenuated the decreases in myocardial catecholaminergic histofluorescence profiles and PGP9.5, GAP43, tyrosine hydroxylase and NET protein expression in mice after doxorubicin treatment. These findings suggest that Nox2 mediates cardiac sympathetic nerve terminal abnormalities in doxorubicin-induced heart failure. These results are supported by our recent report demonstrating that NADPH oxidase inhibitors apocynin and diphenyleneiodonium improve cardiac sympathetic nerve terminal innervation in heart failure after myocardial infarction in rabbits^7^.

In skeletal muscle, denervated myofibers manifests atrophy^34^. Cardiac innervation regulates cardiomyocyte trophism^4^. Cardiac sympathetic denervation produced by pharmacological sympathectomy with hydroxyl-dopamine results in a significant reduction in the heart size^4^, suggesting that cardiac denervation causes atrophic remodeling. In the present study, we have shown that doxorubicin cardiomyopathy was characterized by reduced cardiac sympathetic nerve terminals, which were associated with the decreases in LV wall thickness, the ratio of heart weight to tibial length and myocyte cross-sectional area in mice. In consistence with our results, recent reports have demonstrated that doxorubicin induces cardiac atrophy in mice^35,36^. Doxorubicin causes a decrease in cardiac mass in mice and human^3^. Patients with anthracycline therapy have a significant reduction in LV mass, suggesting that LV atrophy is mediated by a reduction in cardiomyocyte size^37,38^. Our present study has further demonstrated that doxorubicin-induced cardiac atrophy coincides with LV systolic dysfunction. Similarly, others have also shown that doxorubicin causes a decrease in LV systolic function that parallels cardiac atrophy^3^. These results indicate that doxorubicin-induced cardiac sympathetic nerve terminal abnormalities cause cardiomyocyte atrophy, leading to LV systolic dysfunction.

Doxorubicin induces myocardial NADPH oxidase activation. Nox2 NADPH oxidase stimulates myocardial remodeling and dysfunction associated with doxorubicin treatment^12,13^. Studies have shown that the neurotransmitter norepinephrine released from cardiac sympathetic nerve terminals plays an important role in regulating cardiomyocyte trophism in vitro^4,39^. The loss of cardiac sympathetic neurotransmitters is a feature of heart failure^40^. Our present study has further demonstrated that Nox2 deficiency improves cardiac sympathetic innervation, leading to the amelioration of cardiac atrophy and function in doxorubicin cardiomyopathy. Taken together, these finding suggest that NADPH oxidase mediates cardiac sympathetic denervation that results in cardiomyocyte atrophy, contributing to cardiac atrophy and dysfunction in doxorubicin cardiomyopathy.

Extensive research has shown that PI3K/Akt signaling pathways are involved in regulating cardiac growth^41^. The reduction of Akt and ERK occurs in the denevated hearts^4^. PI3Kα pathway inhibition with doxorubicin promotes heart atrophy in mice^36^. Our present study has confirmed that the activities of ERK, Akt and S6 assessed by their respective phosphorylated protein expression were decreased in the denervated hearts following by the treatment of doxorubicin. Our present study has further shown that the decreases in the activities of ERK, Akt and S6 were prevented in the Nox2 KO hearts following by the doxorubicin treatment. These findings suggest that NADPH oxidase mediates the reduction of ERK, Akt and S6 activities, resulting in cardiomyocyte atrophy in doxorubicin cardiomyopathy.

Autophagy has been implicated in cardiac myocyte homeostasis^18,42^. Prior studies have demonstrated that myocyte autophagy is activated in the denervated heart which is characterized by cardiomyocyte atrophy^4^. Dysregulated myocyte autophagy occurs in doxorubicin-induced cardiomyopathy^43^. The autophagy gene Beclin1 deficiency inhibits doxorubicin-induced initiation of myocyte autophagy and protects against doxorubicin-induced cardiomyopathy^1^. In the present study, we have shown that Beclin1 protein, a key mediator of autophagy initiation, and LC3 II, Atg5, Atg12 and Atg5-Atg12 conjugate, essential for autophagosome formation, were increased in doxorubicin-induced cardiac atrophy and failure. These results are further supported by the recent reports showing that myocyte autophagy is upregulated after acute doxorubicin administration and inhibition of autophagy initiation protects against acute doxorubicin-induced cardiomyopathy^20^. We have further found that Nox2 deficiency attenuated myocyte autophagy in doxorubicin cardiomyopathy, suggesting that NADPH oxidase mediates myocyte autophagy, contributing to cardiac atrophy and failure after doxorubicin treatment. These results are in consistent with the previous reports demonstrating that excessive myocyte autophagy coexists with myocyte atrophy and increased oxidative stress in doxorubicin cardiomyopathy^3,12,44^.

The increase in LC3 II protein may result from blockade of a downstream autophagic degradation process^1,31^. To distinguish these possibilities, we treated mice with chloroquine, an inhibitor of autophagic degradation^45^. LC3 II protein would be expected to accumulate in hearts treated with chloroquine. However, we did not observe the significant accumulation. We found that myocardial LC3 II protein was markedly increased in the doxorubicin-treated mice, and the increase was prevented by the treatment of chloroquine. These results suggest that chloroquine inhibits the initiation of autophagy via an unknown mechanism, although it exerts an effect at the digestion step^31^. Similarly, other studies have also shown that the initial step in the formation of autophagosomes was inhibited by chloroquine or by bafilomycin A1^45^. The reason behind the discrepancy between our results and other reports^1,31^ is not known, but it might be related to species, animal background, the dosage, frequency and duration of treatment, and the stages of diseases. Further studies are warranted to better understand the effect of doxorubicin on autophagic activity with more a specific approach.

Extensive studies have demonstrated that myocyte apoptosis is increased in doxorubicin cardiomyopathy^46^. Increased myocyte apoptosis contributes to doxorubicin-induced cardiac atrophy and failure^11^. As expected, our present study has demonstrated that anti-apoptotic protein Bcl-2 was decreased, pro-apoptotic protein Bax was increased, and cleaved caspase 3 protein, reflective of caspase activity, a marker of apoptosis, was increased in doxorubicin cardiomyopathy. We have further shown that Nox2 deficiency reduced myocyte apoptosis in doxorubicin cardiomyopathy. We have also shown that Nox2 deficiency reduced myocardial fibrosis in doxorubicin cardiomyopathy. These results are consistent with the previous reports^12,13^. These findings suggest that NADPH oxidase mediates myocyte apoptosis, at least in part, contributing to doxorubicin-induced cardiac atrophy and failure.

In conclusion, our data demonstrated that Nox2 deficiency attenuates cardiac sympathetic nerve terminal abnormalities, myocyte atrophy, myocyte autophagy, myocyte apoptosis and myocardial fibrosis and ameliorates cardiac atrophy and dysfunction in doxorubicin cardiomyopathy. These findings suggest that NADPH oxidase mediates doxorubicin heart failure through both the reduction of cardiac sympathetic nerve terminals and activation of myocyte autophagy, leading to cardiac atrophy and dysfunction. The inhibition of NADPH oxidase, the improvement of cardiac sympathetic nerve terminal innervation and/or the reduction of myocyte autophagy could have beneficial effects in doxorubicin cardiomyopathy and heart failure.

## Materials and methods

### Animal model and experimental protocol

Nox2 knockout (KO) (B6.129S-*Cybb*^*tm1Din*^/J, Stock No. 002365) and wild-type (WT) control mice (C57BL/6J, Stock No. 000664) were purchased from the Jackson Laboratory (Bar Harbor, ME, USA). 8–10 weeks old Nox2 KO and WT mice were randomly assigned to receive a single intraperitoneal injection of doxorubicin (15 mg/kg, Cayman Chemical Company, Ann Arbor, MI, USA) or equivalent volume of saline. The dose of doxorubicin used in this study was based on the previous report^47^.

The study was approved by the Institutional Animal Care and Use Committee at Shanxi Medical University and conformed to the Guide for the Care and Use of Laboratory Animals published by the US National Institute of Health. We confirm that the study is reported in accordance with ARRIVE guidelines.

### Echocardiographic measurements

The two-dimensional and M-mode echocardiograms were performed 7 days after doxorubicin or saline injection to assess LV dimension and function using a FUJIFILM VisualSonics Vevo 3100 high-resolution imaging system (Toronto, Canada) equipped with a 22-55-MHz MS550D transducer, as we have described^48^. Briefly, mice were anesthetized with isoflurane by a facemask at a concentration of 2.5% for induction and then 1.5% for maintenance. The heart was imaged in the two-dimensional parasternal short-axis view, and an M-mode echocardiogram of the mid-ventricle was recorded at the level of papillary muscles. LV end-diastolic dimension (EDD) and end-systolic dimension (ESD) were measured on the M-mode tracings and averaged from more than 3 cardiac cycles. Data analysis was performed offline with the use of a customized version of Vevo 3100 Analytic software. LV fractional shortening (FS) was calculated as [(EDD − ESD) × 100]/EDD. Measurements and analysis were performed by two different investigators blinded to the experimental groups.

### Animal euthanasia, organ weight and histology

The mice were euthanized by 5% isoflurane (route: inhalation) at the end of study. Heart, lung and liver were removed and weighed. LV with septum was weighed and sliced. The mid cavity slice of LV sample was fixed in 10% buffered formalin for histology. The rest of LV samples was stored in liquid nitrogen for biochemical studies. LV tissue sections were stained with hematoxylin and eosin and examined under a light microscope. Myocyte size was assessed by myocyte cross-sectional area as we have described^48^. Myocardial interstitial fibrosis were assessed by Masson trichrome staining as we have described^49^.

### Measurements of myocardial oxidative stress

LV tissue sections were blocked with 10% horse serum in phosphate-buffered saline (PBS), incubated with 8-hydroxydeoxyguanosine (8-OHdG) antibody (MilliporeSigma, Burlington, MA) and then incubated with biotin-conjugated anti-goat IgG (Vector Laboratory, Burlingame, CA). The sections were then incubated with avidin and biotinylated horseradish peroxidase macromolecular complex (ABC, Vector Laboratory), and stained with 3-amino-9-ethylcarbazole (AEC) and hematoxylin (Vector Laboratory). The samples were examined under a light microscope (AX 10, Zeiss). The area and intensity of staining were blinded to score for quantification as we have described^50^.

### Glyoxylic acid-induced histofluorescence

Histofluorescence specific for catecholamines was performed using the sucrose-potassium phosphate-glyoxylic acid (SPG) condensation method^6,7,51^. Briefly, LV tissue block was rapidly frozen on dry ice and stored in liquid nitrogen. Blocks were mounted on a cryostat (− 20 °C) and then sectioned at a thickness of 12 µm. Sections were dipped in SPG, dried, heated under oil at 95 °C for 2.5 min and viewed under a fluorescence microscope (AX 10, Zeiss).

### Immunohistochemistry

The paraffin or frozen myocardial tissue sections were blocked with 10% horse or goat serum in PBS, incubated with goat polyclonal anti-PGP9.5 antibody (Sigma-Aldrich, St. Louis, MO), mouse monoclonal anti-GAP43 antibody (Santa Cruz Biotechnology, Dallas, TX), mouse monoclonal anti-tyrosine hydroxylase antibody (Sigma-Aldrich) or mouse monoclonal anti-LC3β antibody (Santa Cruz Biotechnology) and then incubated with biotin-conjugated anti-goat or anti-mouse IgG (Vector Laboratory, Burlingame, CA). The sections were then incubated with ABC reagents (Vector Laboratory), and stained with AEC (Vector Laboratory) and hematoxylin. The samples were examined under a light microscope (AX 10, Zeiss). The area and intensity of staining were blinded to score for quantification as we have described^7,52^. The scoring range was as follows: 0, no visible staining; 1, faint staining; 2, moderate staining; and 3, strong staining.

### Western blot

Protein extracts were resolved by electrophoresis in 10–12% sodium dodecyl-sulfate-polyacrylamide gel and transferred to polyvinylidene fluoride membranes as we have described^48,50^. The membranes were blocked in 5% nonfat dry milk in Tris-buffered saline containing 0.05% Tween 20 (TBST). The resulting blots were incubated overnight with mouse monoclonal anti-tyrosine hydroxylase antibody (Sigma-Aldrich, St. Louis, MO), mouse monoclonal anti-GAP43 antibody (Santa Cruz Biotechnology, Dallas, TX), goat polyclonal anti-PGP9.5 antibody (Sigma-Aldrich), mouse monoclonal anti-noradrenaline transporter (NET) antibody (Abcam, Cambridge, MA), mouse monoclonal anti-ERK antibody (Santa Cruz Biotechnology), mouse monoclonal anti-p-ERK antibody (Santa Cruz Biotechnology), rabbit polyclonal anti-Akt antibody (Cell Signaling Technology, Danvers, MA), rabbit anti-p-Akt antibody (Cell Signaling Technology), mouse monoclonal anti-rpS6 antibody (Santa Cruz Biotechnology), mouse monoclonal anti-p-rpS6 antibody (Santa Cruz Biotechnology), rabbit polyclonal anti-LC3 antibody (Cell Signaling Technology), rabbit monoclonal anti-LC3B antibody (Abcam), rabbit polyclonal anti-beclin1 antibody (MBL international, Woburn, MA), mouse monoclonal anti-ATG5 antibody (Santa Cruz Biotechnology) or mouse monoclonal anti-ATG12 antibody (Santa Cruz Biotechnology). Mouse monoclonal anti-GAPDH antibody (Abcam, Cambridge, MA) was used to confirm equal loading conditions. After the incubation of primary antibodies, blots were incubated with the secondary antibody goat anti-mouse IgG-HRP, goat anti-rabbit IgG-HRP or donkey anti-goat IgG-HRP. The membrane was washed with TBST and then the bands were visualized with ECL detection reagents (Thermo Scientific Pierce, Waltham, MA) and the Gel Imaging System (Bio-Rad, CA, USA) and quantified using Labwork4.6 image analysis software. The optical density of samples was normalized to a control sample in an arbitrary densitometry unit.

### Statistical analysis

The data were analyzed with GraphPad Prism 5 software (GraphPad Software, Inc. La Jolla, CA, USA). Results are presented as means ± standard error of the mean (S.E.M.). Comparisons among groups were performed by analysis of variance (ANOVA) followed by a Bonferroni post hoc test for multiple comparisons. Comparisons between two groups were used unpaired student’s test. Survival was calculated by Kaplan–Meier analysis. A value of *P* < 0.05 was considered statistically significant.

### Supplementary Information

Supplementary Information.

## Data Availability

The data supporting the findings of this study are available from the corresponding author upon reasonable request.
