# Supplementary material for: NADPH oxidase 2 mediates cardiac sympathetic denervation and myocyte autophagy, resulting in cardiac atrophy and dysfunction in doxorubicin-induced cardiomyopathy
Source: Sci Rep. 2024 Mar 23;14:6971. doi: 10.1038/s41598-024-57090-2 (PMC10960835; doi:10.1038/s41598-024-57090-2)
Supplement: Supplementary file 1 — Supplementary Information. [file 41598_2024_57090_MOESM1_ESM.pdf]

NADPH oxidase 2 mediates cardiac sympathetic denervation and myocyte autophagy, resulting in cardiac atrophy and dysfunction in doxorubicin-induced cardiomyopathy

Yuan Ma[1,2#], Hui-Ping Zhao[1,2#], Li-Guo Yang[1,2], Lu Li[2], Ai-Lin Wang[1,2], Xiao-Juan Zhang[1,2], Ke Wang[1,2], Bin Yang[1,2], Zong-Feng Zhu[1,2], Pei-Jun Zhang[3], Jia-Pu Wang[1,2], Rui-Fang Chi[1,2], Bao Li[1,2], Fu-Zhong Qin[1,2*], Zhi-Peng Wang[4]

[1]The Second Hospital of Shanxi Medical University, Taiyuan 030001, Shanxi; [2]Shanxi Medical University, Taiyuan 030001, Shanxi, PR China; [3]Shanxi Datong University School of Medicine, Datong 037009, Shanxi, P.R. China; [4]China Institute for Radiation Protection, Taiyuan 030006, Shanxi, PR China

# Contributed equally

*** Corresponding Author:**

Fu-Zhong Qin, MD, PhD, Professor

The Second Hospital of Shanxi Medical University

382 Wuyi Road, Taiyuan 030001, Shanxi, P. R. China

Tel: +86 15296616896

Email: sxcvh13@sina.com

Supplementary Fig. 1

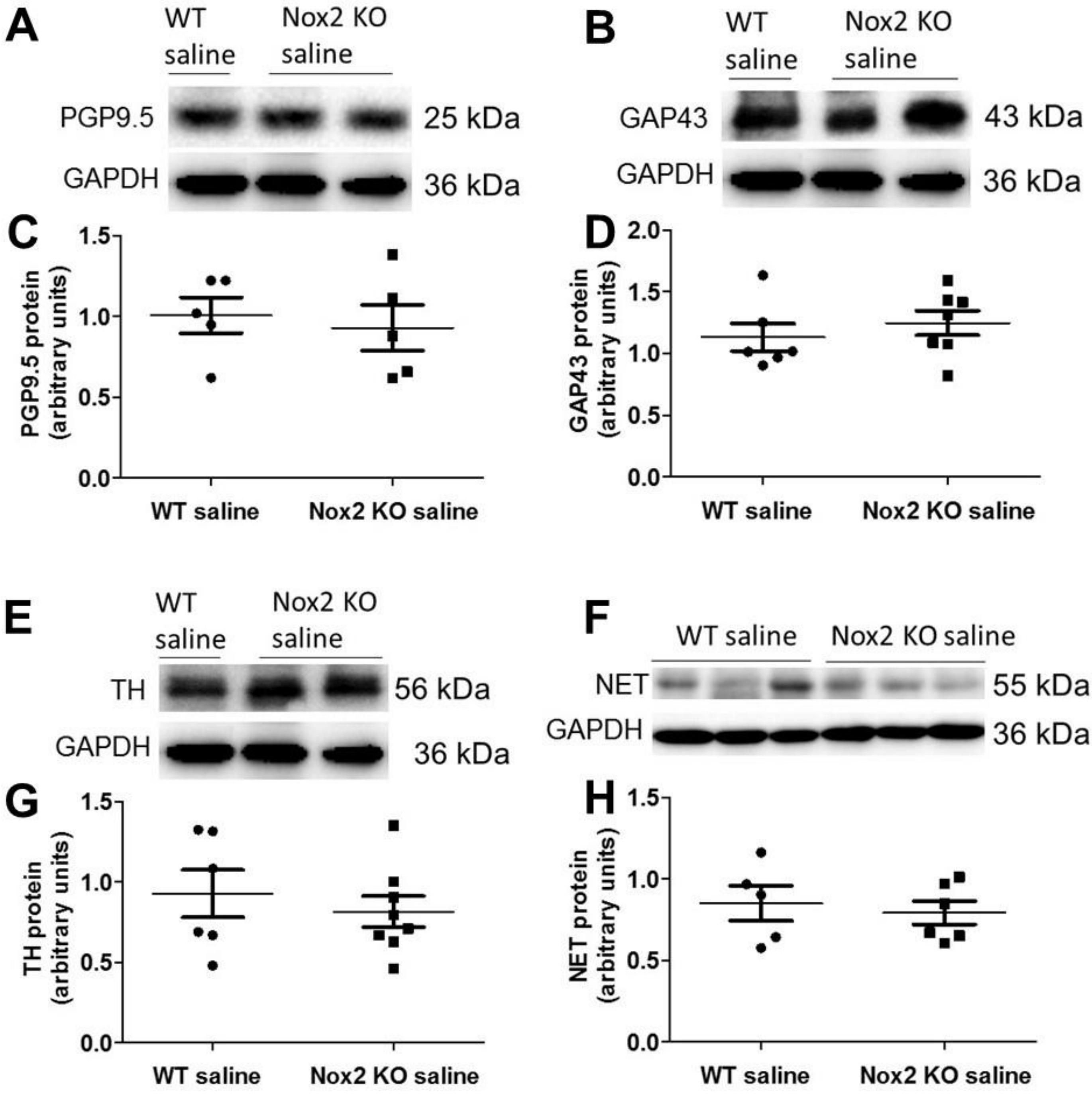

Supplementary Fig. 1. Changes in myocardial PGP9.5, GAP43, tyrosine hydroxylase (TH) and noradrenaline transporter (NET) proteins in WT saline and Nox2 KO saline groups. A, B, E, F. Representative Western blots of PGP9.5, GAP43, TH and NET proteins, respectively. Equal loading of proteins is illustrated by GAPDH bands. C, D, G, H. The graphs show the relative expression of PGP9.5, GAP43, TH and NET in the two groups, respectively. Values are presented as means ± S.E.M.; n = 5 for C; n = 6-7 for D; n = 6-8 for G; n = 5-6 for H. Comparisons between two groups were used unpaired Student's t-test.

Supplementary Fig. 2

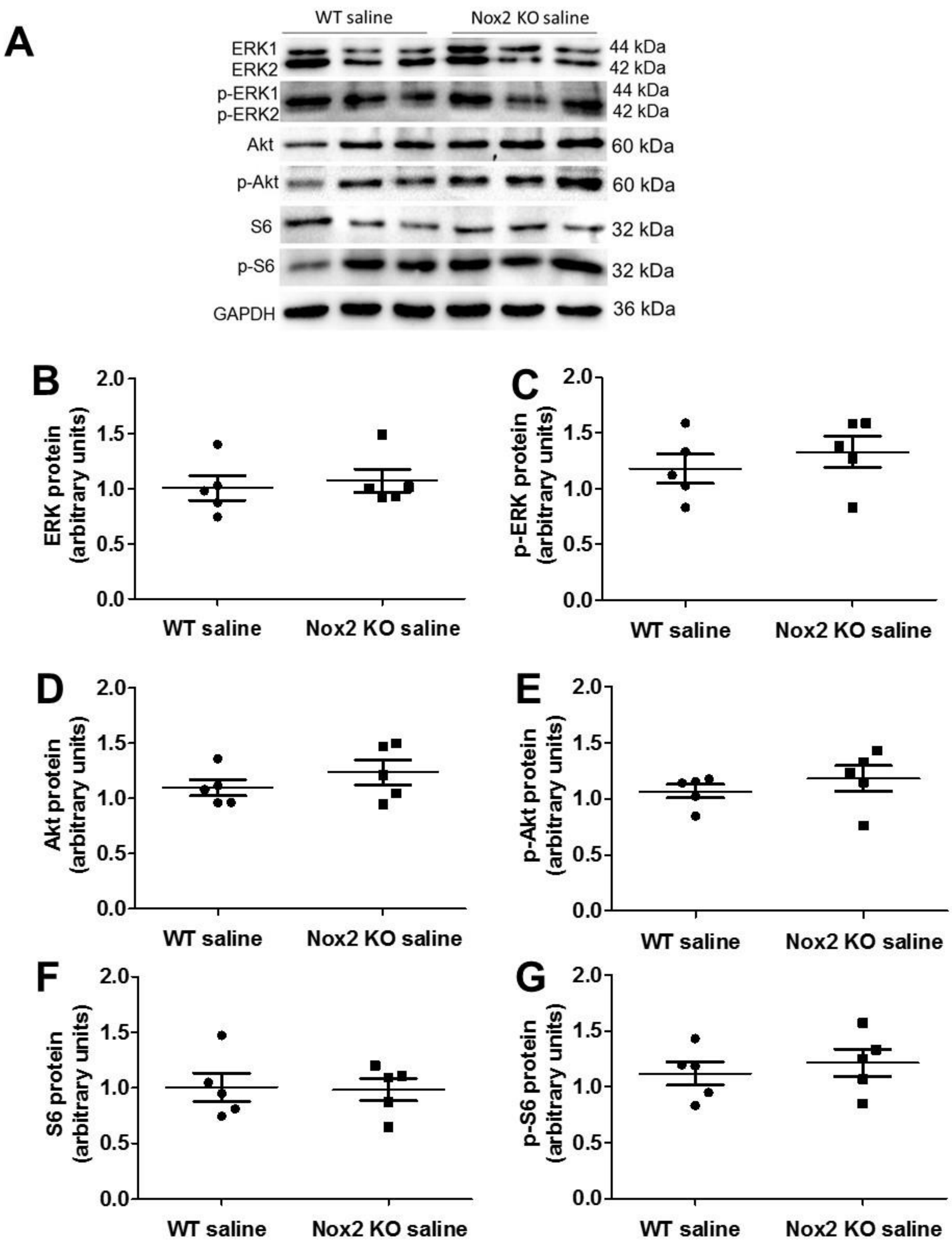

Supplementary Fig. 2. Changes in myocardial ERK, p-ERK, Akt, p-Akt, S6 and p-S6 proteins in WT saline and Nox2 KO saline groups. A. Representative Western blots of ERK, p-ERK, Akt, p-Akt, S6 and p-S6 proteins. Equal loading of proteins is illustrated by GAPDH bands. B-G. The graphs show the relative expression of ERK, p-ERK, Akt, p-Akt, S6 and p-S6 proteins in the two groups, respectively. Values are presented as means ± S.E.M.; n = 5. Comparisons between two groups were used unpaired Student's t-test.

Supplementary Fig. 3

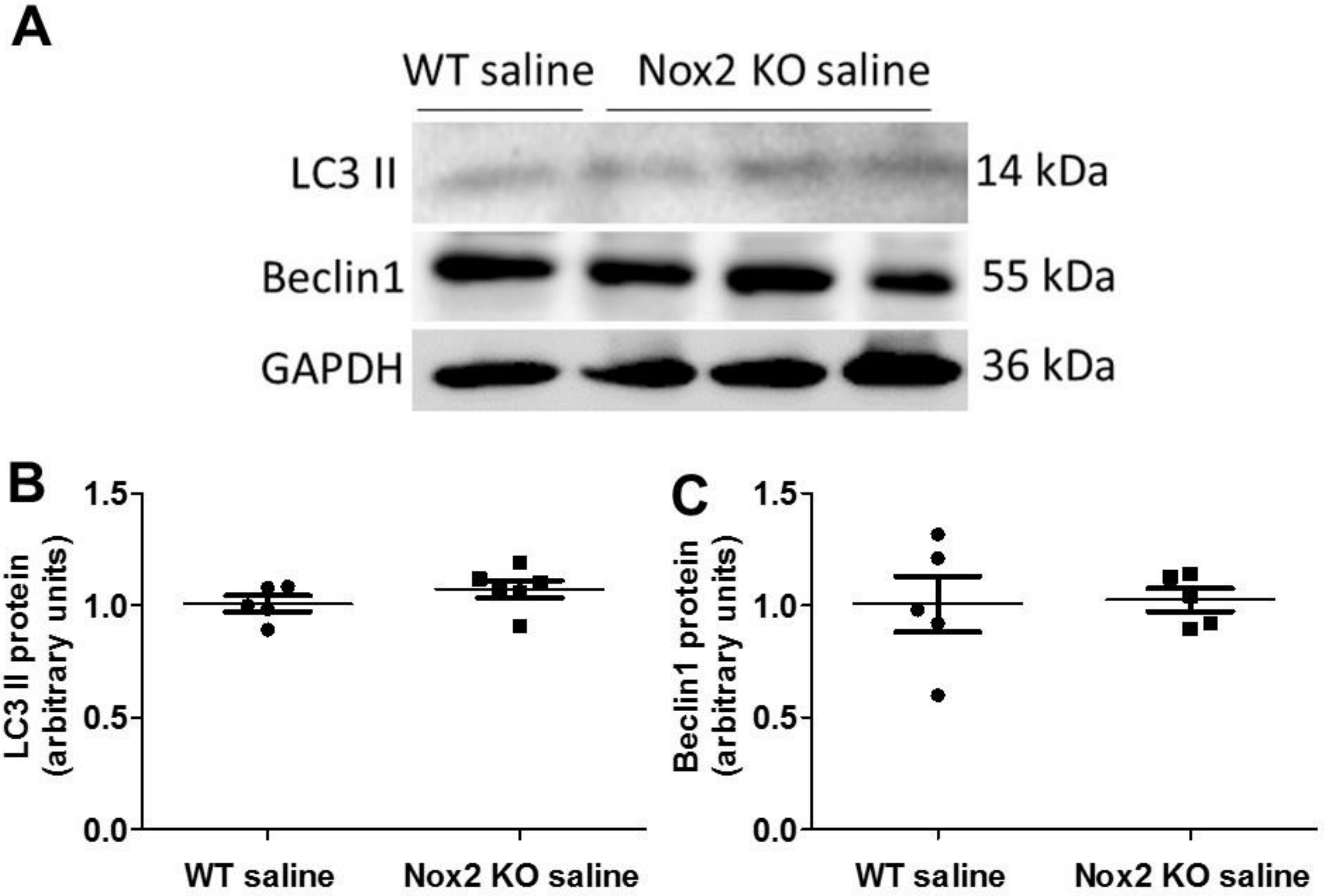

Supplementary Fig. 3. Changes in myocardial LC3 II and Beclin1 proteins in WT saline and Nox2 KO saline groups. A. Representative Western blots of LC3 II and Beclin1 proteins. Equal loading of proteins is illustrated by GAPDH bands. B, C. The graphs show the relative expression of LC3 II and Beclin1 proteins in the two groups, respectively. Values are presented as means ± S.E.M.; n = 5-6 for C, n = 5 for D. Comparisons between two groups were used unpaired Student's t-test.

Supplementary Methods

Eight-week old male C57BL/6 J mice were randomly divided into 4 experimental groups as follow: mice in control group (n=9) received normal saline (NS, equal volume, i.p.), mice in chloroquine group (n=8) received intraperitoneal injection of chloroquine (Sigma-Aldrich, St. Louis, MO) at a dose of 10 mg/kg (once a day for 7 days, i.p.), mice in doxorubicin group (n=10) received intraperitoneal injection of doxorubicin (Cayman Chemical, Ann Arbor, Michigan) at a single dose of 15 mg/kg, and mice in doxorubicin plus chloroquine group (n=8) received intraperitoneal injection of doxorubicin at a single dose of 15 mg/kg and 10 mg/kg of chloroquine (beginning 1 hour before doxorubicin injection, once a day for 7 days, i.p.). The doses of doxorubicin and chloroquine were based on our pilot studies and previous reports (Fu et al. Circ Res 2016 March 4;118(5):798-809; Kanamori et al. Am J Pathol 2013 March;182(3):701-13).

Supplementary Fig. 4

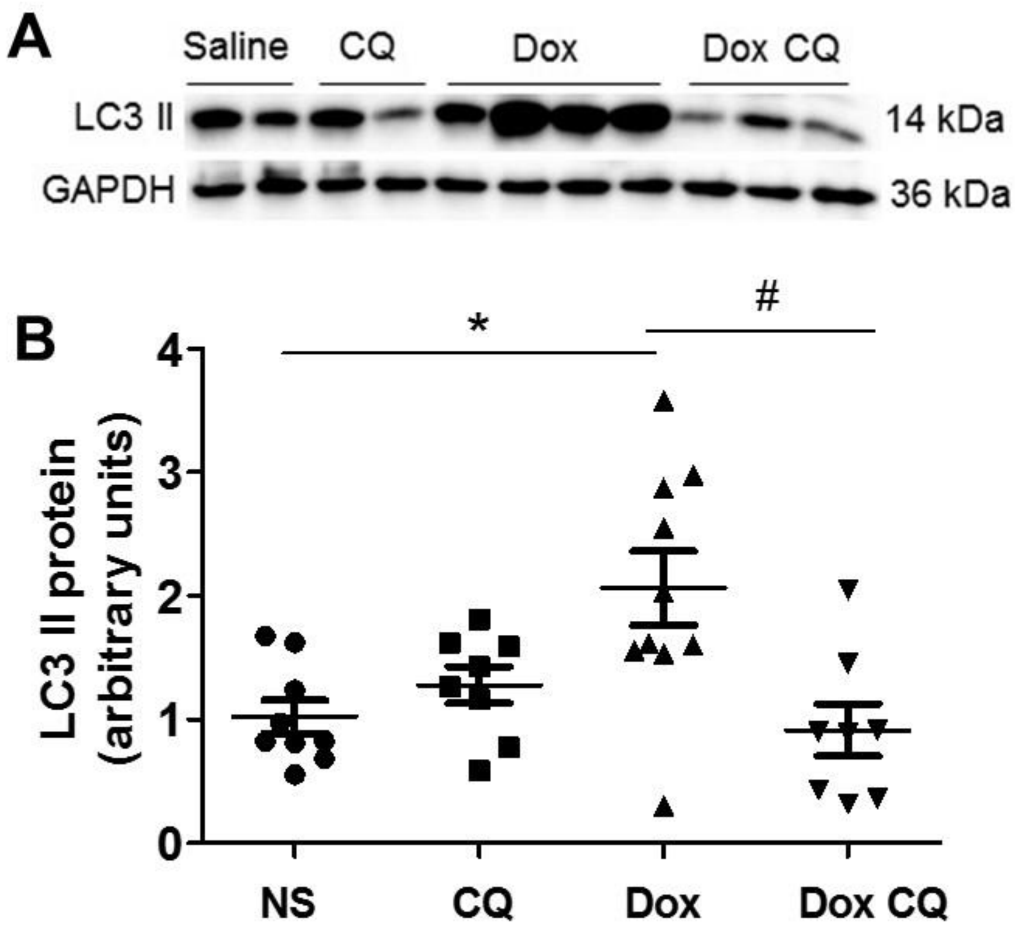

Supplementary Fig. 4. Changes in myocardial LC3 II protein in mice with saline (NS), chloroquine (CQ), doxorubicin (Dox) and Dox plus CQ treatments. A. Representative Western blot of LC3 II protein. Equal loading of proteins is illustrated by GAPDH bands. B. The graph shows the relative expression of LC3 II protein in the four groups, respectively. Values are presented as means ± S.E.M.; n = 8-10. *P < 0.05 vs. NS group. #P<0.05 vs. Dox group. Comparisons among groups were performed by one-way ANOVA followed by a Newman-Keuls test for multiple comparisons.

Supplementary Fig. 5

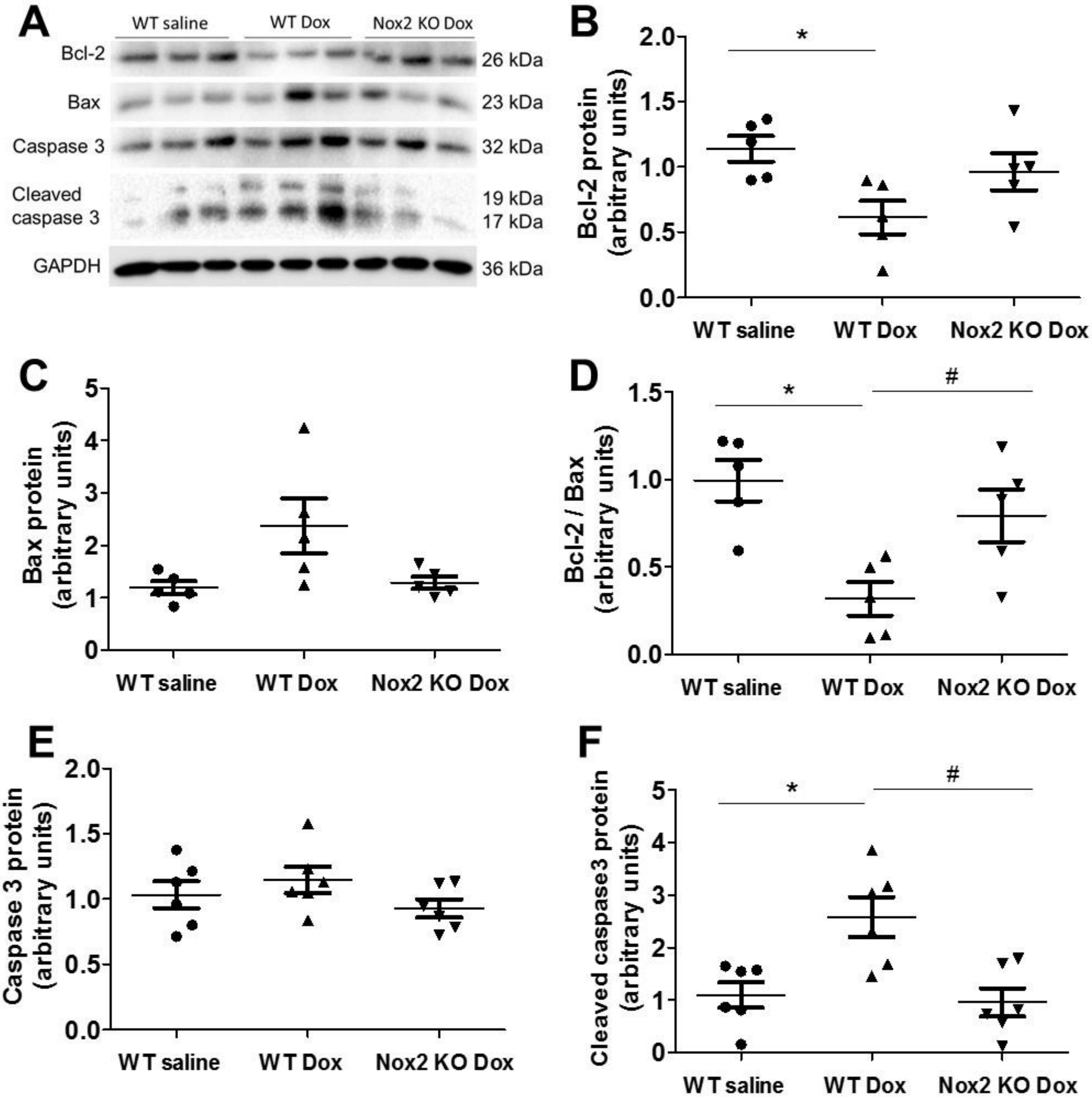

Supplementary Fig. 5. Changes in myocardial Bcl-2, Bax, caspase 3 and cleaved caspase 3 proteins in WT saline, WT doxorubicin (Dox) and Nox2 KO Dox groups. A. Representative Western blots of Bcl-2, Bax, caspase 3 and cleaved caspase 3 proteins. Equal loading of proteins is illustrated by GAPDH bands. B-F. The graphs show the relative expression of Bcl-2, Bax, caspase 3 and cleaved caspase 3 in the three groups, respectively. Values are presented as means ± S.E.M.; n = 5 for B, C, D; n = 6 for E, E. *P < 0.05 vs. WT saline group. #P<0.05 vs. WT Dox group. Comparisons among groups were performed by one-way ANOVA followed by a Bonferroni post hoc test for multiple comparisons.

Supplementary Fig. 6

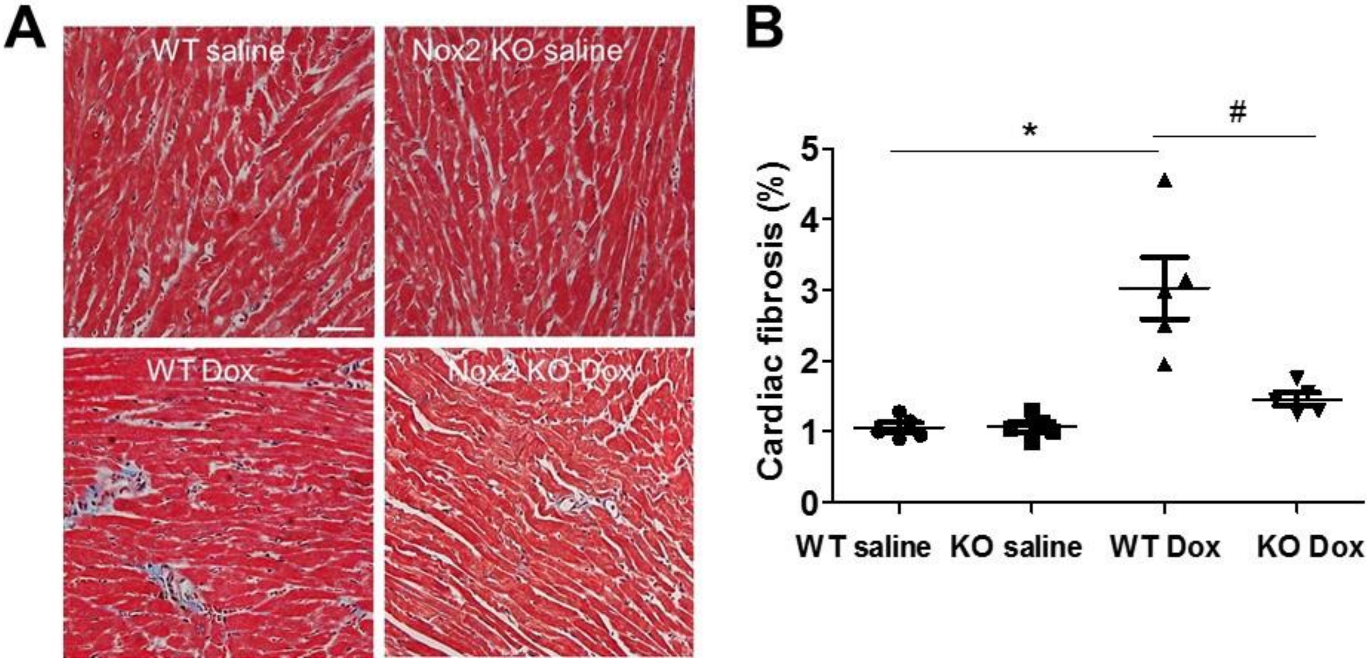

Supplementary Fig. 6. Changes in cardiac fibrosis in WT and Nox2 KO mice with saline or Doxorubicin (Dox) treatment. A. The representative photomicrographs of left ventricular myocardium with Masson trichrome staining. Bar = 50 μm. B. The graph shows the quantification of cardiac fibrosis as measured using ImageJ. Values are presented as means ± S.E.M.; n = 5. *P < 0.05 vs. WT saline group. #P<0.05 vs. WT Dox group. Comparisons among groups were performed by one-way ANOVA followed by a Bonferroni post hoc test for multiple comparisons.

Full-length blots for Figure 4 A

PGP9.5 (ab8189)

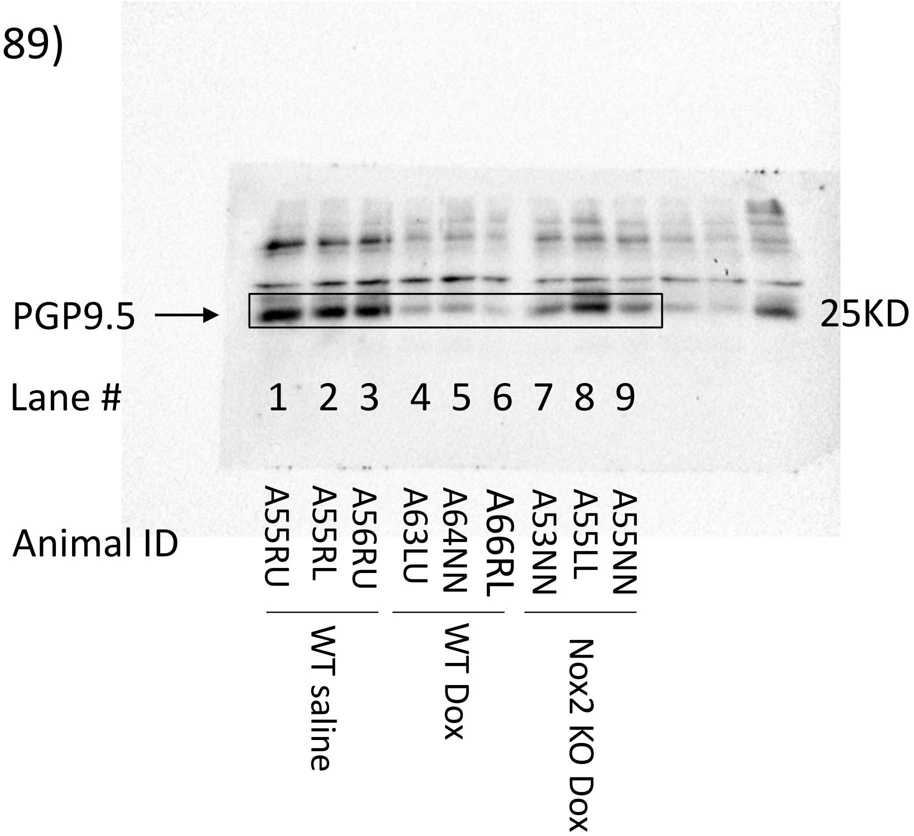

GAPDH (ab8245)

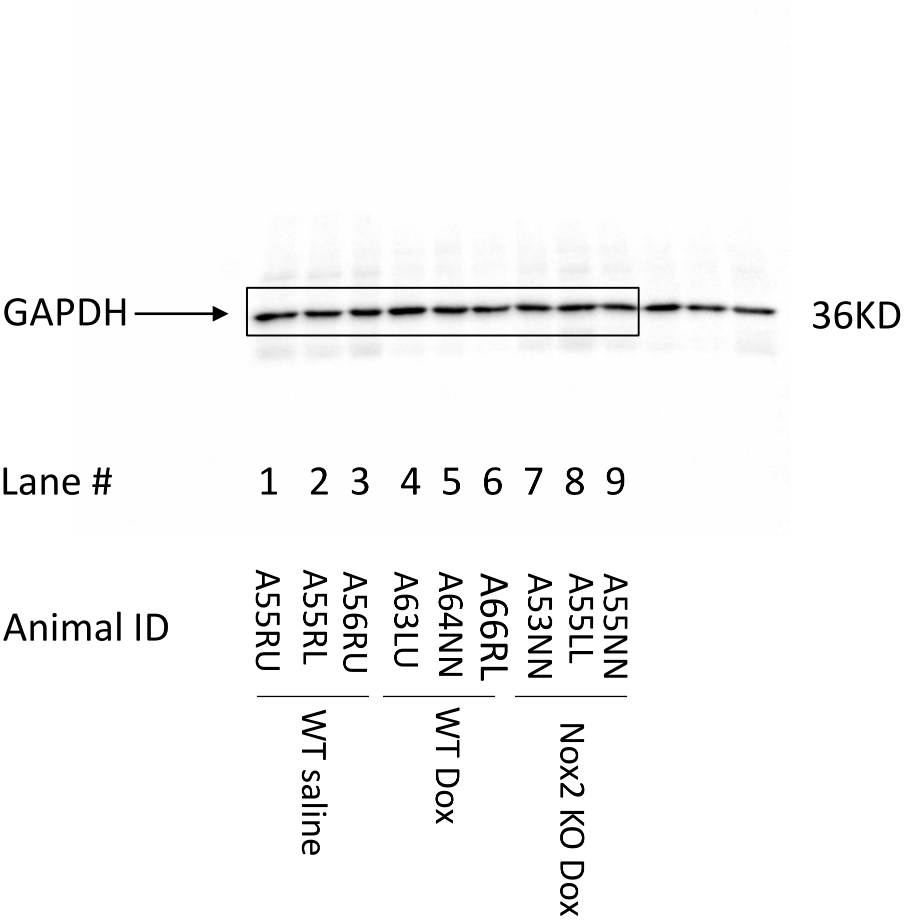

Full-length blots used for Figure 4 A.
Area shown in figures are indicated by boxes.

Full-length blots for Figure 4 B

GAP43 (sc33705)

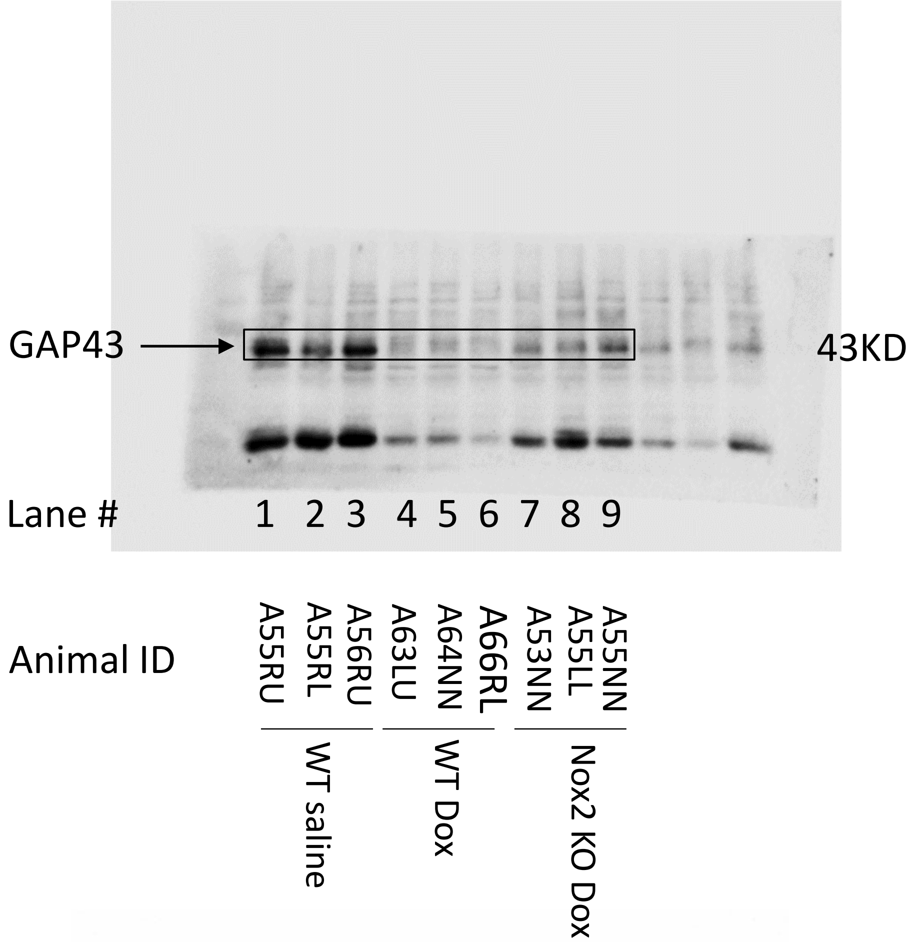

GAPDH (ab8245)

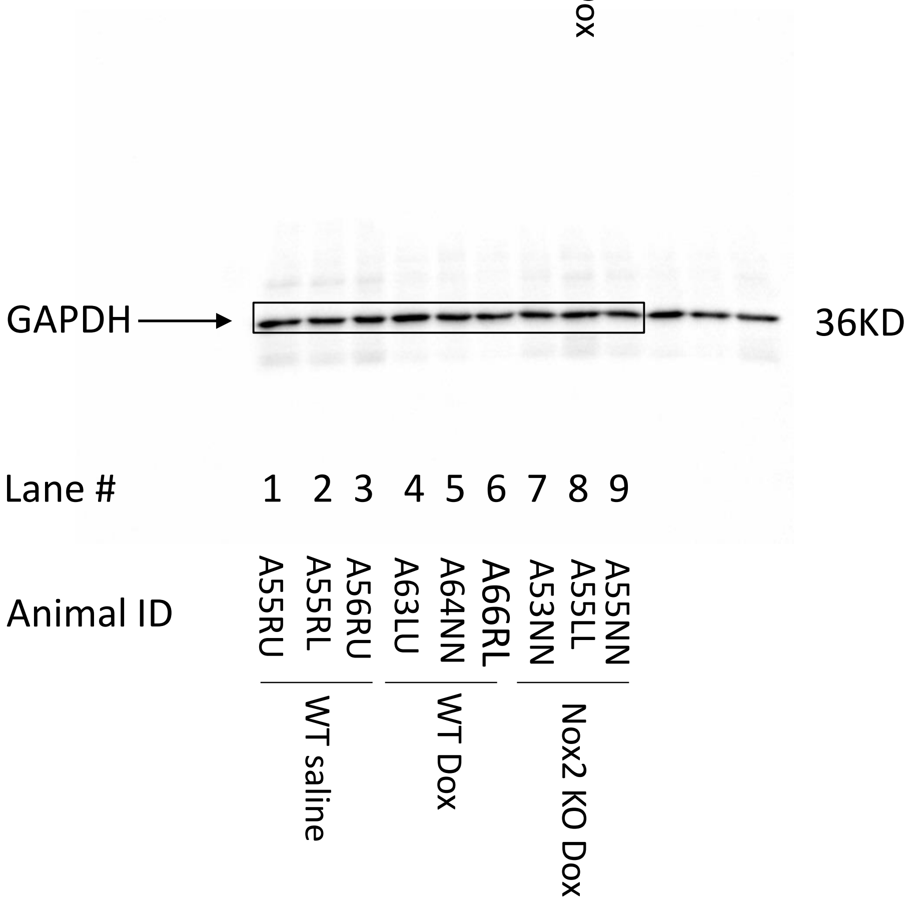

Full-length blots used for Figure 4 B.
Area shown in figures are indicated by boxes.

Full-length blots for Figure 4 E

Tyrosine hydroxylase (TH)

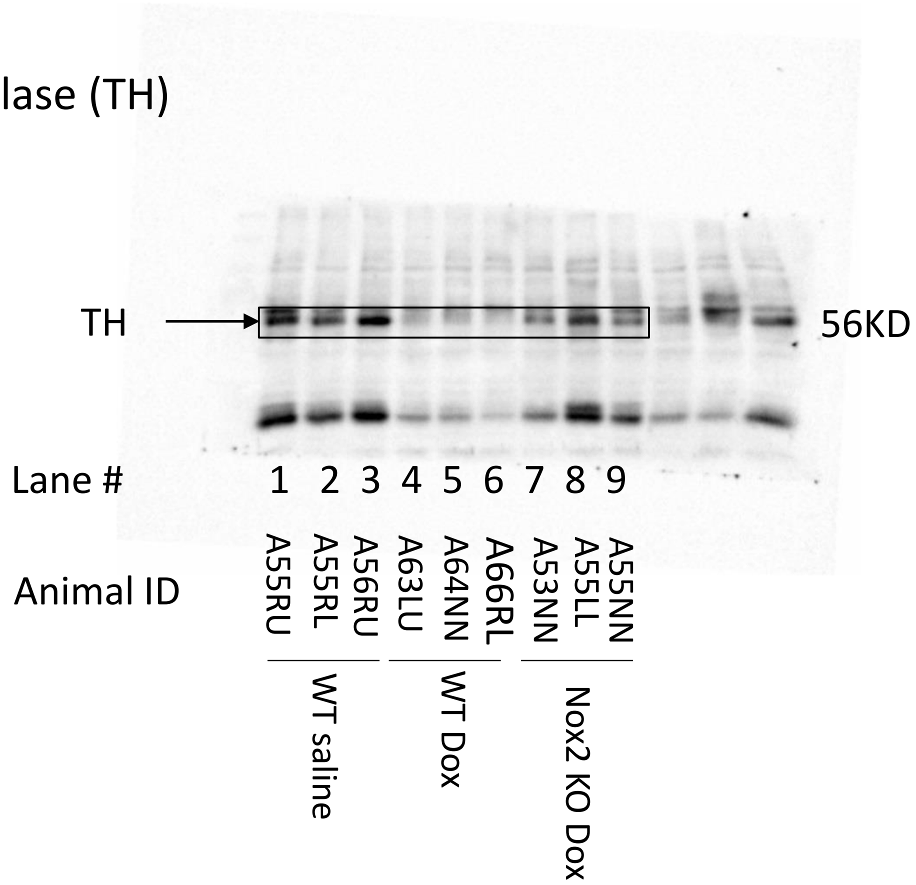

GAPDH (ab8245)

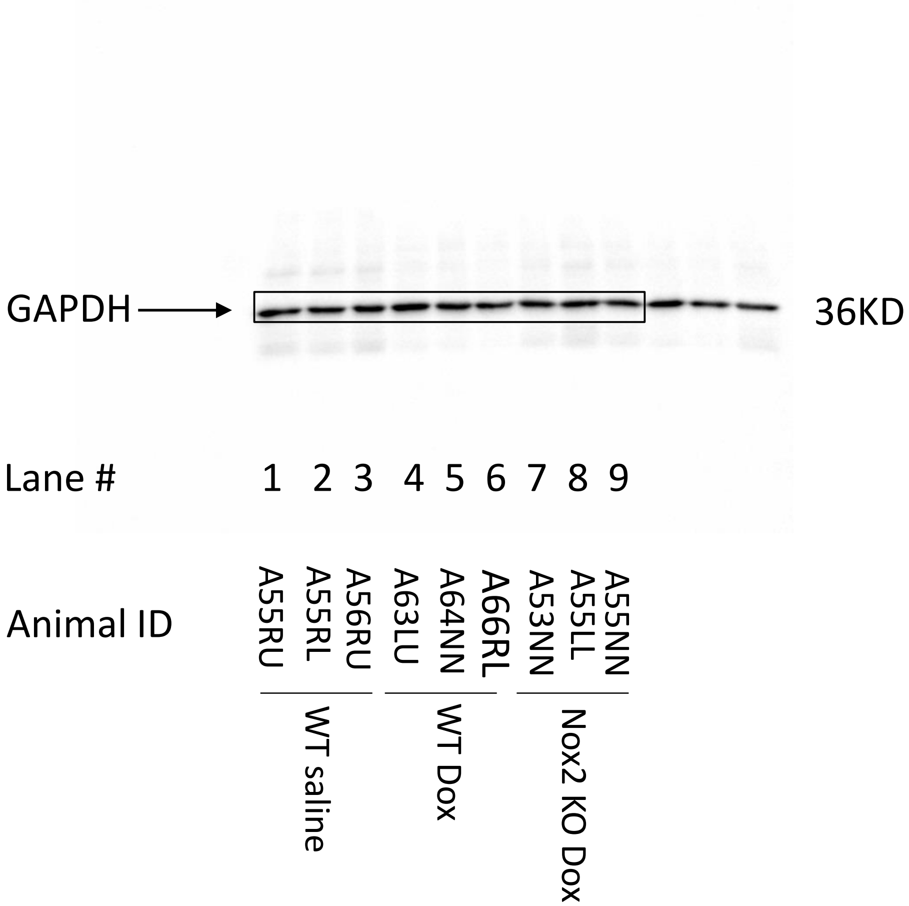

Full-length blots used for Figure 4 E.
Area shown in figures are indicated by boxes.

Full-length blots for Figure 4 F

Noradrenaline transporter (NET)

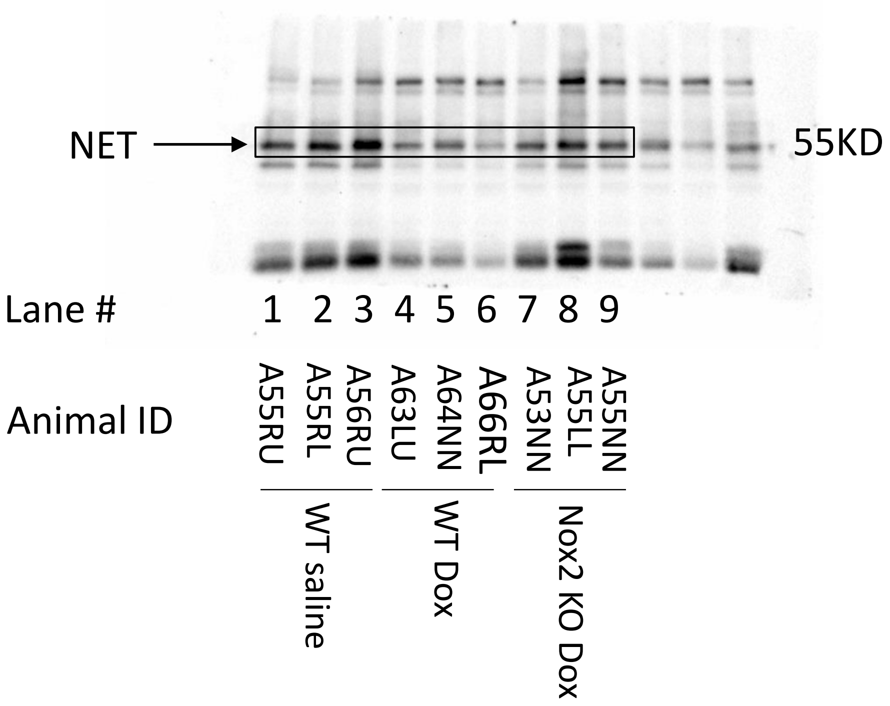

GAPDH (ab8245)

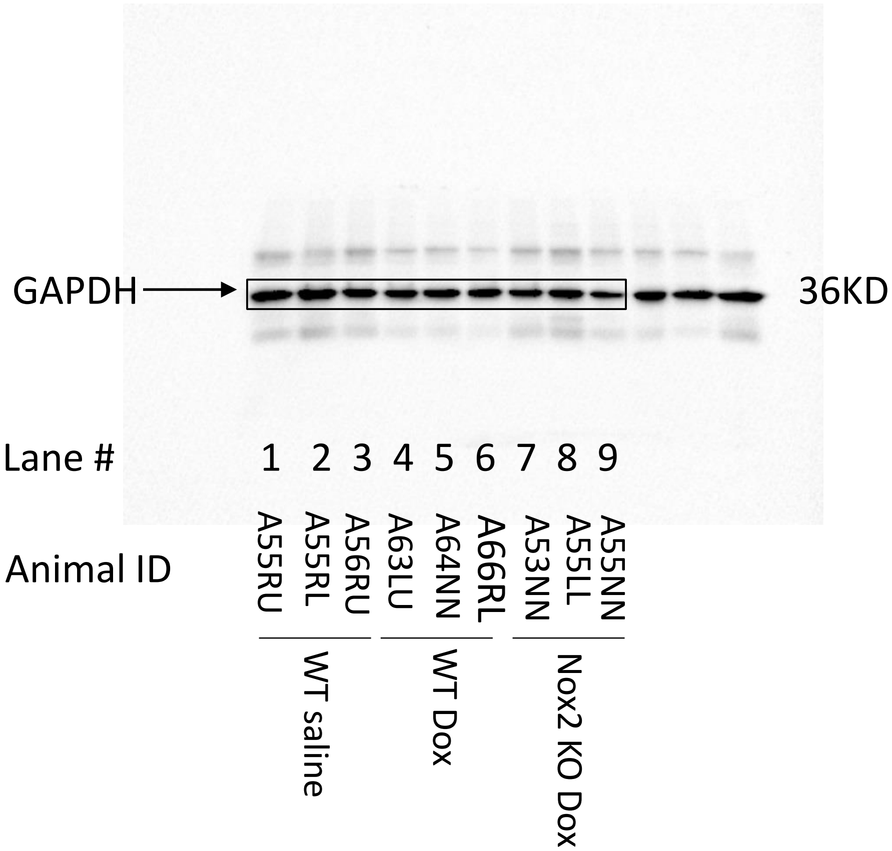

Full-length blots used for Figure 4 F.
Area shown in figures are indicated by boxes.

Full-length blots for Figure 5 A

ERK (sc1647)

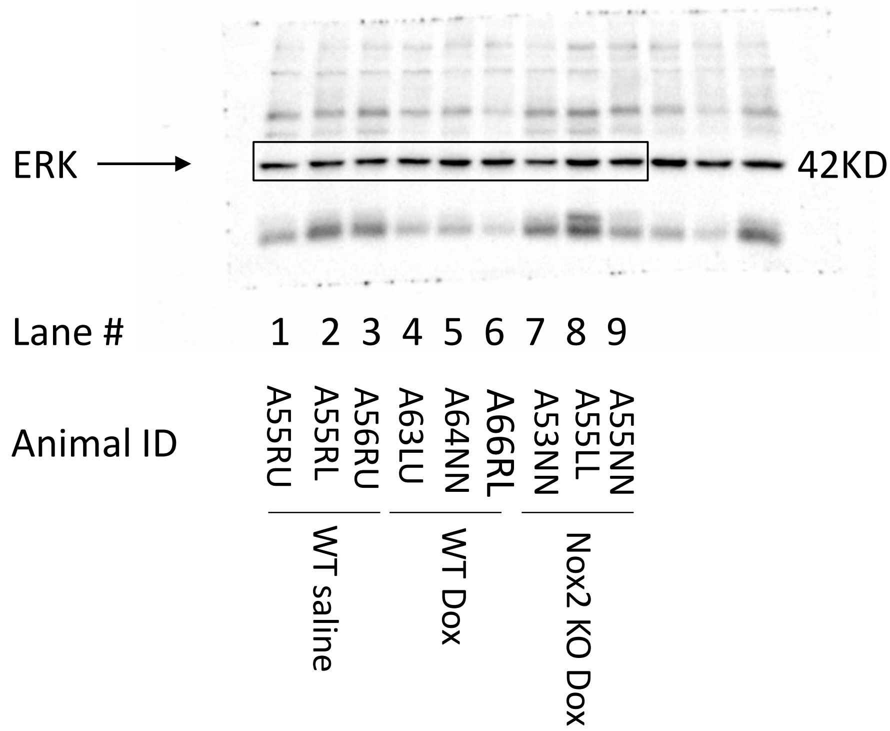

GAPDH (ab8245)

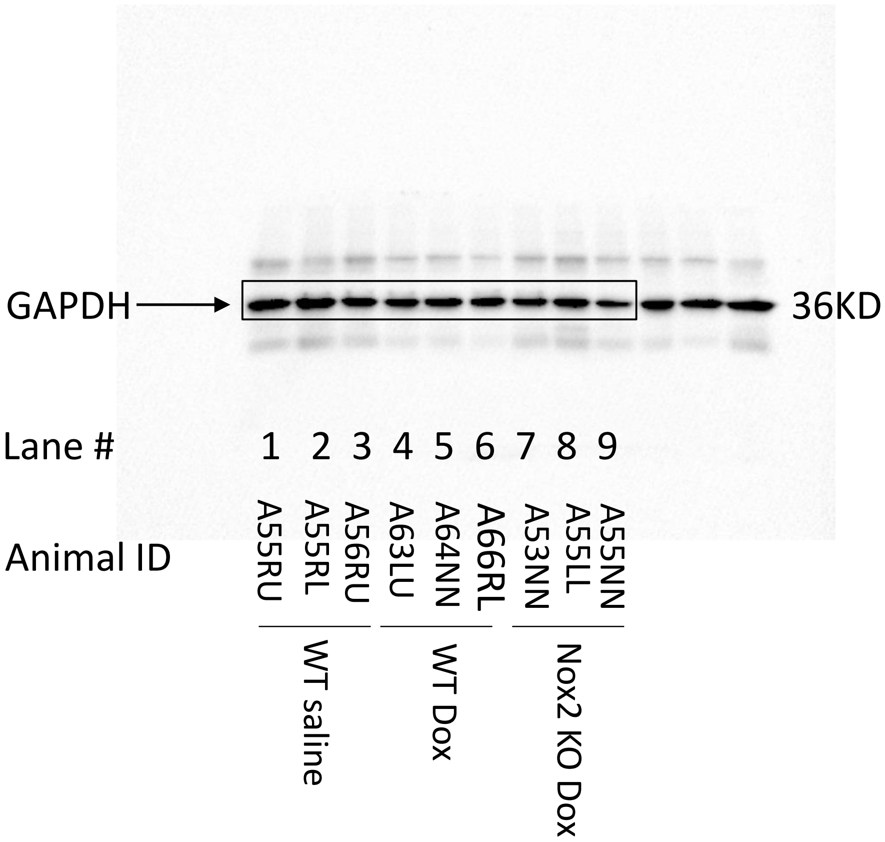

Full-length blots used for Figure 5A.
Area shown in figures are indicated by boxes.

Full-length blots for Figure 5 B

p-ERK (sc7383)

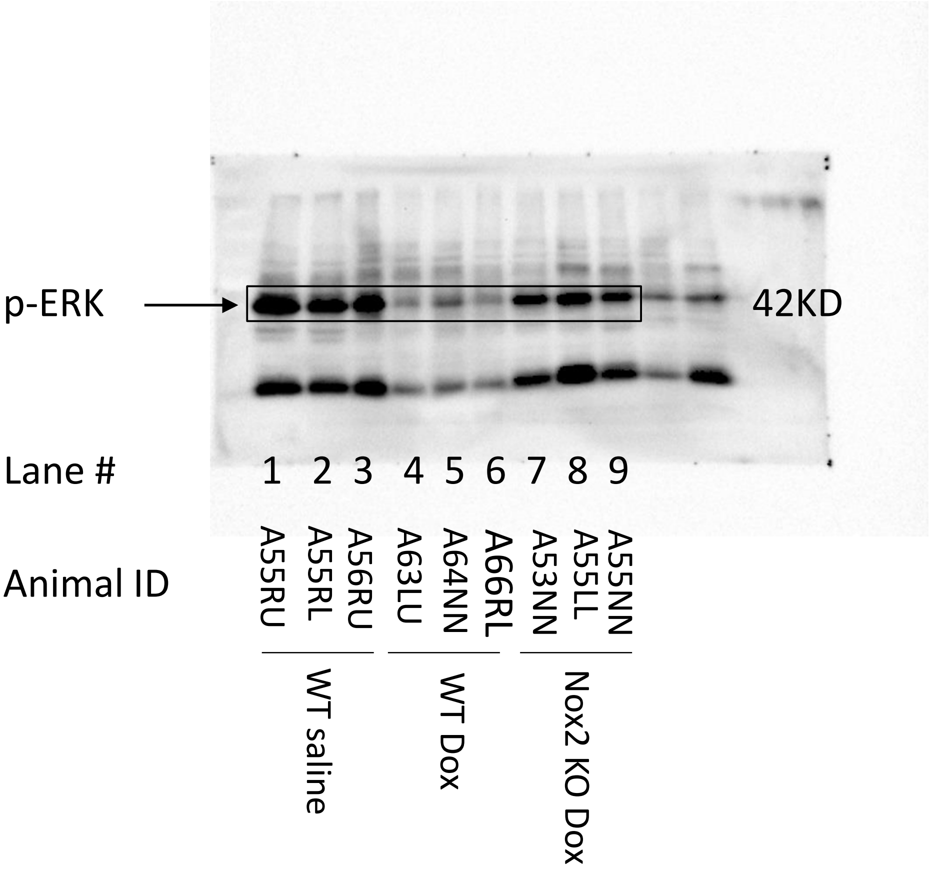

GAPDH (ab8245)

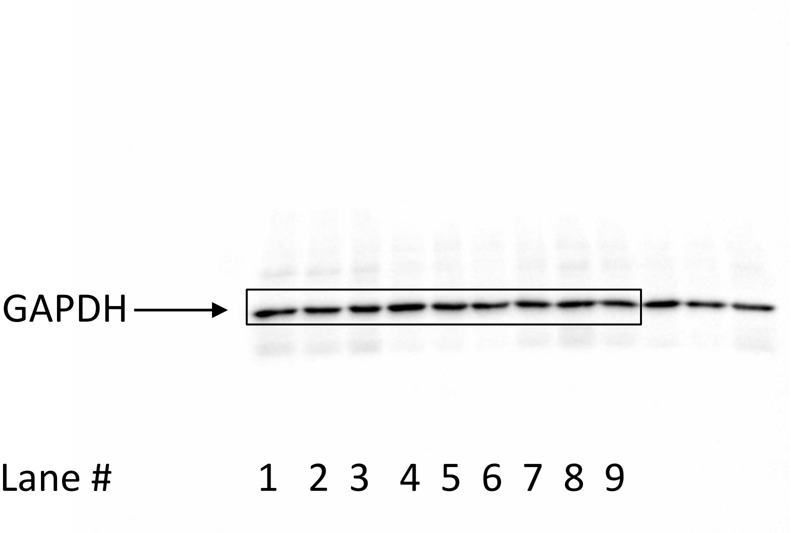

Full-length blots used for Figure 5B.
Area shown in figures are indicated by boxes.

Full-length blots for Figure 5 E

Akt (CST9272)

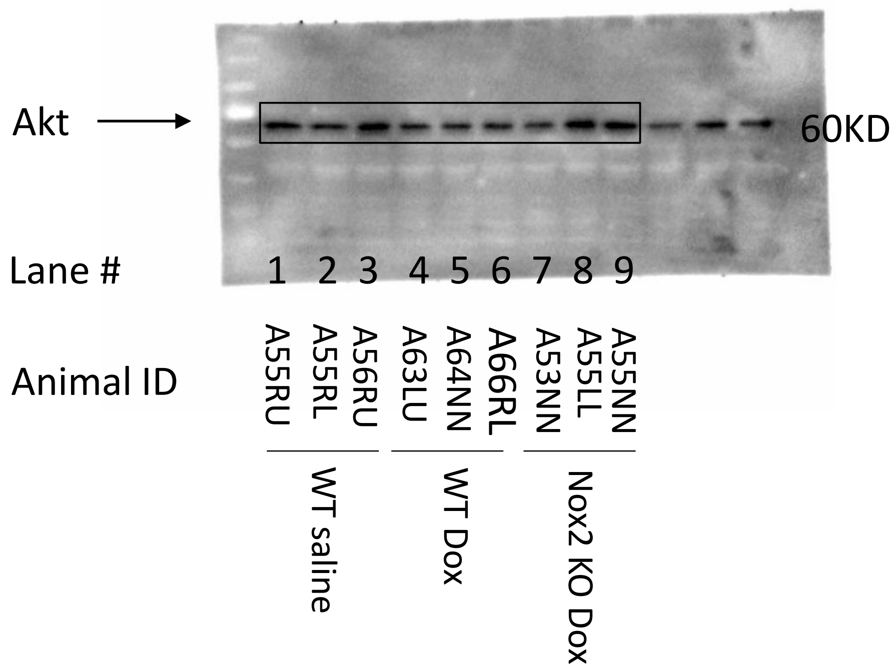

GAPDH (ab8245)

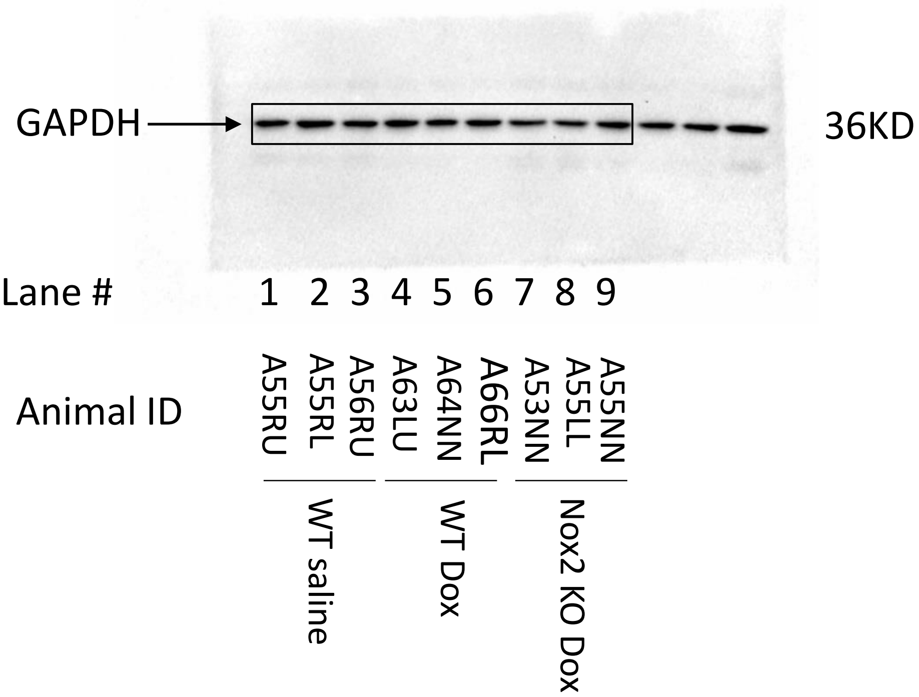

Full-length blots used for Figure 5E.
Area shown in figures are indicated by boxes.

Full-length blots for Figure 5 F

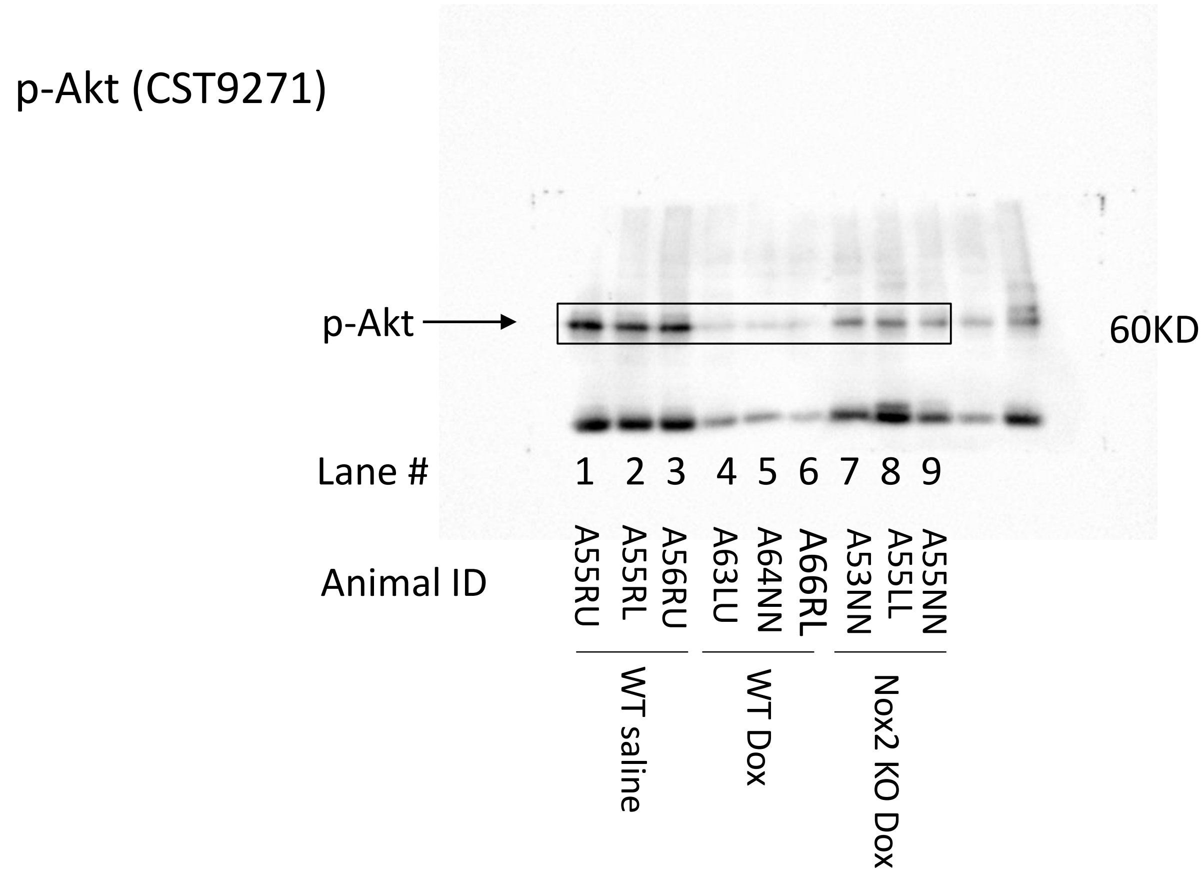

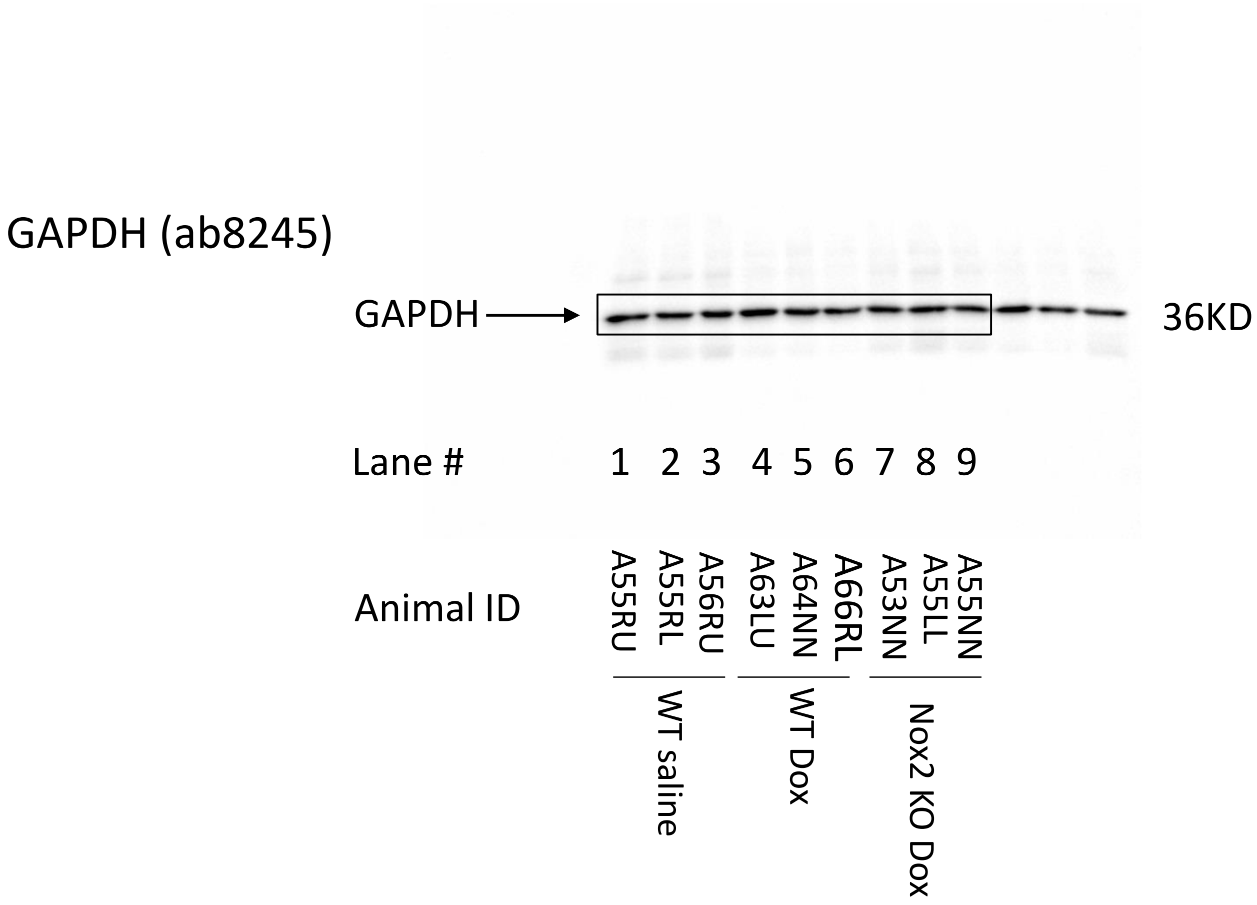

Full-length blots used for Figure 5F.
Area shown in figures are indicated by boxes.

Full-length blots for Figure 5 I

rpS6 (sc74459)

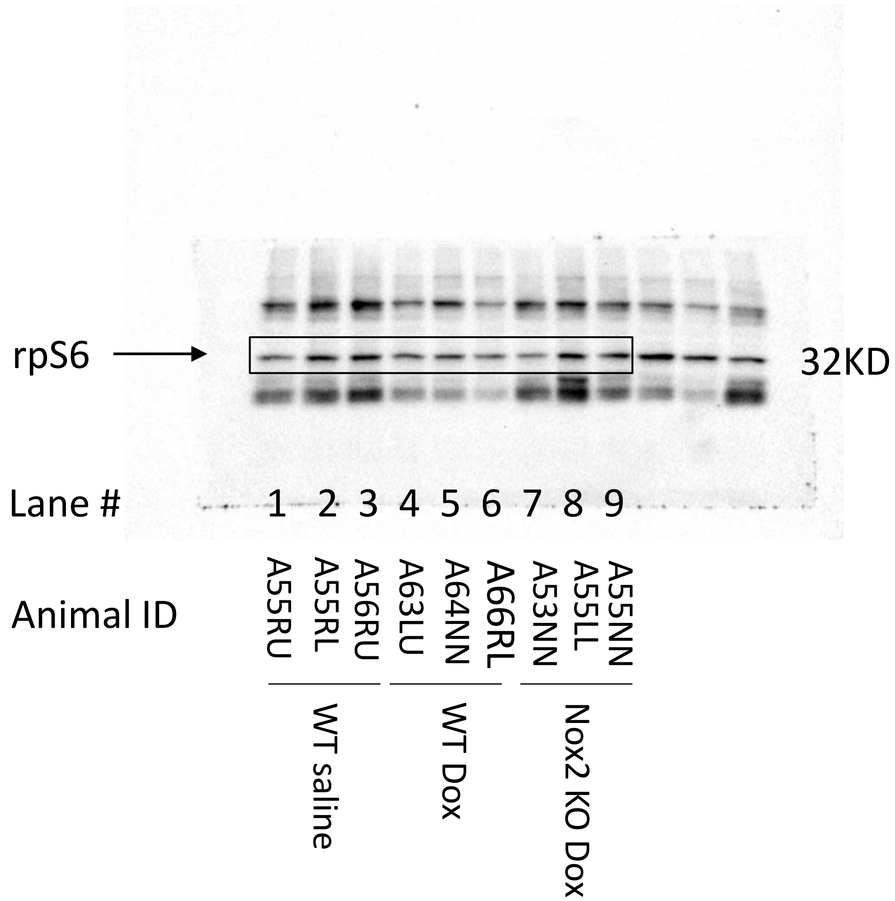

GAPDH (ab8245)

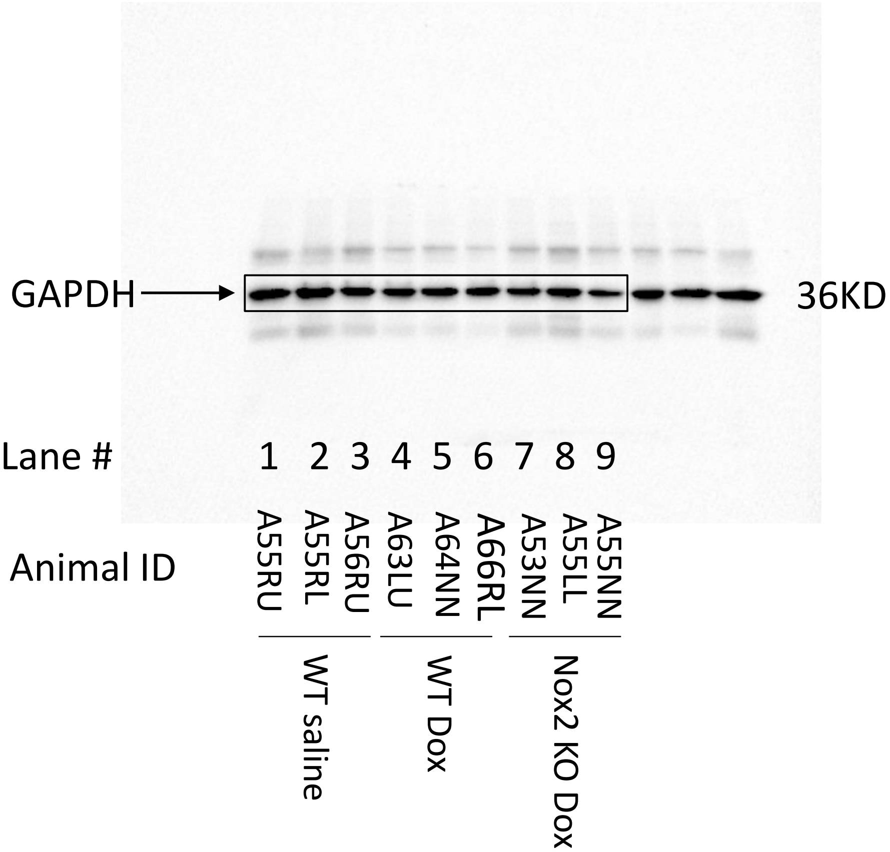

Full-length blots used for Figure 5I.
Area shown in figures are indicated by boxes.

Full-length blots for Figure 5 J

p-rpS6 (sc293144)

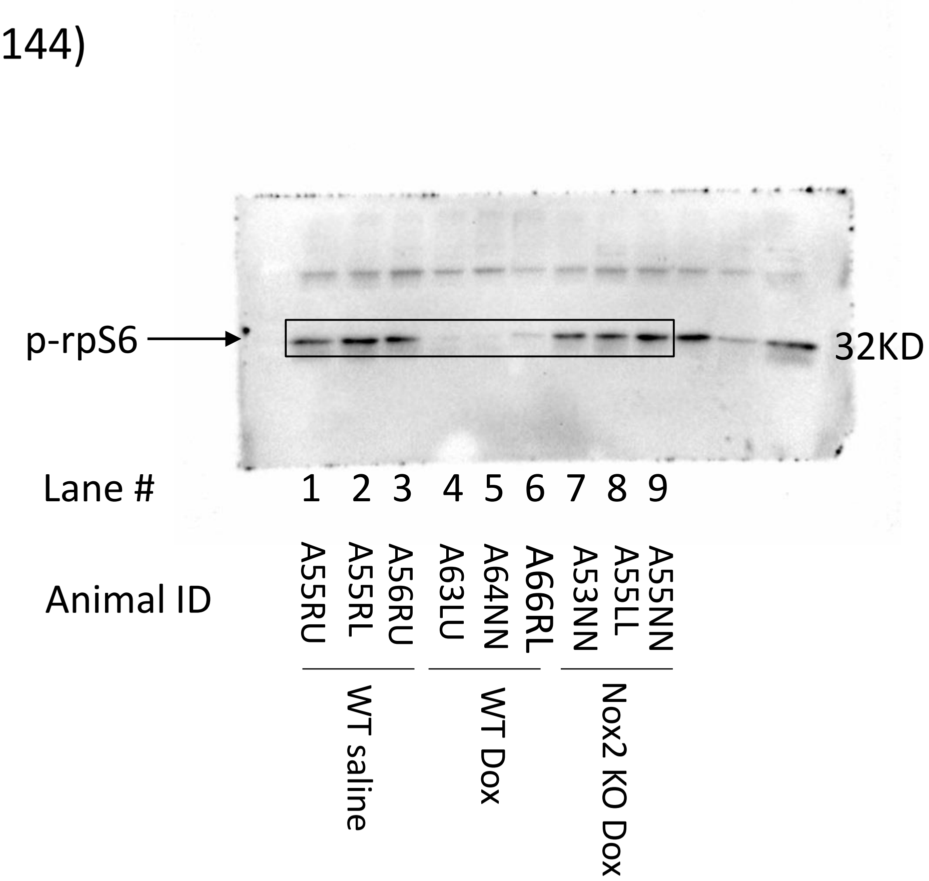

GAPDH (ab8245)

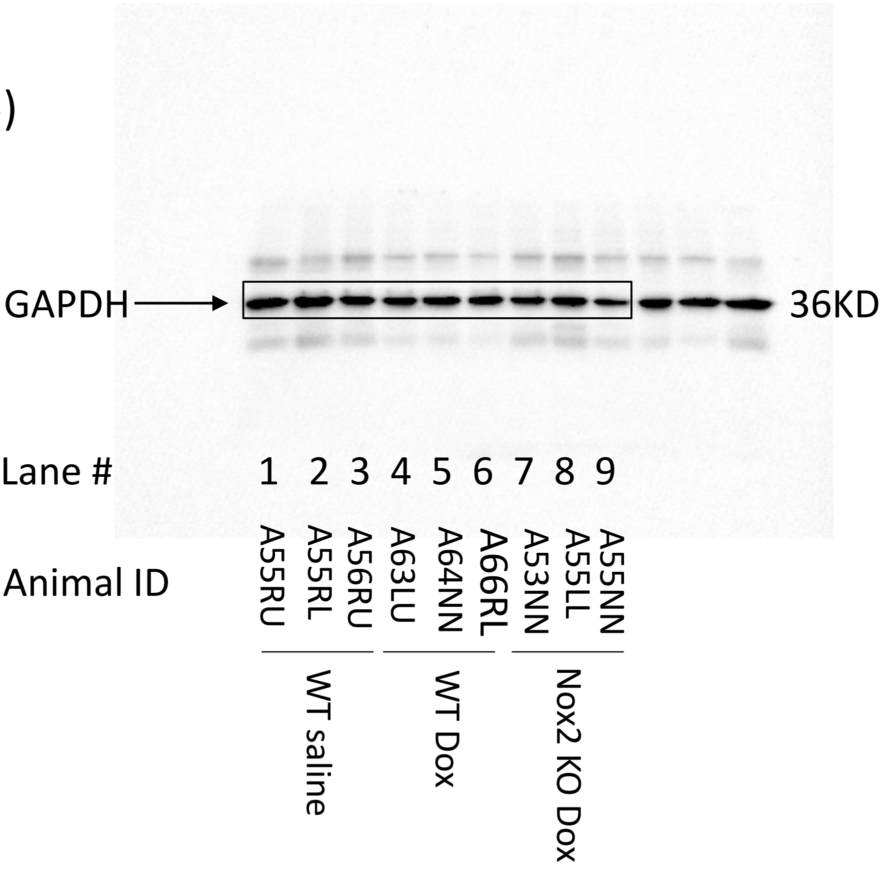

Full-length blots used for Figure 5J.
Area shown in figures are indicated by boxes.

Full-length blots for Figure 6 C

LC3A/B (CST4108)

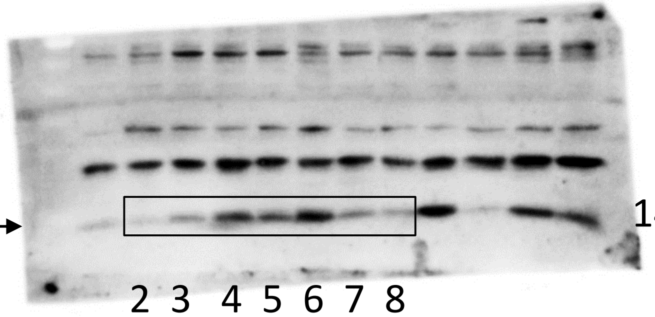

LC3 II → 14KD

Lane # 2 3 4 5 6 7 8

Animal ID: A55RL, A56RU, A63LU, A64NN, A66RL, A53NN, A55LL

WT saline | WT Dox | Nox2 KO Dox

GAPDH (ab8245)

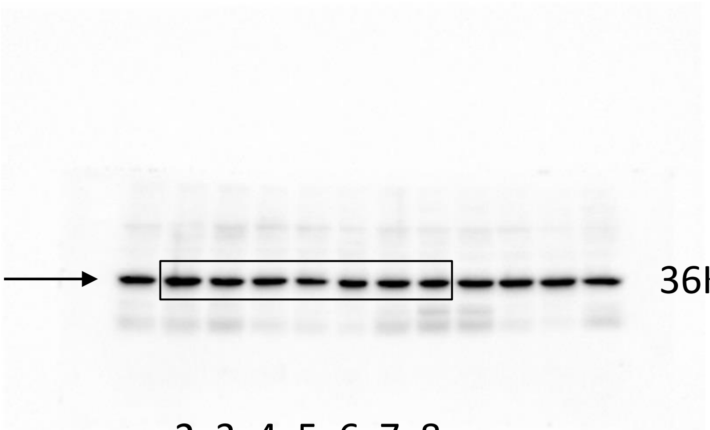

GAPDH → 36KD

Lane # 2 3 4 5 6 7 8

Animal ID: A55RL, A56RU, A63LU, A64NN, A66RL, A53NN, A55LL

WT saline | WT Dox | Nox2 KO Dox

Full-length blots used for Figure 6C.
Area shown in figures are indicated by boxes.

Full-length blots for Figure 6 D

Beclin1 (PD017)

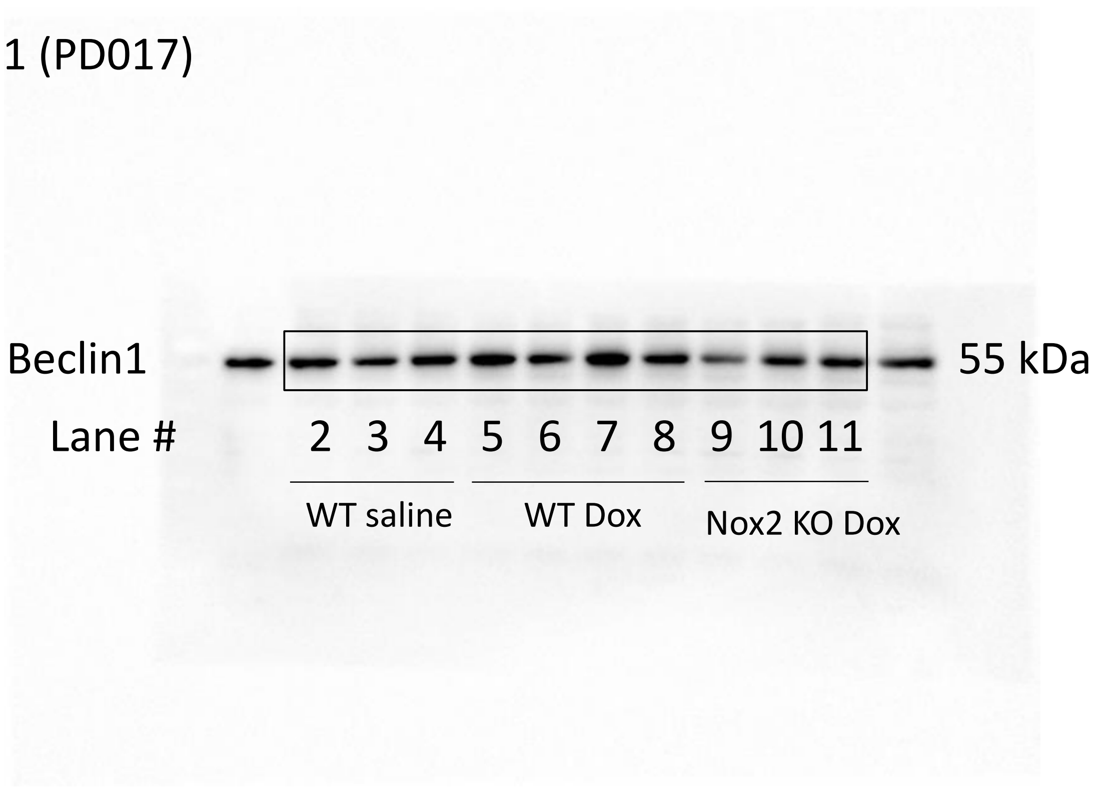

GAPDH (ab8245)

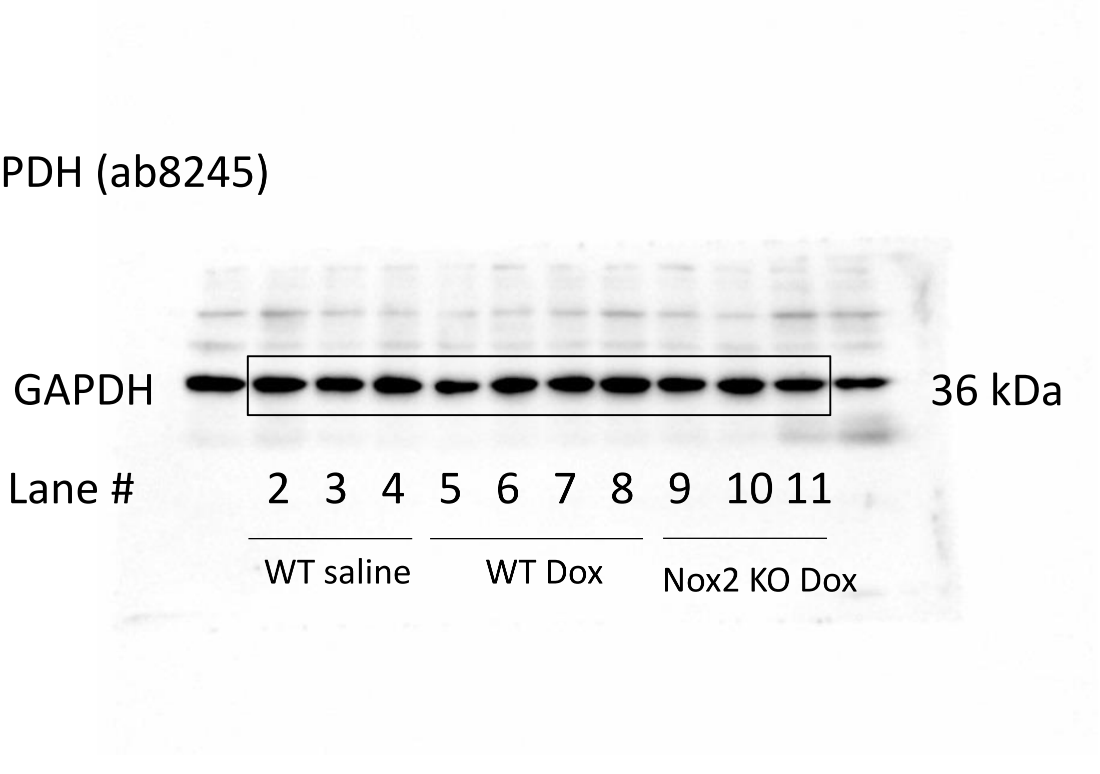

Full-length blots used for Figure 6D.
Area shown in figures are indicated by boxes.

Full-length blots for Figure 7 A and E

Atg5 (sc-133158)

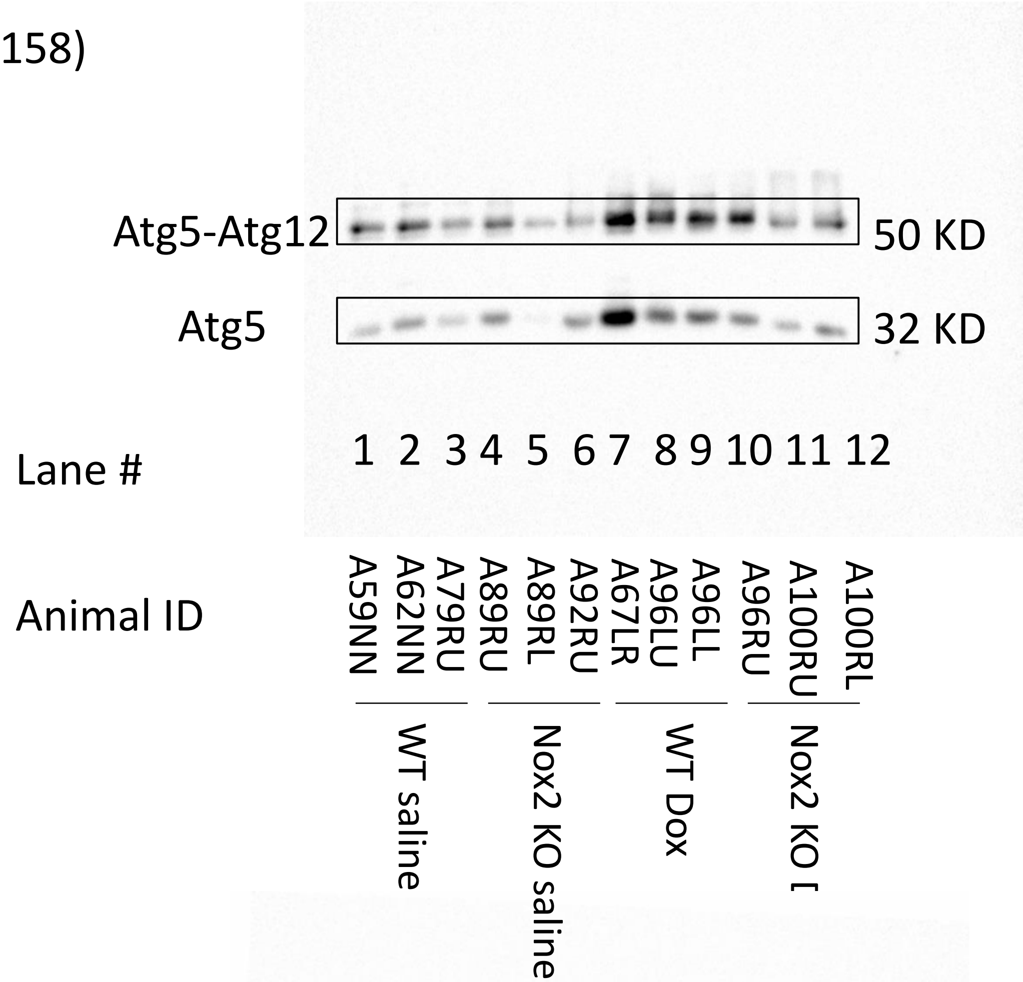

GAPDH (ab8245)

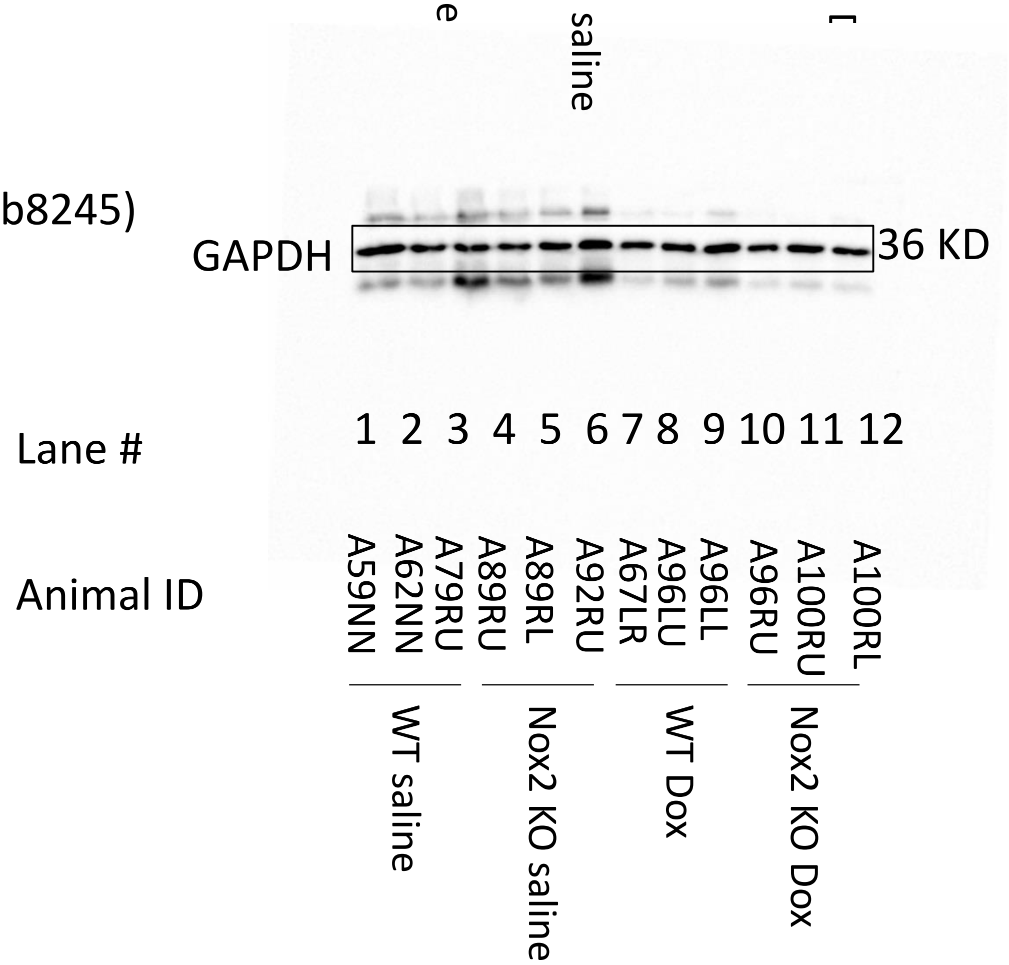

Full-length blots used for Figure 7 A and E.
Area shown in figures are indicated by boxes.

Full-length blots for Figure 7 B

Atg12 (sc-271688)

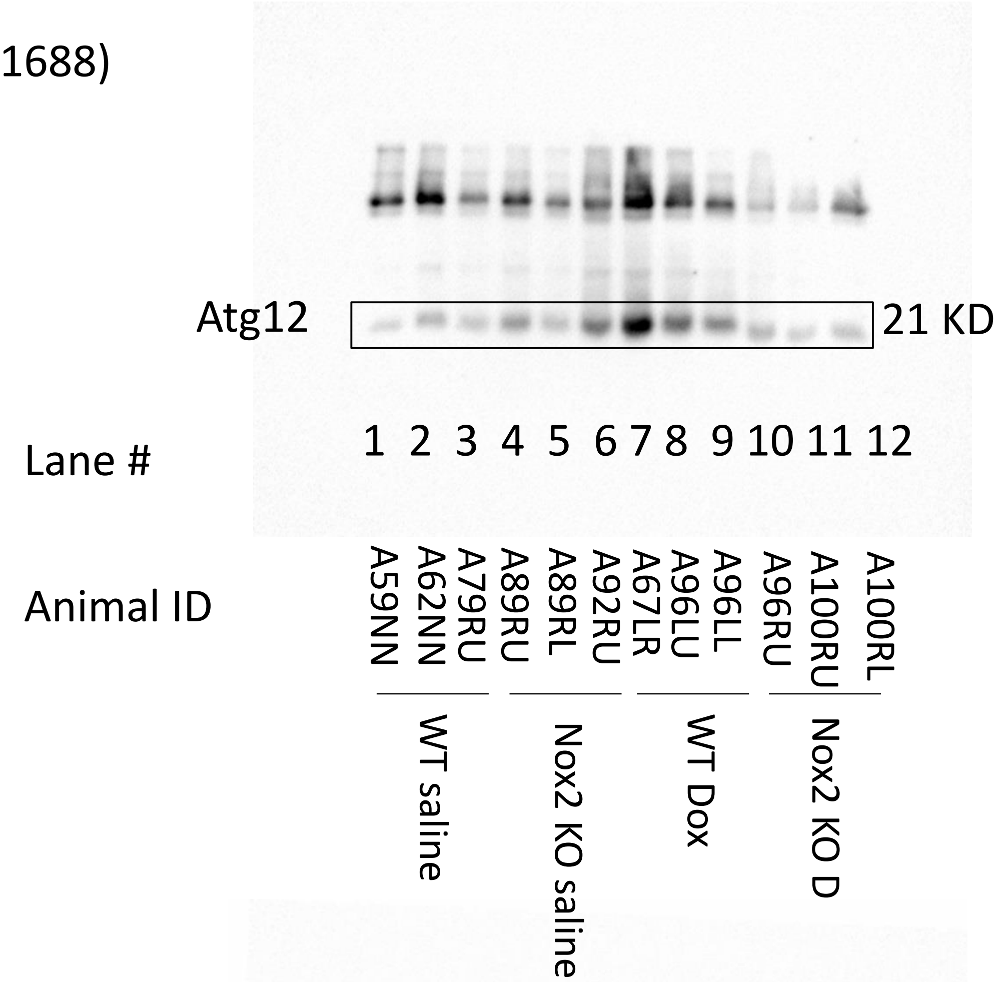

GAPDH (ab8245)

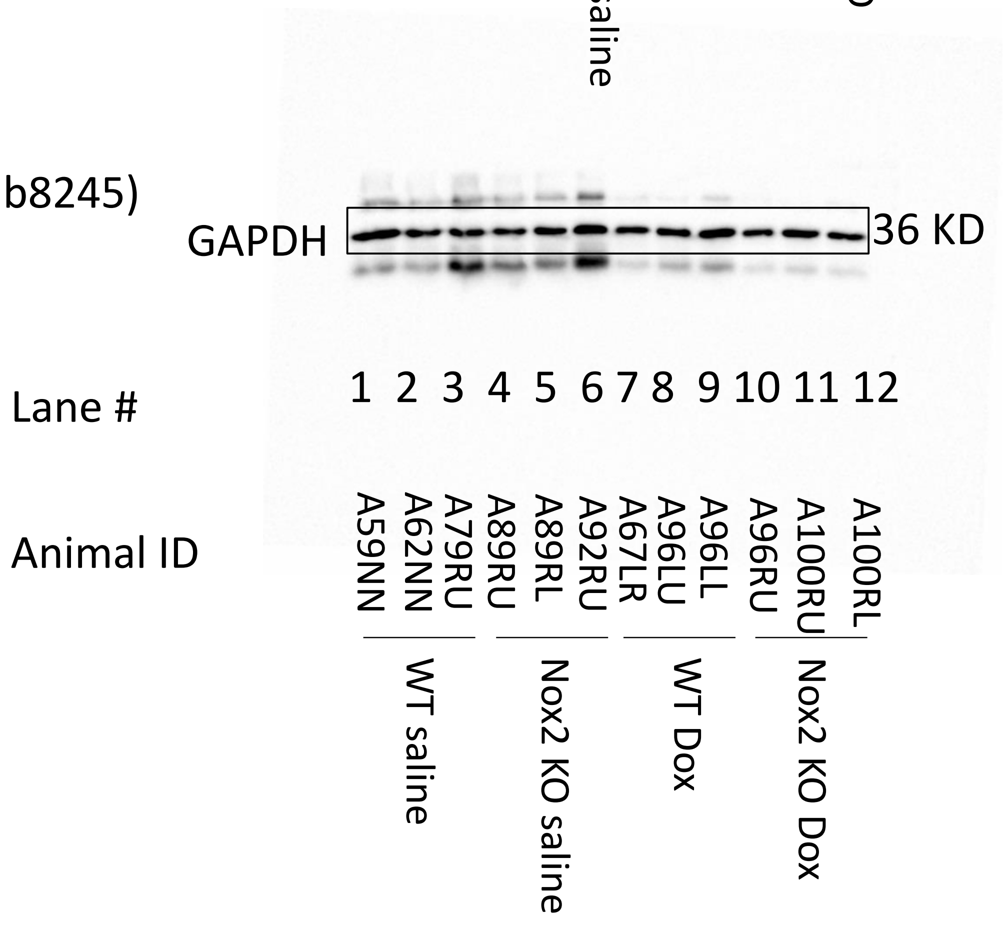

Full-length blots used for Figure 7 B.
Area shown in figures are indicated by boxes.

Full-length blots for Supplementary Figure 1 A

PGP9.5 (ab8189)

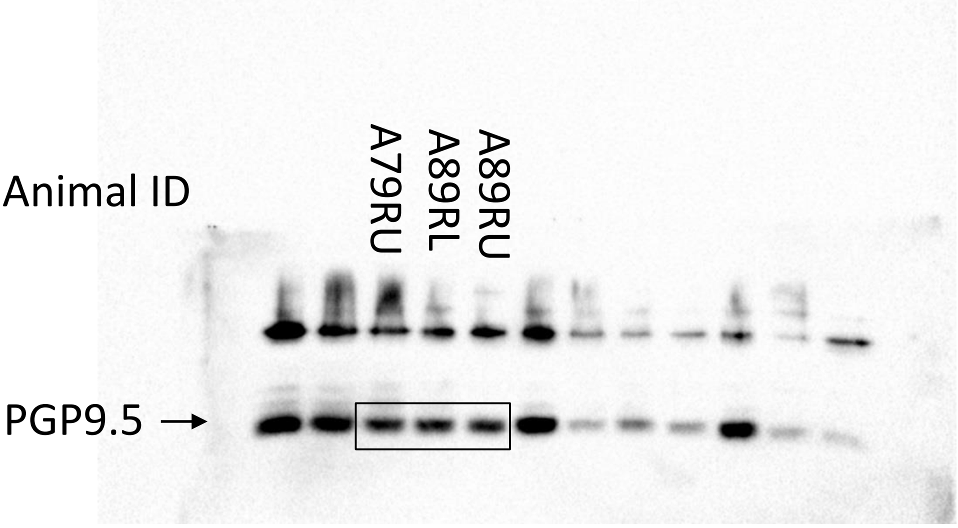

GAPDH (ab8245)

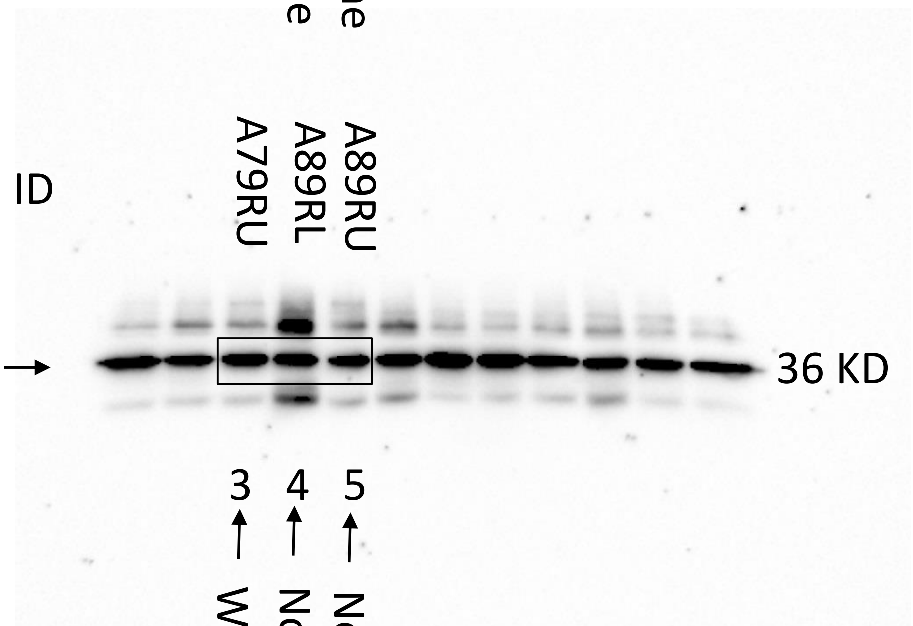

Full-length blots used for Supplementary Figure 1 A.
Area shown in figures are indicated by boxes.

Full-length blots for Supplementary Figure 1 B

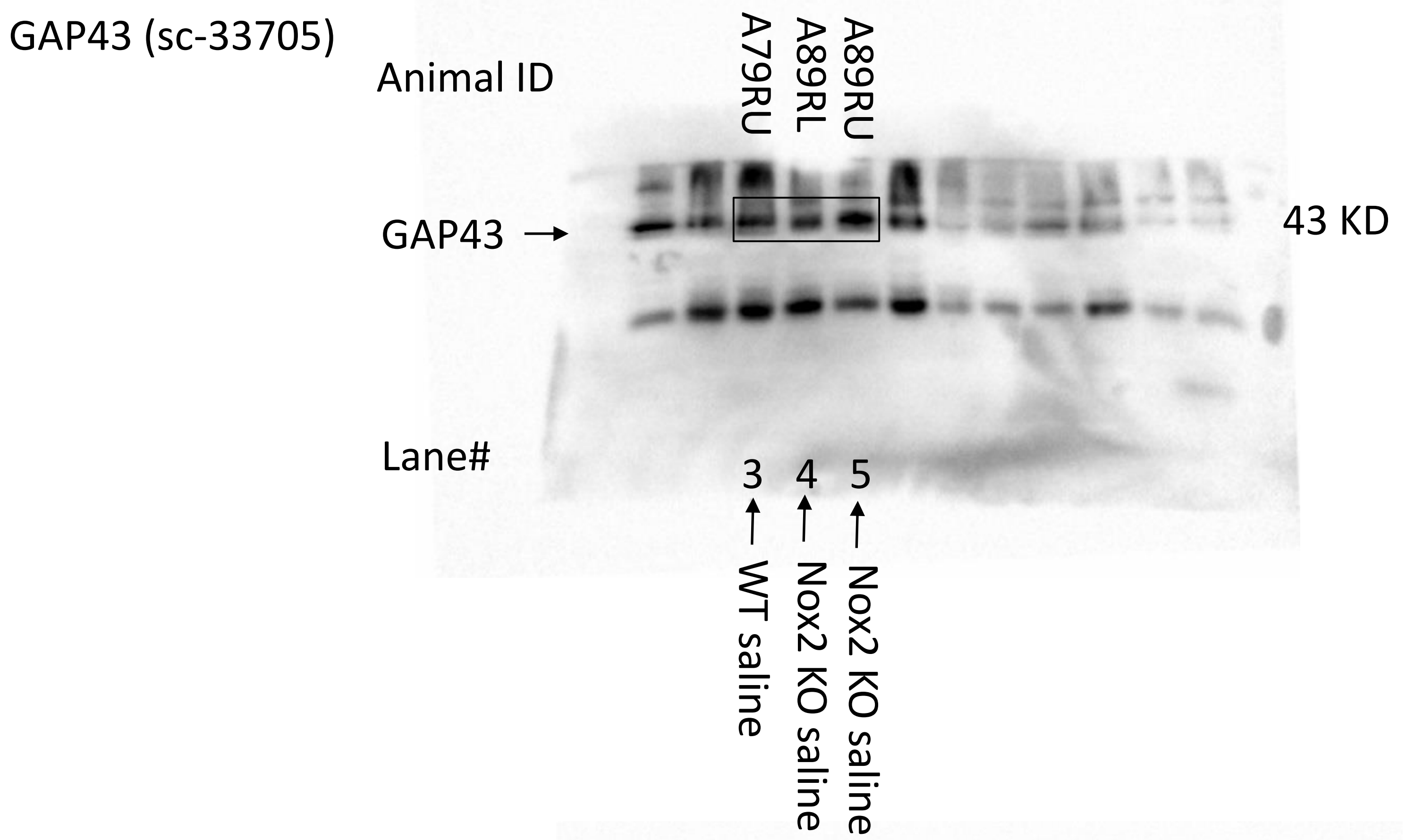

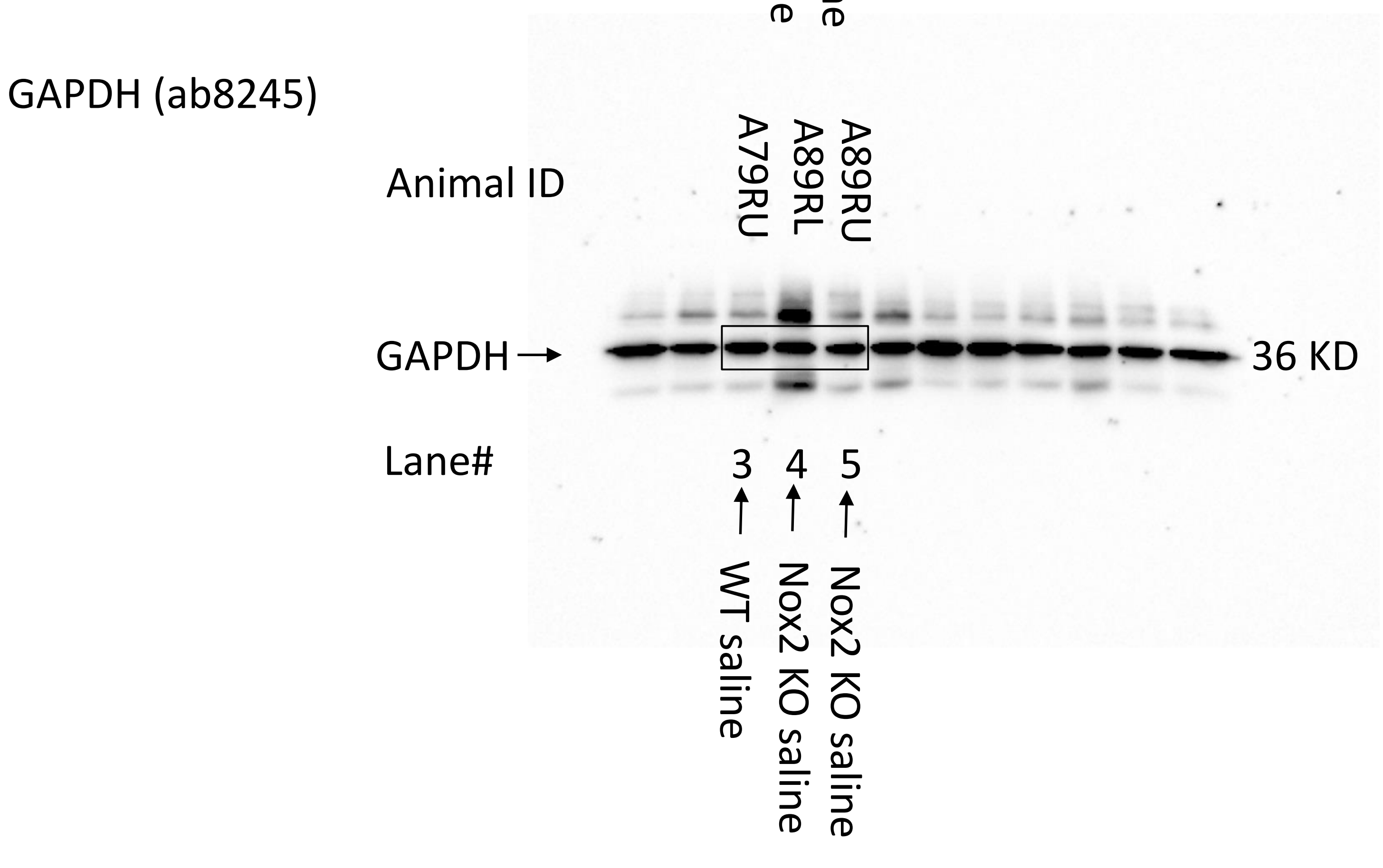

Full-length blots used for Supplementary Figure 1 B.
Area shown in figures are indicated by boxes.

Full-length blots for Supplementary Figure 1 E

Tyrosine hydroxylase (TH)

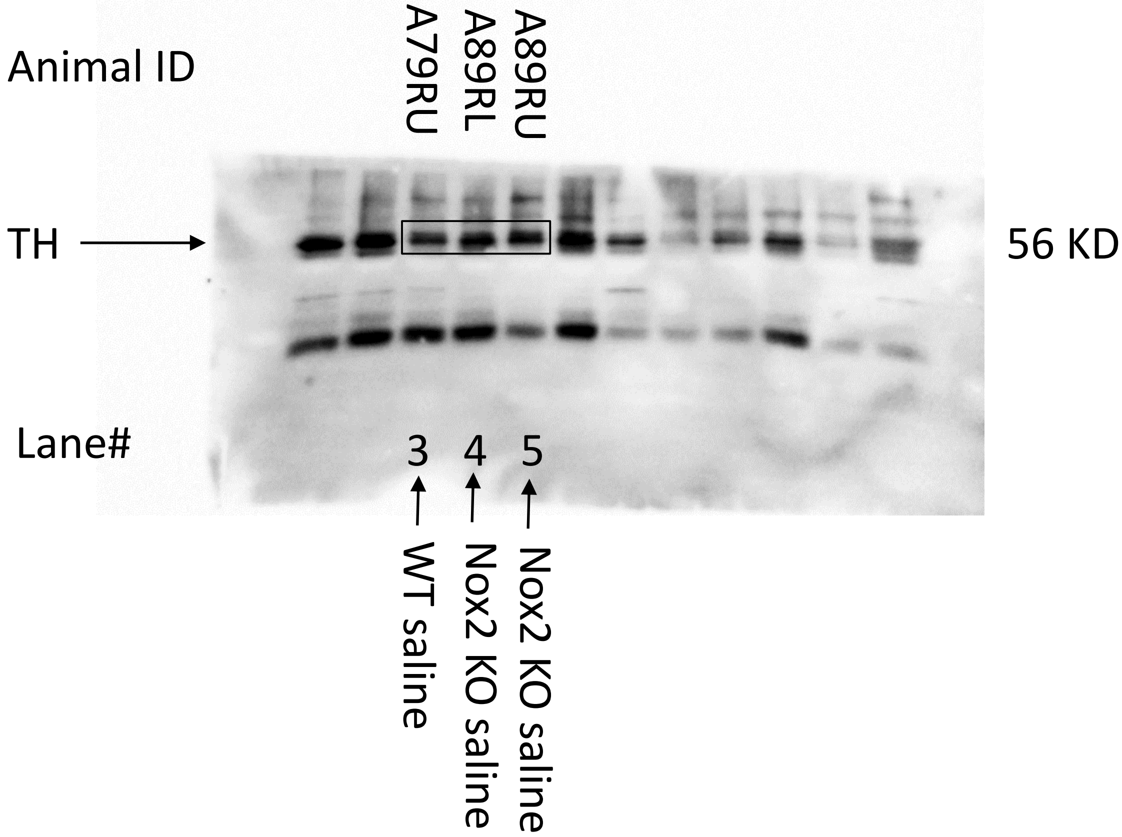

GAPDH (ab8245)

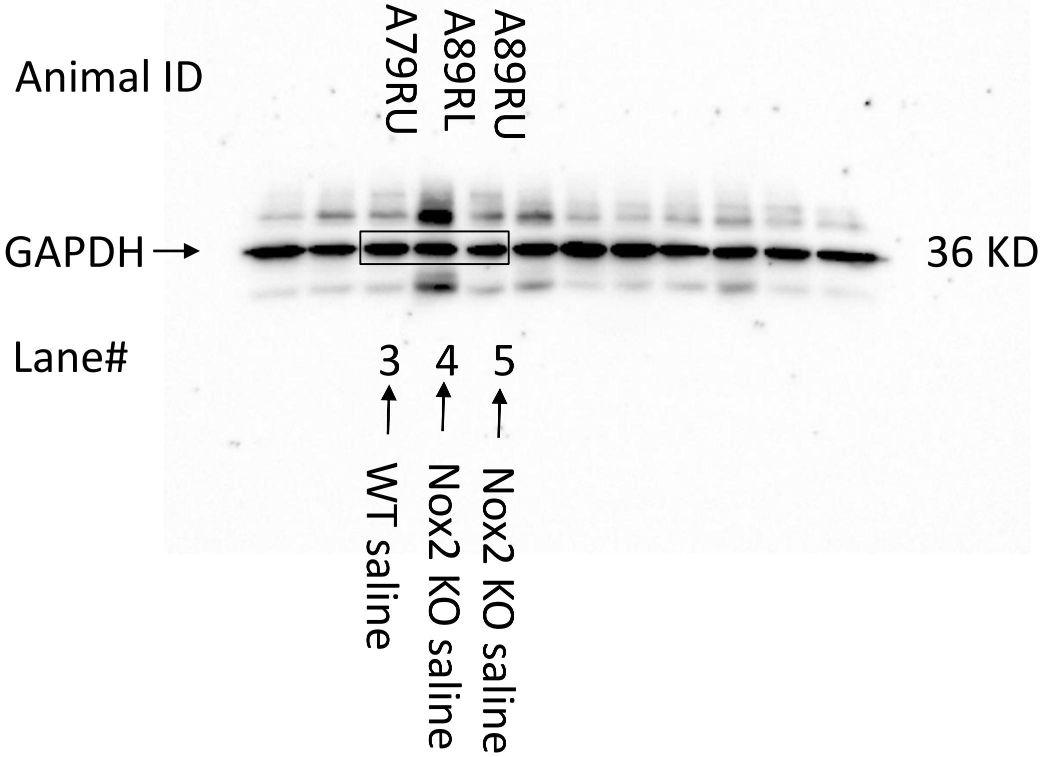

Full-length blots used for Supplementary Figure 1 E.
Area shown in figures are indicated by boxes.

Full-length blots for Supplementary Figure 1 F

Noradrenaline transporter (NET)

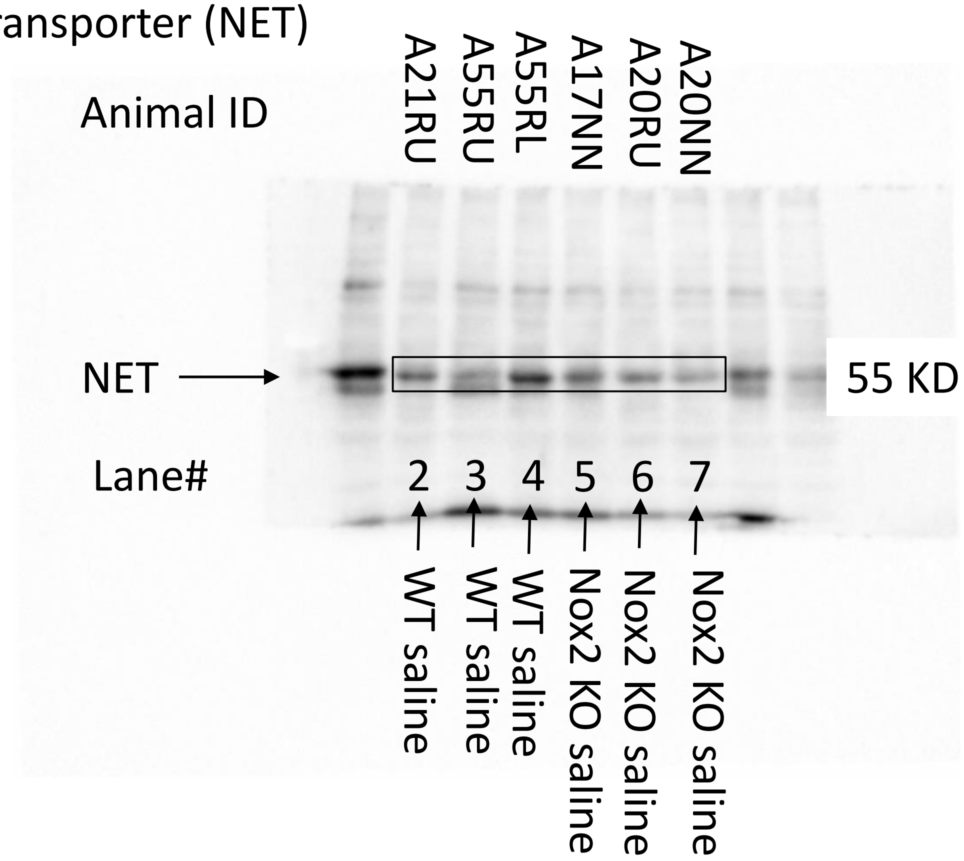

GAPDH (ab8245)

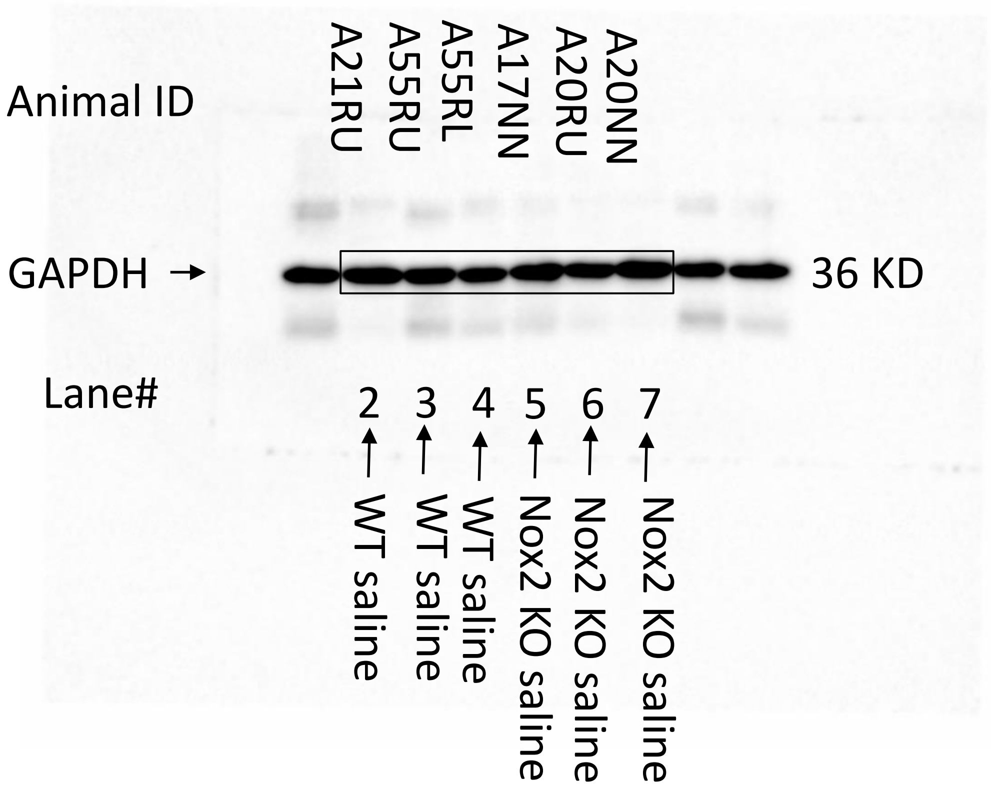

Full-length blots used for Supplementary Figure 1 F.
Area shown in figures are indicated by boxes.

Full-length blots for Supplementary Figure 2 A

ERK (sc-1647)

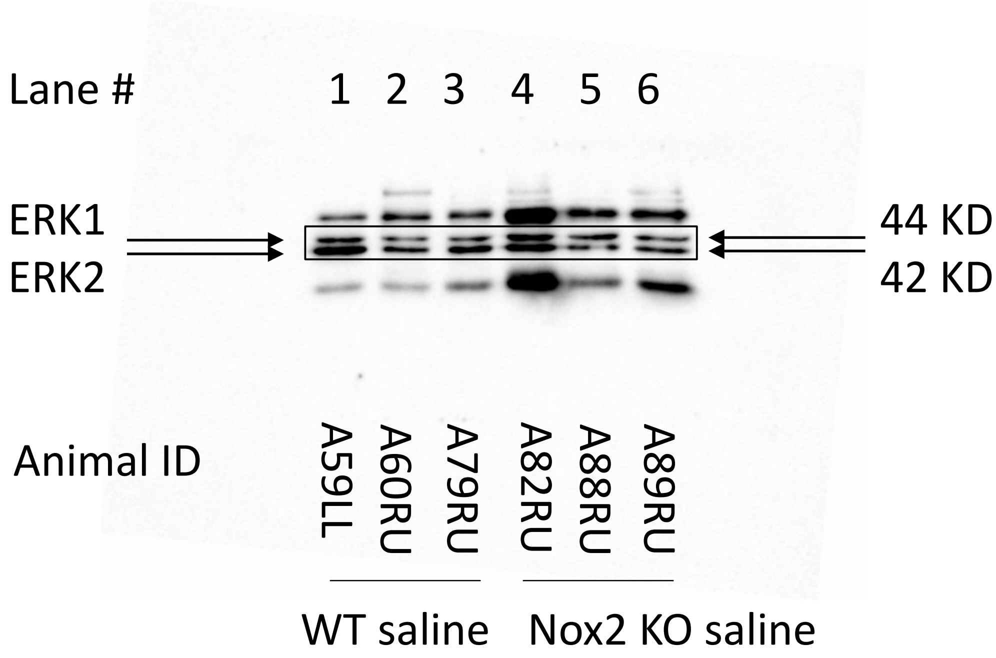

p-ERK (sc-7383)

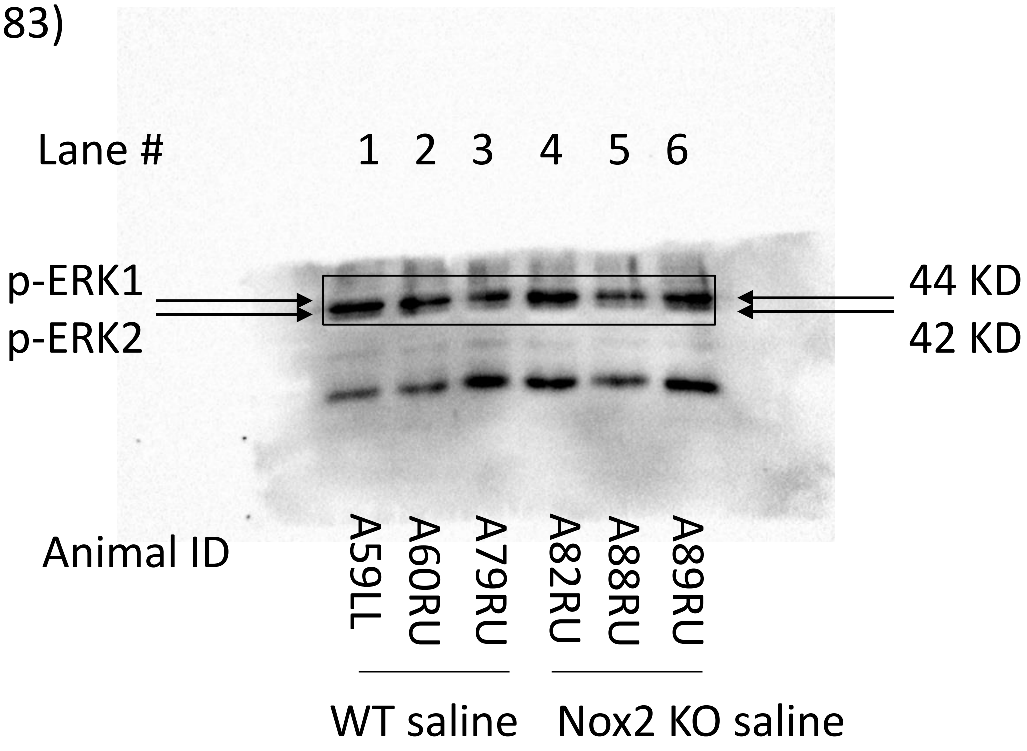

Full-length blots used for Supplementary Figure 2 A.
Area shown in figures are indicated by boxes.

Full-length blots for Supplementary Figure 2 A

Akt (CST9272S)

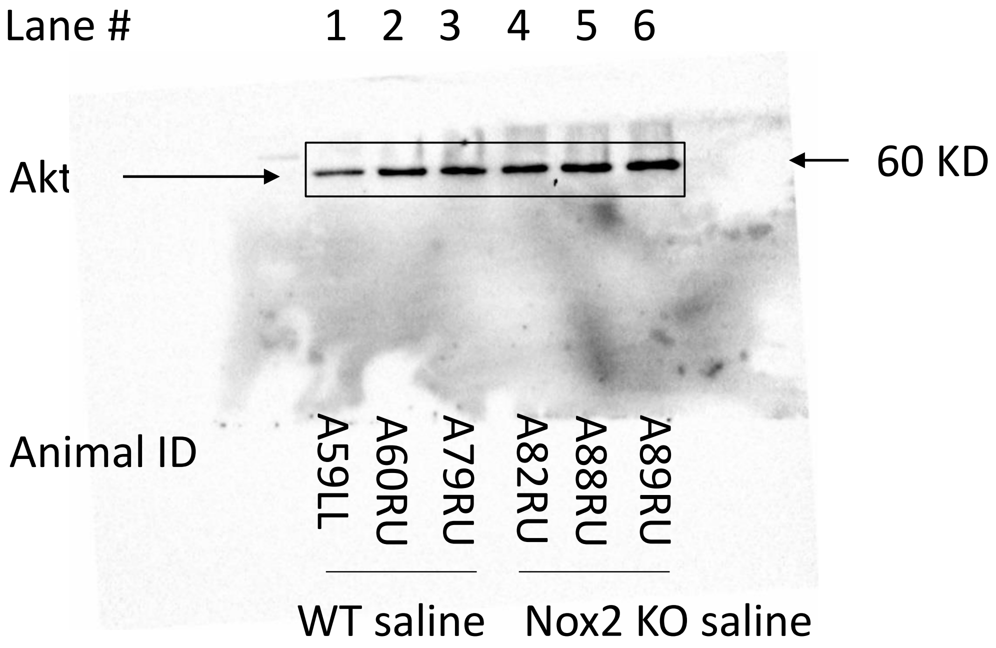

p-Akt (sc-271966)

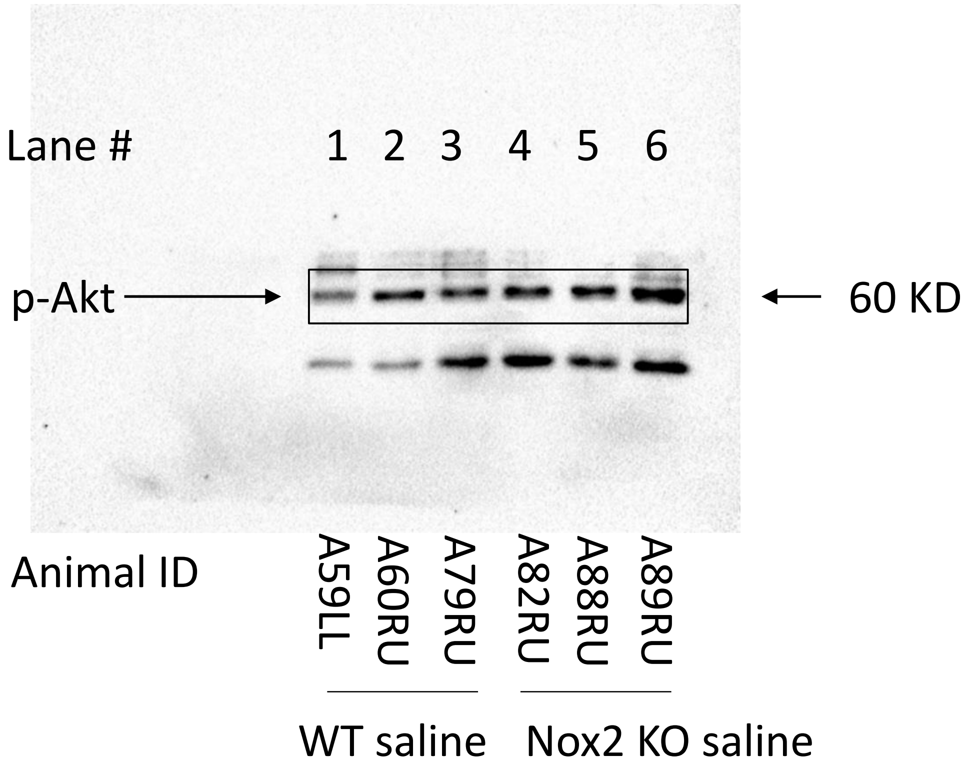

Full-length blots used for Supplementary Figure 2 A.
Area shown in figures are indicated by boxes.

Full-length blots for Supplementary Figure 2 A

S6 (sc-74459)

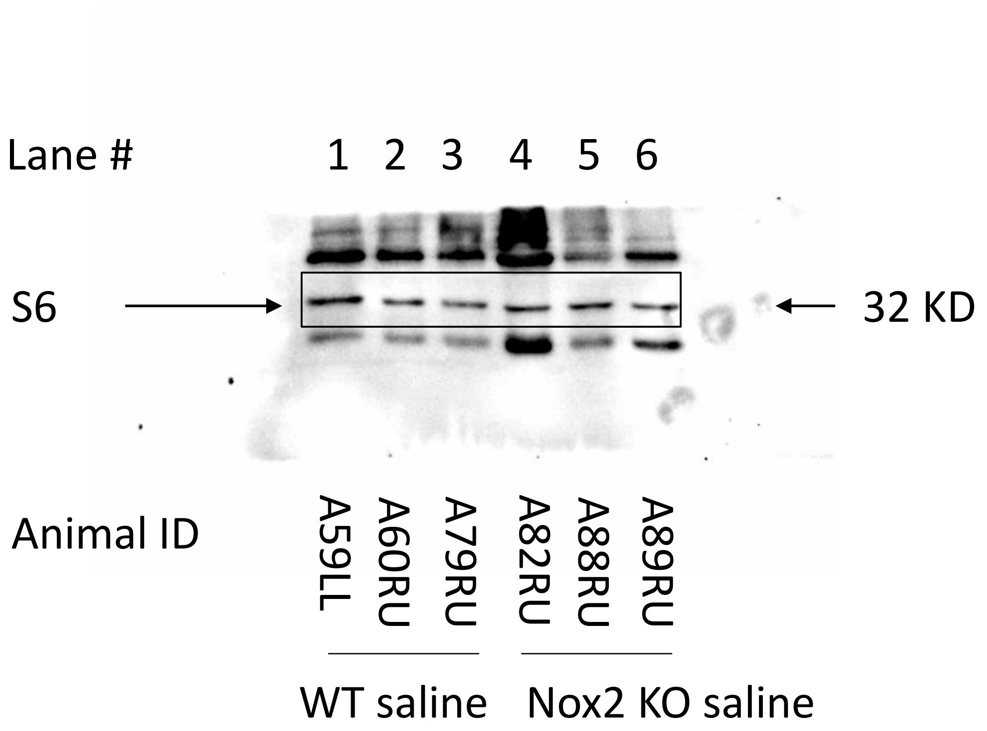

p-S6 (sc-293144)

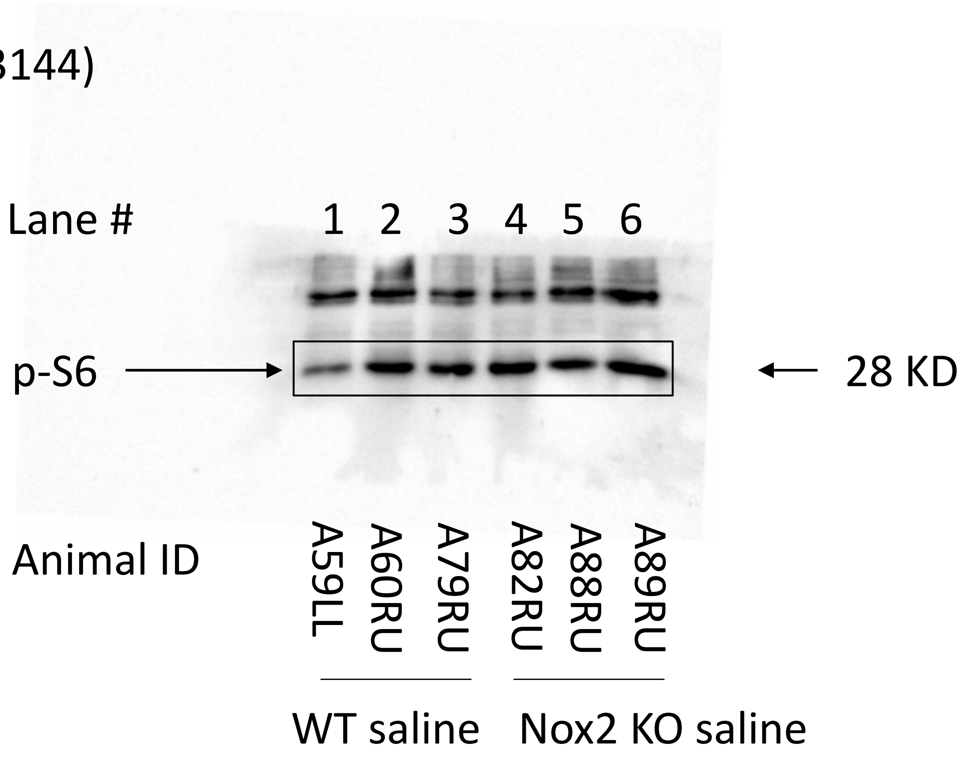

Full-length blots used for Supplementary Figure 2 A.
Area shown in figures are indicated by boxes.

Full-length blots for Supplementary Figure 2 A

GAPDH (ab8245)

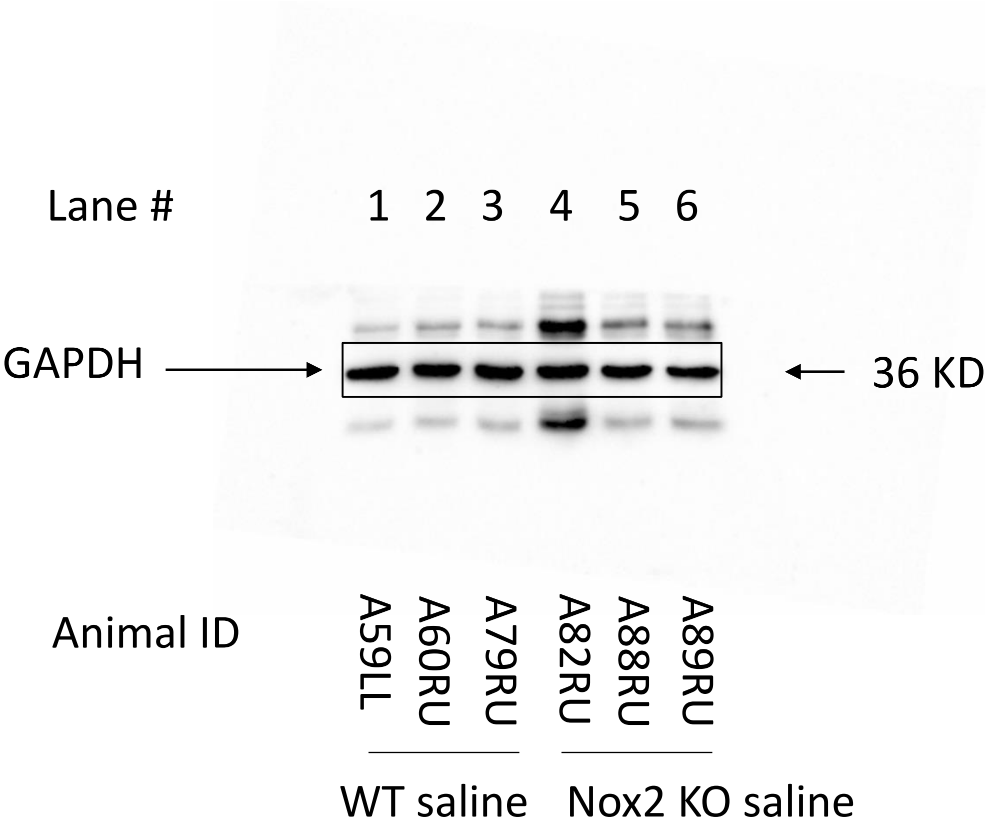

Full-length blots used for Supplementary Figure 2 A.
Area shown in figures are indicated by boxes.

Full-length blots for Supplementary Figure 3 A

LC3 (CST4108)

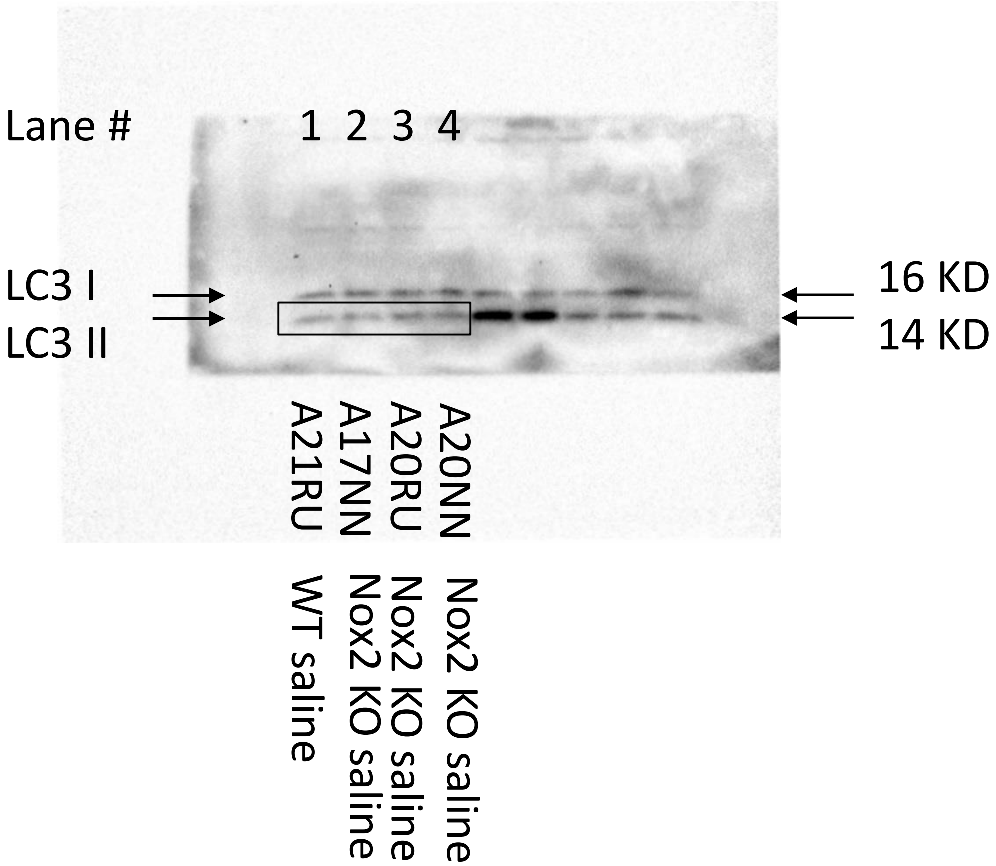

Beclin1 (PD017)

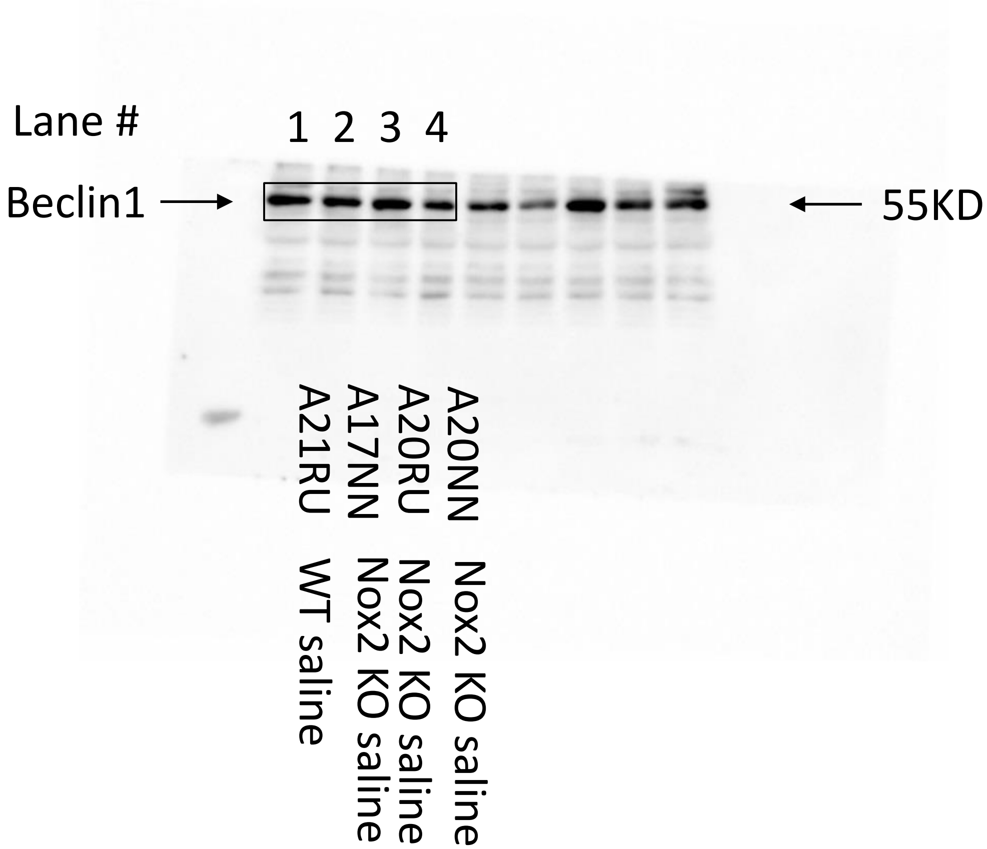

Full-length blots used for Supplementary Figure 3 A.
Area shown in figures are indicated by boxes.

Full-length blots for Supplementary Figure 3 A

GAPDH (ab8245)

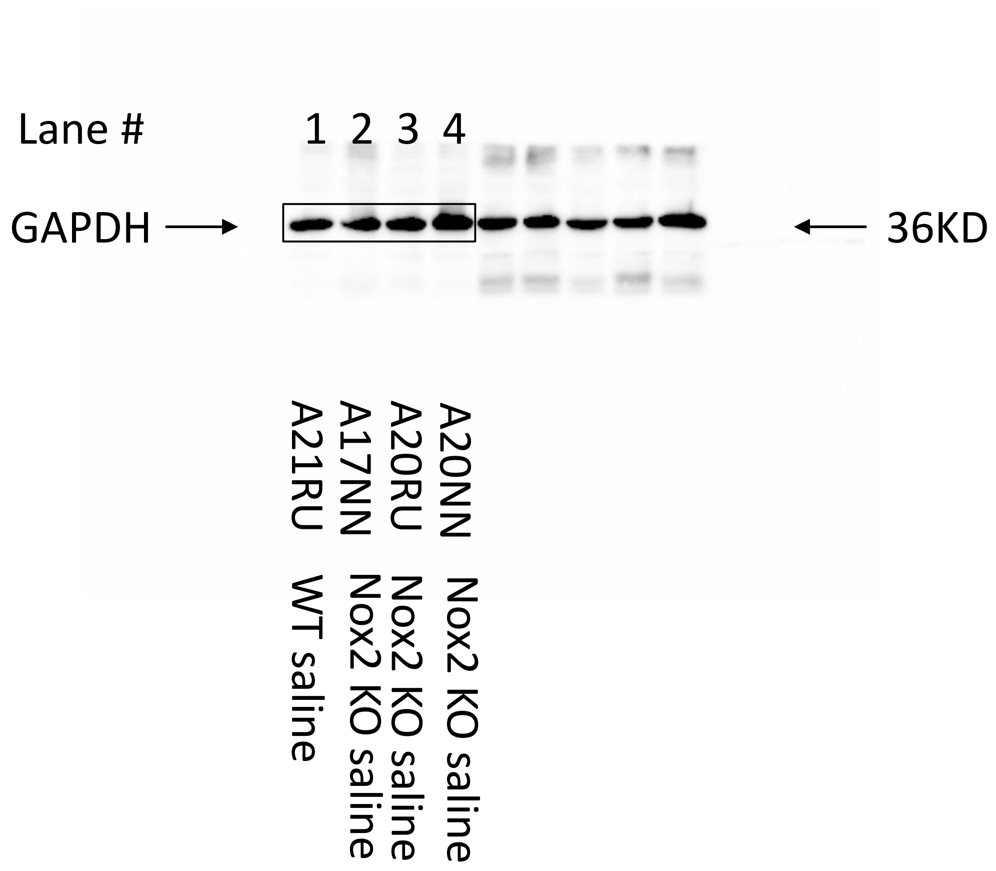

Full-length blots used for Supplementary Figure 3 A.
Area shown in figures are indicated by boxes.

Full-length blots for Supplementary Figure 4 A

LC3 (ab192890)

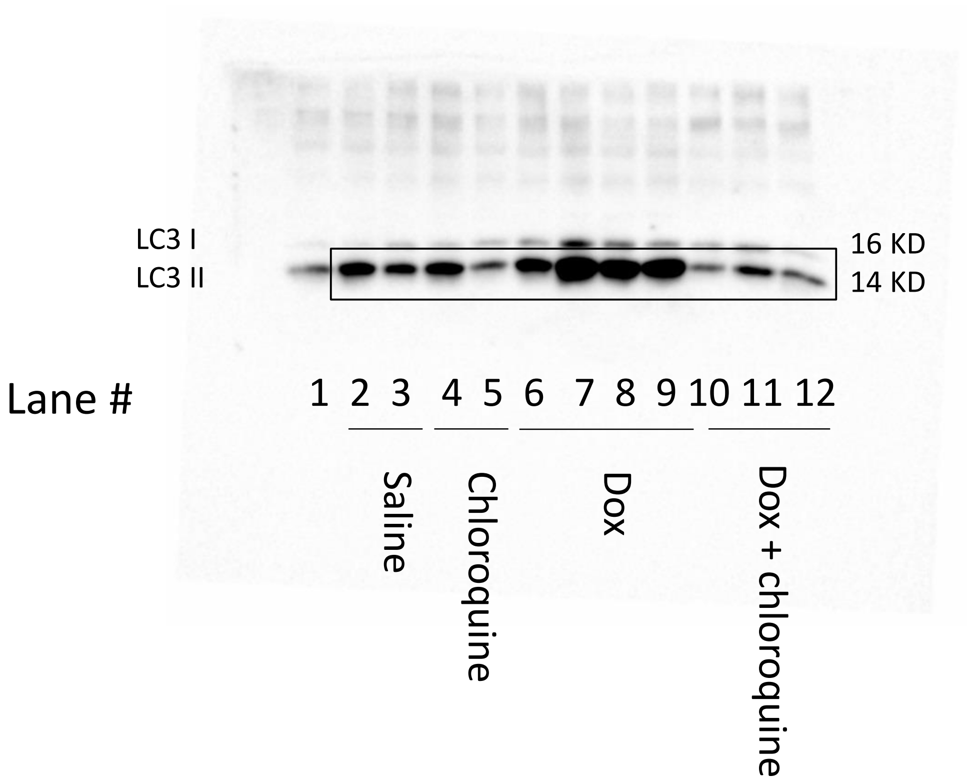

Full-length blots used for Supplementary Figure 4 A.
Area shown in figures are indicated by boxes.

Full-length blots for Supplementary Figure 4 A

GAPDH (ab8245)

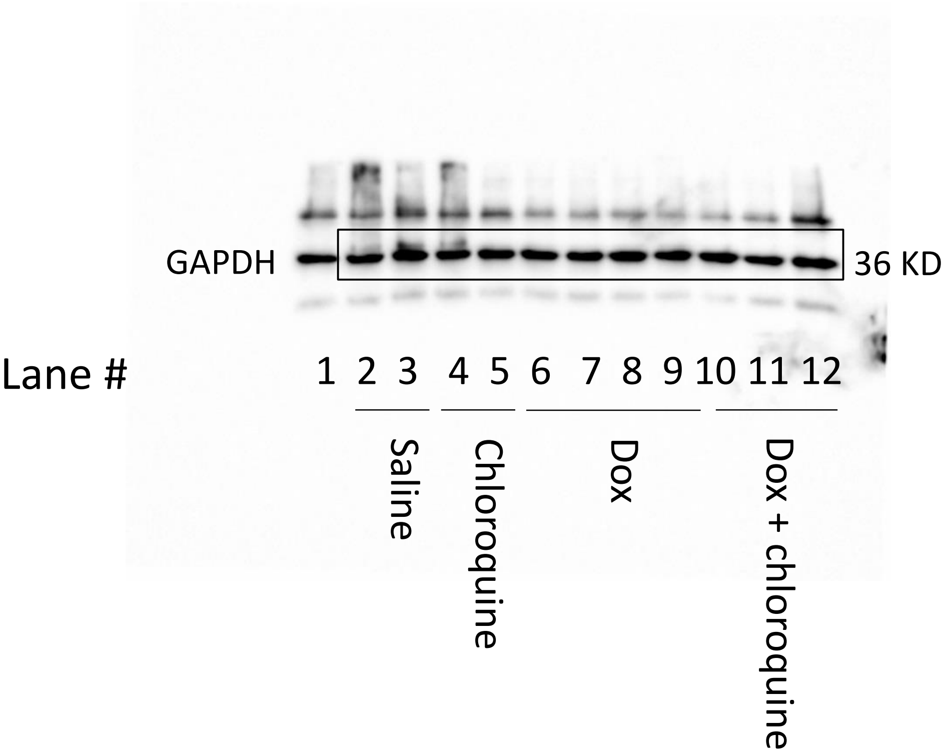

Full-length blots used for Supplementary Figure 4 A.
Area shown in figures are indicated by boxes.

Full-length blots for Supplementary Figure 5 A

Bcl-2 (sc-7382)

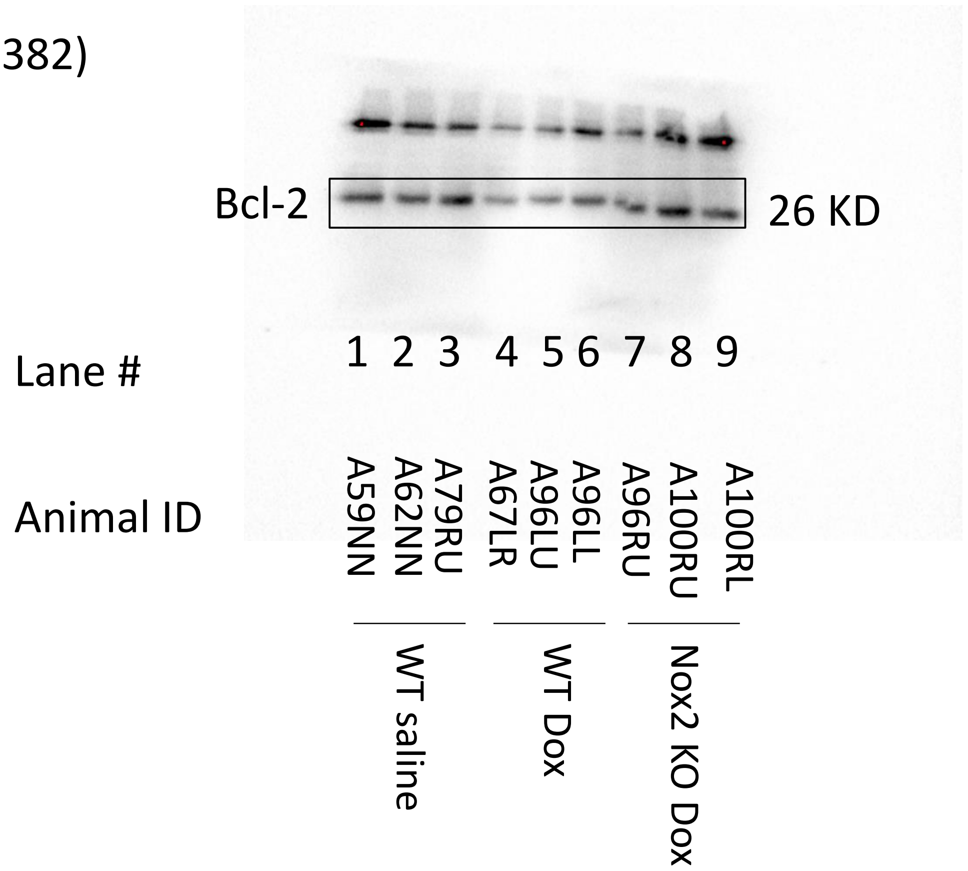

Bax (sc-7480)

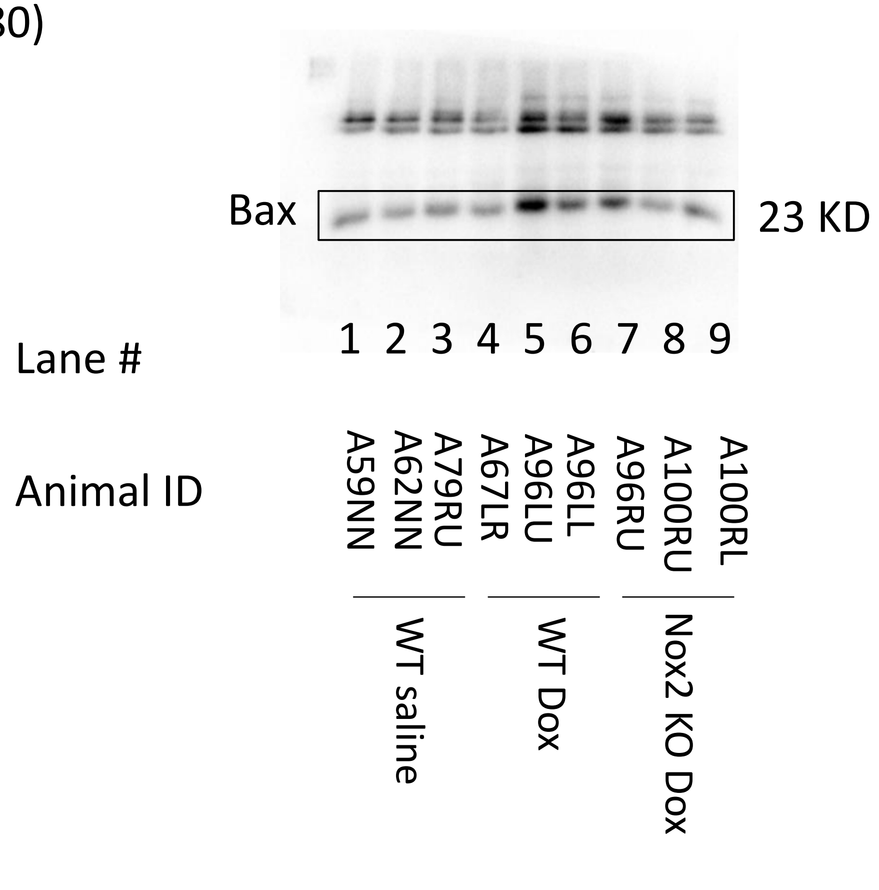

Full-length blots used for Supplementary Figure 5 A.
Area shown in figures are indicated by boxes.

Full-length blots for Supplementary Figure 5 A

Caspase3 (sc-373730)

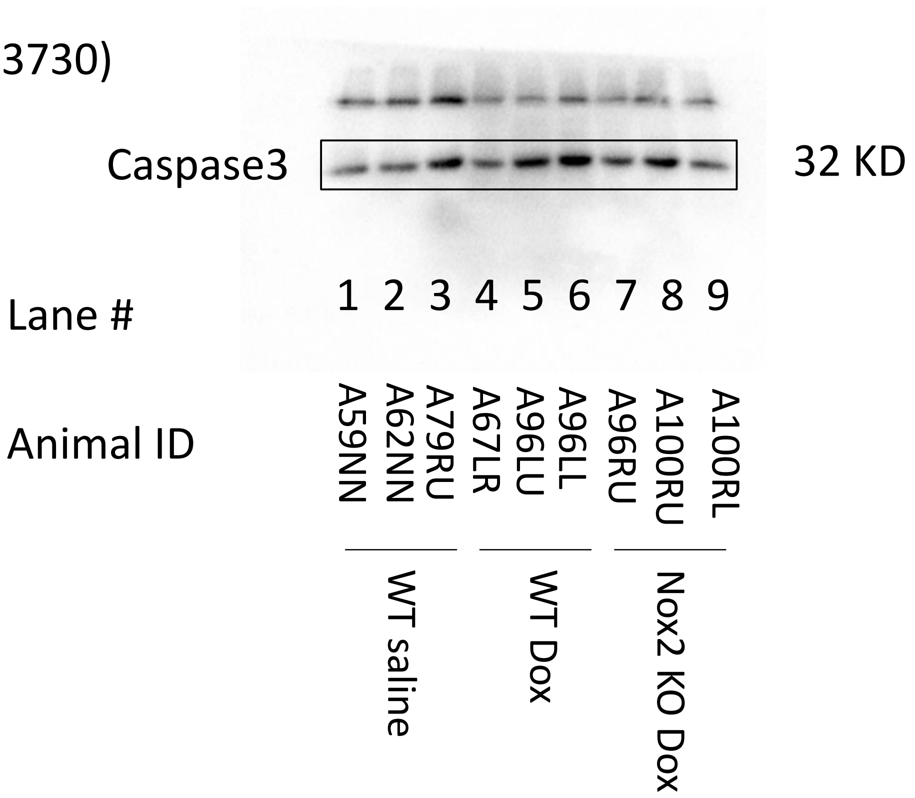

Caspase3 (sc-1224)
Cleaved caspase3

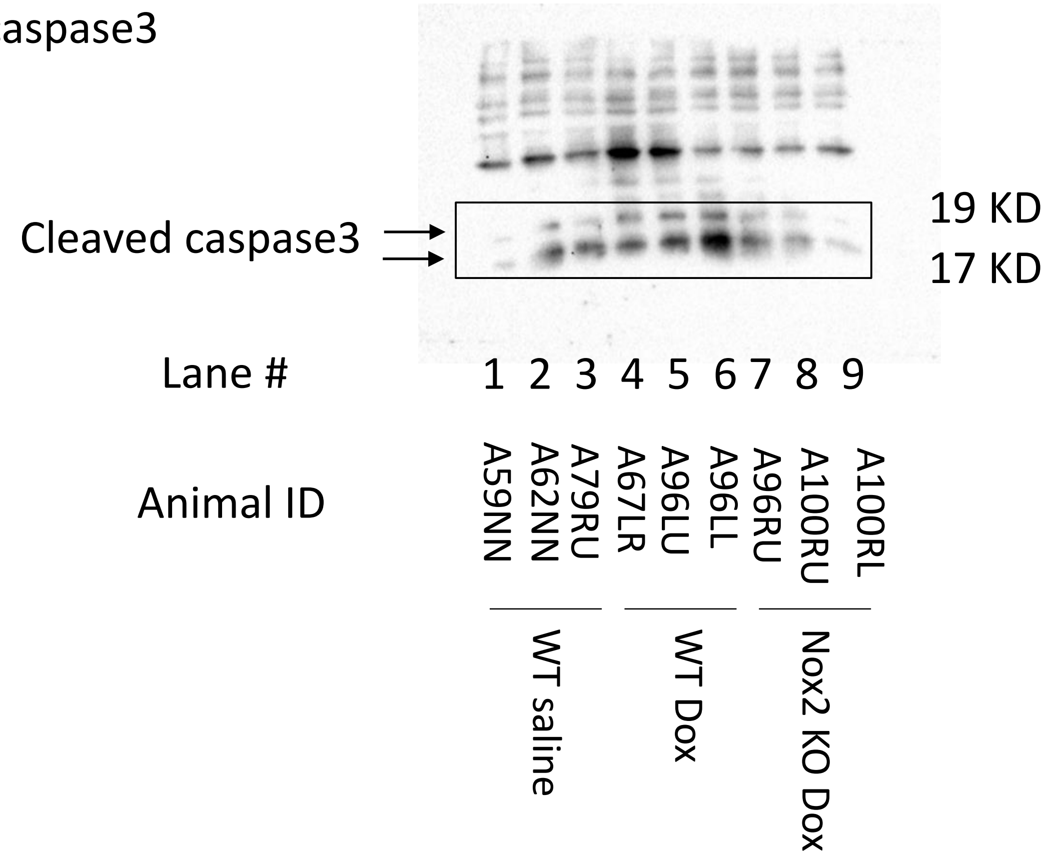

Full-length blots used for Supplementary Figure 5 A.
Area shown in figures are indicated by boxes.

Full-length blots for Supplementary Figure 5 A

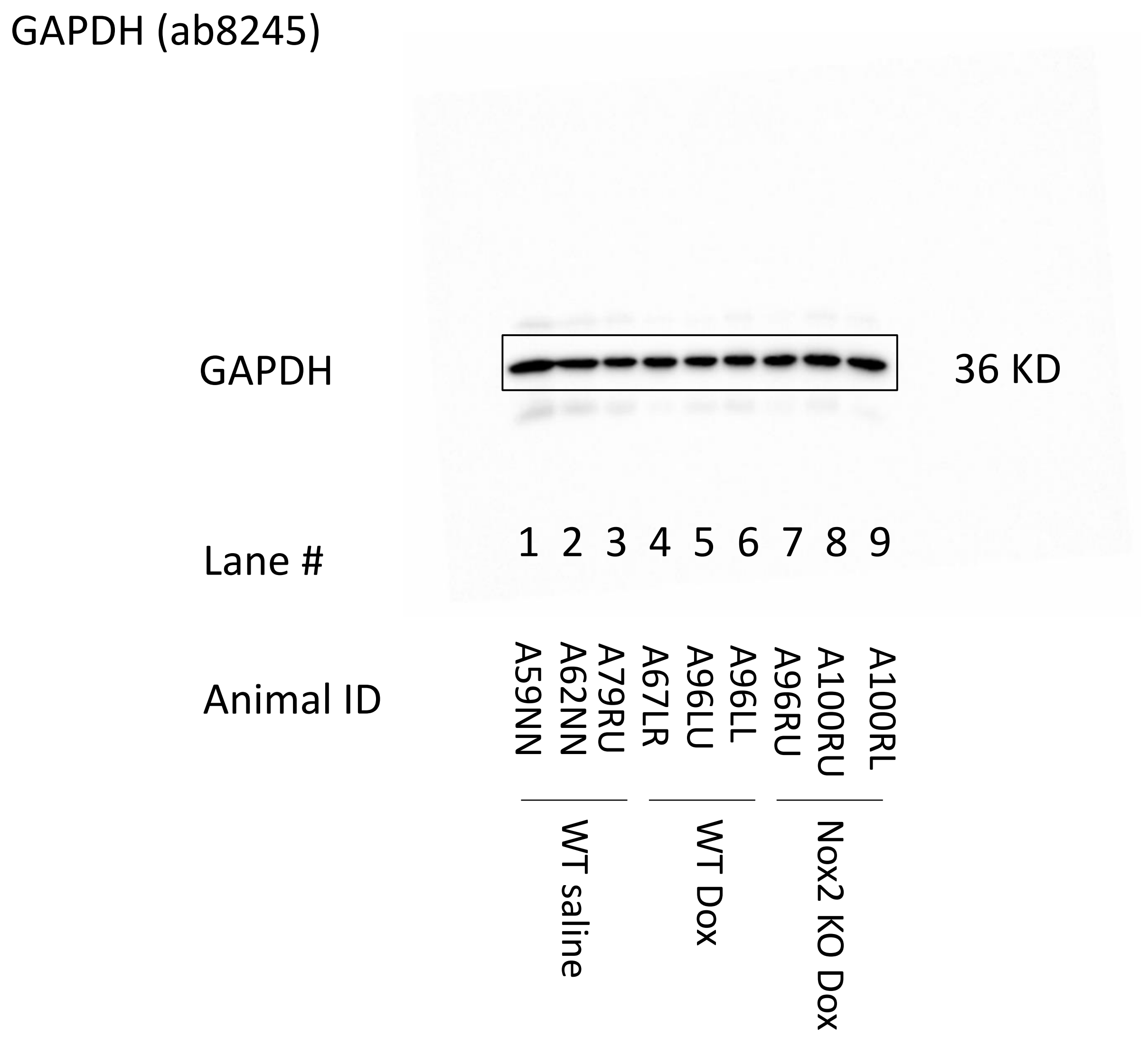

Full-length blots used for Supplementary Figure 5 A.
Area shown in figures are indicated by boxes.
